# Bio-Oriented Synthesis of Novel (S)-Flurbiprofen Clubbed Hydrazone Schiff’s Bases for Diabetic Management: In Vitro and In Silico Studies

**DOI:** 10.3390/ph15060672

**Published:** 2022-05-27

**Authors:** Aftab Alam, Mumtaz Ali, Najeeb Ur Rehman, Saeed Ullah, Sobia Ahsan Halim, Abdul Latif, Zainab, Ajmal Khan, Obaid Ullah, Shujaat Ahmad, Ahmed Al-Harrasi, Manzoor Ahmad

**Affiliations:** 1 Department of Chemistry, University of Malakand, Dir Lower P.O. Box 18800, Khyber Pakhtunkhwa, Pakistan; aftab.alam@uom.edu.pk (A.A.); mumtazali@uom.edu.pk (M.A.); drlatif2016@gmail.com (A.L.); 2 Natural and Medical Sciences Research Center, University of Nizwa, PC 616, Birkat Al Mauz, Nizwa P.O. Box 33, Oman; saidkhan@unizwa.edu.om (S.U.); sobia_halim@unizwa.edu.om (S.A.H.); ajmalkhan@unizwa.edu.om (A.K.); obaidullah@unizwa.edu.om (O.U.); 3 Husein Ebrahim Jamal Research Institute of Chemistry, International Center for Chemical and Biological Sciences, University of Karachi, Karachi 75270, Sindh, Pakistan; 4 College of Chemistry and Materials Science, Hebei Normal University, Shijiazhuang 050024, China; abidkhan4199@gmail.com; 5 Department of Pharmacy, Shaheed Benazir Bhutto University Sheringal, Dir Upper P.O. Box 18800, Khyber Pakhtunkhwa, Pakistan; shujaat@sbbu.edu.pk

**Keywords:** (S)-flurbiprofen, Schiff’s bases, α-glucosidase inhibition, molecular docking

## Abstract

A new series of (S)-flurbiprofen derivatives **4a**–**4p** and **5a**–**5n** were synthesized with different aromatic or aliphatic aldehydes and ketones to produce Schiff’s bases and their structures were confirmed through HR-ESI-MS, ^1^H, and ^13^C-NMR spectroscopy. The α-glucosidase inhibitory activities of the newly synthesized compounds were scrutinized, in which six compounds **5k**, **4h**, **5h**, **4d**, **4b,** and **5i** showed potent inhibition in the range of 0.93 to 10.26 µM, respectively, whereas fifteen compounds **4c, 4g, 4i, 4j, 4l, 4m, 4o, 4p, 5c, 5d, 5j, 5l, 5m, 5n** and **1** exhibited significant inhibitory activity with IC_50_ in range of = 11.42 to 48.39 µM. In addition, compounds **5g, 5f**, **4k**, **4n**, and **4f** displayed moderate-to-low activities. The modes of binding of all the active compounds were determined through the molecular docking approach, which revealed that two residues, specifically Glu277 and His351 are important in the stabilization of the active compounds in the active site of α-glucosidase. Furthermore, these compounds block the active site with high binding energies (−7.51 to −3.36 kcal/mol) thereby inhibiting the function of the enzyme.

## 1. Introduction

Compounds containing at least one carbon–nitrogen double bond with the general formula R_1_R_2_C=NR_3_ are named Schiff’s base [1]. Hydrazones are the bimolecular condensation product of an alkyl or aryl hydrazine and a carbonyl compound which may be an aldehyde or a ketone. Due to their easy synthesis and active pharmacophore group (C=N moiety), hydrazones mostly display good biological and catalytic activities [2]. The Schiff base moiety is a valuable group which is found several bioactive compounds including antioxidant [3,4], antibacterial [5,6], anti-fungal, antiviral [6,7], anti-diabetic [2], anticancer [8], antitumor [9], xanthine oxidase and α-glucosidase inhibitors [10,11,12], anti-inflammatory [13], and anti-convulsant molecules [14]. Khan et al. synthesized and reported the flurbiprofen derivatives as novel α-amylase inhibitors [15], whereas studies have also shown the potent inhibitory potential of Schiff’s bases for cyclooxygenase enzymes (COX-I and COX-II) [15,16].

The non-steroidal anti-inflammatory drugs (NSAIDs) are the most common drugs used in the world to reduce pain, fever, and inflammation due to their analgesic, antipyretic and anti-inflammatory properties [17]. Flurbiprofen is a well-known NSAID, known by the trade name Ansaid, a derivative of phenyl alkanoic acid [18]. This drug is a well-known anti-inflammatory agent because it exerts its effect by blocking prostaglandin synthesis through inhibition of cyclooxygenase enzymes COX-I and COX-II [19]. Being an important class of NSAIDs family, flurbiprofen is extensively used to treat pain, inflammation, acute gout, migraine [20], osteoarthritis [21], soft tissue injuries [22], rheumatoid arthritis [23], postoperative ocular inflammation, herpetic stromal keratitis and periodontal surgery [24]. The hydrazide and hydrazones of flurbiprofen derivatives demonstrated a large number of pharmacological and pharmaceutical activities including anti-microbial, anti-inflammatory, and antioxidant properties, and also inhibit a wide range of bacterial infections [25,26]. In search of biologically active compounds with α-glucosidase inhibitory potential, we have synthesized a new series of flurbiprofen Schiff’s base derivatives.

Diabetes mellitus (DM) is a challenging endocrine disease globally which affects the metabolism of fats, proteins, and carbohydrates [27]. It is a group of metabolic diseases represented by hyperglycemia stemming from either the lack of insulin action or insulin secretion or both [28]. Chronic hyperglycemia is related to dysfunction, long-lasting damage, and malfunction of many organs, such as the eyes, heart, kidneys, blood vessels, and nerves. Primarily, it is sorted into type-I and type-II DM. Type-I DM is caused due to pancreatic cell injury, and sufficient insulin cannot be produced, while type-II DM is caused through the resistance of insulin or less insulin action in the body [29,30]. The inhibition of α-glucosidase slow the digestion of polysaccharides into core monomers, thereby lowering blood sugar levels [31]. In contrast to other hypoglycemic agents that regulate certain biochemical processes, the α-glucosidase inhibitors act locally in the human intestine [32]. However, the clinical uses of these inhibitors are associated with side effects such as abdominal discomfort, diarrhea, and flatulence. Therefore, designing new α-glucosidase inhibitors with no or minimal side effects is of dire need [33,34,35]. 

The main objective of this study was to synthesize various derivatives of the marketed available drug flurbiprofen through simple transformation and to screen the synthesized compounds for their various biological activities. In the past, many derivatives of different drugs have been synthesized and evaluated for different biological activities which showed potent enzyme inhibitory activities, for example, derivatives of secnidazole showed excellent carbonic anhydrase and α-glucosidase inhibitory potential [36], while metronidazole showed potent α-amylase and β-glucuronidase inhibition. Similarly, atenolol derivatives show potent inhibition against the urease enzyme, and piroxicam derivatives were screened for their anti-nociceptive activity with excellent results [37]. Likewise, NSAIDS (ibuprofen and naproxen) are also evaluated for their enzyme inhibitory activities, which showed promising results [36].

**Figure 1:**
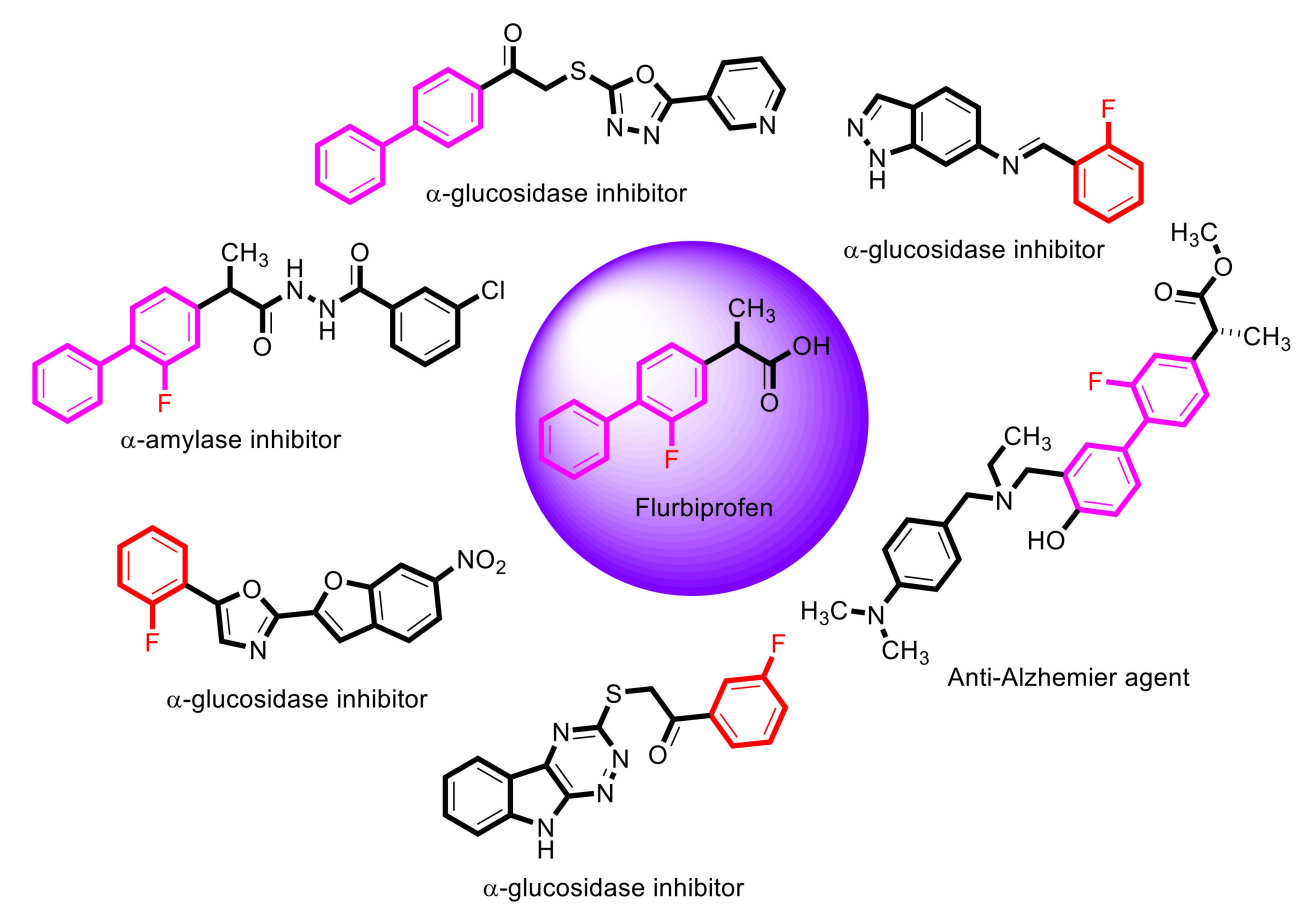
Reported α-glucosidase inhibitors [15,28,29].

Recently, Momin khan et al. reported oxadiazole-based derivatives of flurbiprofen and screened them for their in vitro α-amylase inhibitory potential with excellent results [15]. Similarly, compounds containing biphenyl rings were checked for their anti-Alzheimer and α-glucosidase inhibitory activities and considered as lead molecules for diabetes mellitus type-II (Figure 1) [37]. Likewise, Bushra et al. and Taha et al. reported a number of compounds with fluorine atoms attached to the benzene ring with excellent α-glucosidase inhibitory potential and confirmed that the fluorine group is responsive to the strength of compounds [38]. Flurbiprofen also has a fluorine group attached to the benzene ring; therefore, we planned to synthesize derivatives of flurbiprofen for their α-glucosidase inhibitory activity.

## 2. Results and Discussion

### 2.1. Chemistry

**Scheme 1:**
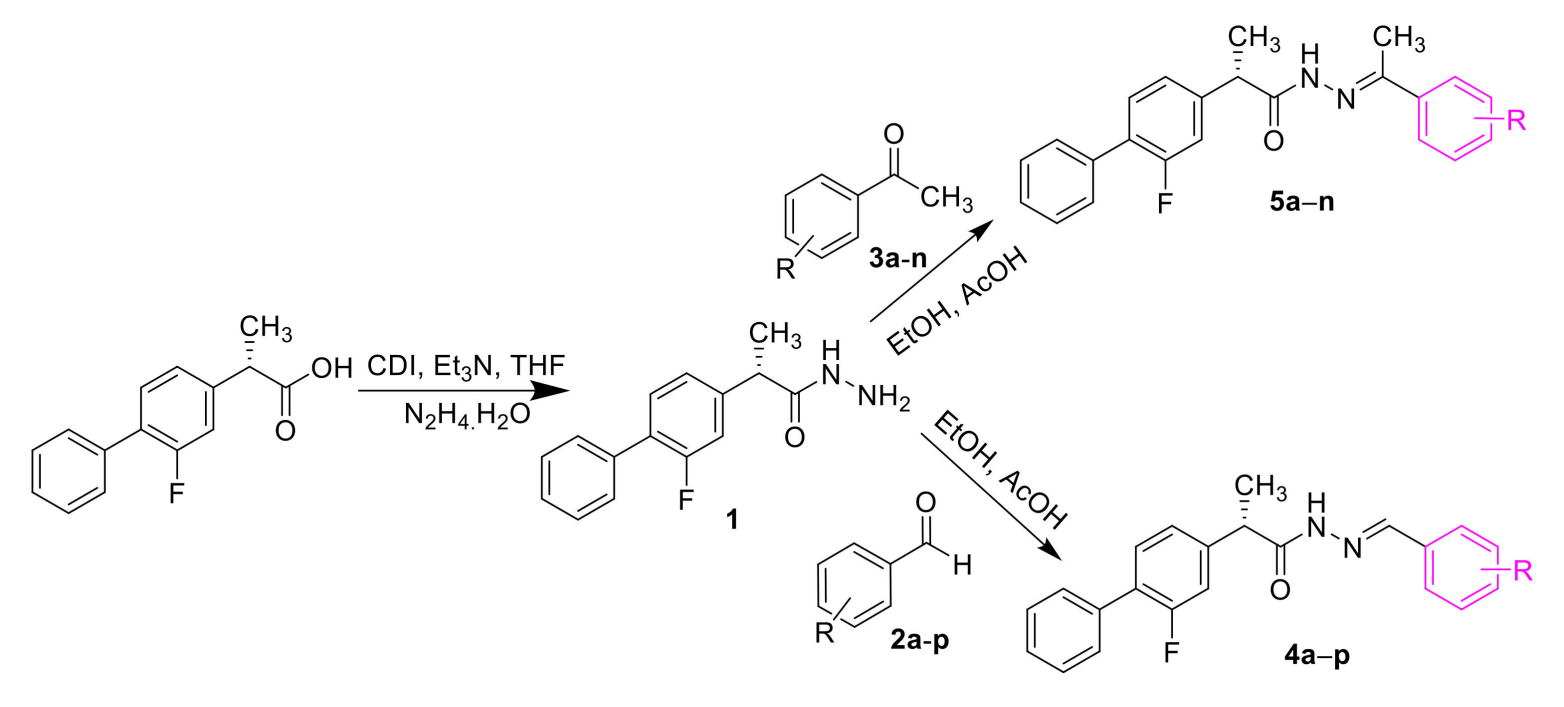
Schiff’s base derivatives of flurbiprofen.

In continuation of our efforts to discover pharmacologically potent α-glucosidase inhibitors, thirty Schiff base derivatives of flurbiprofen were successfully synthesized through multi-step reactions. Flurbiprofen was reacted with hydrazine hydrate in the presence of CDI (1, 1′-carbonyldiimidazole) and triethylamine (Et_3_N) in THF solvent. The hydrazide was further reacted with various substituted aliphatic/aromatic aldehydes or ketones in the presence of acid using ethanol solvent to give the desired Schiff’s base derivatives of flurbiprofen (Scheme 1).

### 2.2. In Vitro α-Glucosidase Inhibitory Activity

The synthesized compounds were tested for in vitro α-glucosidase inhibitory potential and compared with standard drug acarbose. All the compounds showed α-glucosidase inhibitory activity except five compounds (**4a**, **4e**, **5a**, **5b**, and **5e**). In this series, compounds **4b**, **4d, 4h, 5k, 5h** and **5i** were found potent against α-glucosidase as compared to the standard inhibitor, acarbose (IC_50_ = 875.75 ± 1.24 µM). Compound **5k** (IC_50_ = 0.93 µM), was the most potent inhibitor followed by **4h** (IC_50_ = 1.52 µM), **5h** (IC_50_ = 3.41 µM), **4d** (IC_50_ = 4.77 µM), **4b** (IC_50_ = 7.61 µM), and **5i** (IC_50_ = 10.26 µM), (Figure 1, Table 1). Furthermore, compounds **4g**, **5m**, **4i**, **5n**, **5l**, **4l**, **4p**, **4j**, **4m**, **4o**, **5j**, **1**, **5c**, **5d**, and **4c** also demonstrated significant inhibitory activity (IC_50_ value ranging from 11.42 to 48.39 µM) against α-glucosidase, while five compounds [**5g**, (IC_50_ = 68.75 µM), **5f** (IC_50_ = 136.36 µM), **4k** (IC_50_ = 146.78 µM), **4n** (IC_50_ = 147.26 µM), and **4f** (IC_50_ = 426.82 µM)] showed moderate to least inhibitory activities (Table 1).

**Figure 2:**
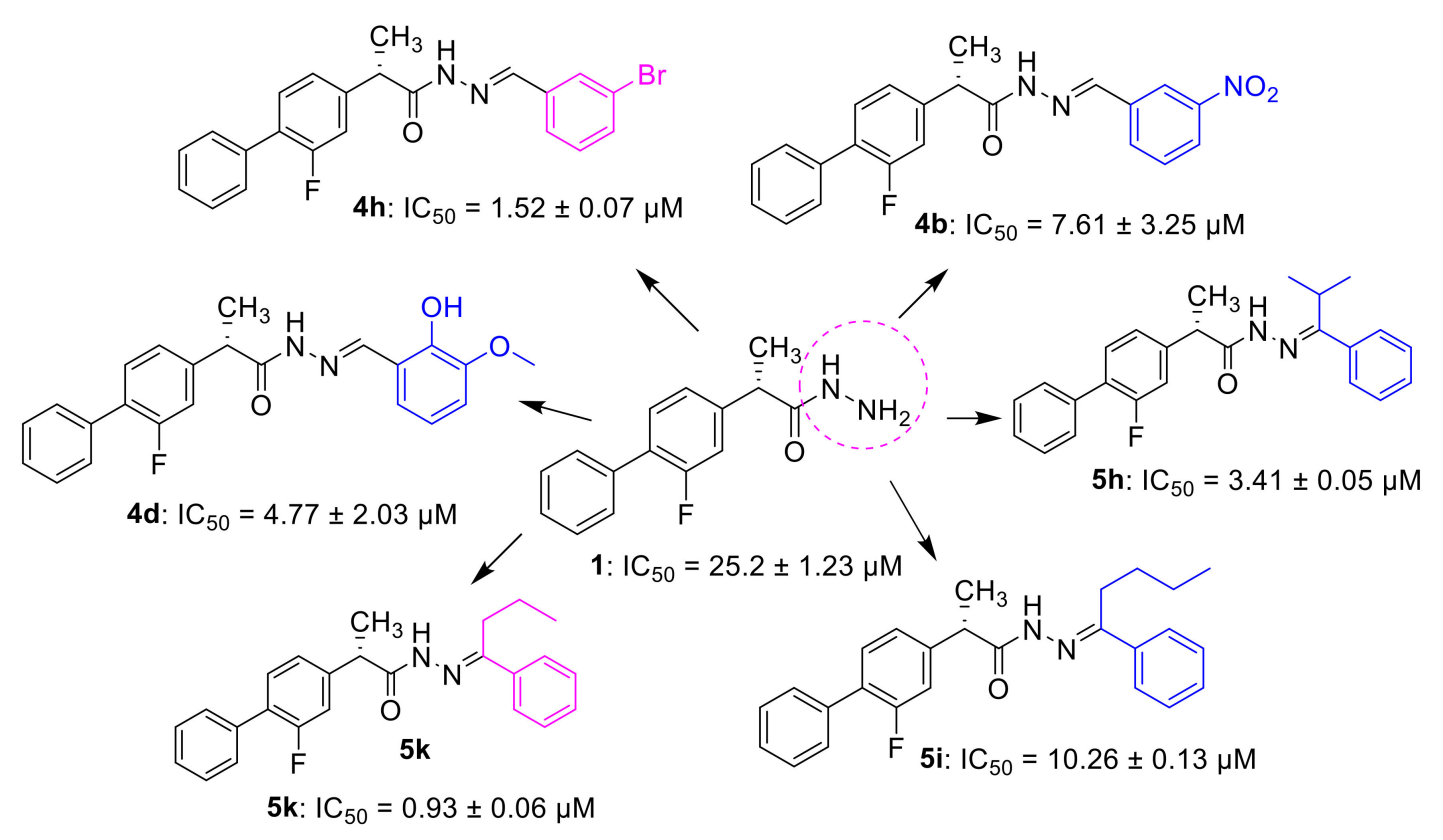
Substitution influence on the Schiff’s base derivatives of flurbiprofen.

The structure–activity relationship (SAR), for better understanding, was discussed by analyzing the changes in position and nature of attached substituents (R) on the benzene ring. In the case of the aldehydes series, compound **4h** was the most active inhibitor of α-glucosidase having IC_50_ value of 1.52 ± 0.07 μM (Figure 2), ~580 times stronger than acarbose as the standard inhibitor (Figure 2). The most potent activity of **4h** could be due to the presence of bromine substituent at the meta position of the benzene ring. The decrease in α-glucosidase inhibition of **4g** (IC_50_ = 11.42 ± 1.09 μM) may be likely due to the presence of bromine at the para substitution and additional substituent (fluorine) at the ortho position of the benzene ring. Similarly, a further slight decline in the inhibition of **4i** (IC_50_ = 13.20 ± 2.47 μM) could be due to the presence of additional bromine at the ortho substituent and hydroxyl group at the para position of the benzene ring. Comparing **4d** (IC_50_ = 4.77 ± 2.03 μM) with **4j** (IC_50_ = 18.71 ± 0.25 μM) and **4m** (IC_50_ = 19.84 ± 1.89), the higher activity of the 4d might be due to the presence of hydroxyl and methoxy groups at ortho and meta positions, respectively, attached to the benzene ring. The compounds (**4j** and **4m**) attributed almost the same inhibition, while the lower activity of the compound **4f** (IC_50_ = 426.82 ± 4.63 μM) suggested that the –OH group at ortho position and –OCH_3_ at ortho and meta substitution on the benzene ring may interact well with the α-glucosidase enzyme compared to para (Table 1). The slight decline in activity of **4c** (IC_50_ = 48.39 ± 3.75 μM) is likely due to the substitution of second –OH at para position, indicating the interaction of –OH at para substitution with the screen enzyme as well. Comparing straight-chain alkanes, compounds **4l** (IC_50_ = 17.36 ± 1.68 μM), **4o** (IC_50_ = 22.72 ± 1.46 μM), displayed outstanding inhibition than **4k** (IC_50_ =146.78 ± 0.86 μM), indicating that n-hexyl and octyl groups might have better interaction with the enzyme compared to butyl in the aldehyde series. Compound **4p** revealed promising activity with IC_50_ value of 18.61 ± 0.14 μM having a carboxylic group at the para substitution of the benzene ring, while **4n** (IC_50_ = 147.26 ± 2.78 μM, naphthalene substituent), determined good inhibition compared to the standard.

Similarly, in the case of ketone series, **5k** attributed excellent inhibitory activity ((IC_50_ = 0.93 ± 0.06 μM), ~940 times stronger) compared to the standard, which could be attributed due to the presence of benzene ring on one side and propyl group on the other side of the attached moiety.

Comparing the most active compound **5k** with **5h** and **5i**, a decrease in inhibition of the can be attributed to the replacement of propyl with isopropyl (**5h**, IC_50_ = 3.41 ± 0.05 μM) and butyl (**5i**, IC_50_ = 10.26 ± 0.13 μM) on one side of the attached moiety. On the other side, comparing compounds **5h** and **5i**, the higher inhibition of **5i** is likely due to the isomerization of the propyl group (Figure 2). Comparing compounds **5f** and **5g** with the most active compound (**5k**), a further decrease in inhibition of the **5g** (IC_50_ = 68.75 ± 1.42 μM) and **5f** (IC_50_ = 136.36 ± 2.57 μM) may be possible due to the replacement of ethyl and methyl groups on one side of the attached moiety, respectively. The lower inhibition activity of **5f** than **5g** could be possibly due to the replacing of ethyl with a methyl group on one side of the attached substituent. Overall, it can be concluded that the propyl group on one side of the substituent, among the ketone series having straight-chain alkanes on one side and benzene ring on the other side, may have a beneficial effect on the interaction with the α-glucosidase enzyme. Compound **5a** and **5b** (having methyl group on one side and electron-withdrawing substituent on the para position of a benzene ring on the other side) was found inactive, indicating that methyl with para hydroxyl and methyl with para nitro benzene groups had no beneficial effect on the interaction with the α-glucosidase enzyme. Similarly, compound **5e** having cyclohexane on one side and benzene on the other side was found to be inactive. The higher inhibition of **5m** (IC_50_ = 11.83 ± 0.18 μM) than **5n** (IC_50_ = 14.24 ± 0.16 μM) might be possibly due to the presence of methoxy group at ortho substation on benzene ring on one side in addition to having the same functional group on the other side. Compounds **5j** (IC_50_ = 29.31 ± 0.60 μM), **5c** (IC_50_ = 35.52 ± 2.04 μM), and **5d** (IC_50_ = 42.03 ± 3.51 μM) displayed promising inhibition in the series as well (Table 1).

### 2.3. Molecular Docking Studies

The mode of binding of all the active compounds was studied in the active site of the *α*-glucosidase enzyme by molecular docking. The binding orientation of the parent compound (**1**) indicates that the 2-fluoro-substituted-biphenyl moiety of the compound lies near the entrance of the active site where Tyr158 provides hydrophobic interaction to the biphenyl ring of the compound **1**, while its hydrazide moiety mediated H-bonding (H-bond) with the side chains of Asp69 and Arg442. However, the substitution of the R group at the terminal amino group of **1** was responsible to tilt the other compounds towards the hydrophobic pocket in the active site constituted by Trp58, Phe301, Phe303, and Tyr347 and the R group resided in that pocket.

Among compounds **4a**–**4p**, **4h** was found most active inhibitor with an IC_50_ value of 1.52 µM, followed by **4d** and **4b** which exhibited an IC_50_ value of 4.77 µM and 7.16 µM, respectively. The binding orientation of compound **4h**, which is the second most active compound in this series, was like the docked conformation of **5k**. The addition of isobutyl-benzene at the R position of compound **4h** made this molecule tilt more towards the Phe303 which stabilized the **4h** by π-π interaction. Due to the tilted position, the hydrazide moiety of **4h** got far away from Glu277 and His351. A similar trend of binding was followed by compounds **4d** and **4b**. The hydrazide moiety of **4d** mediated H-bonding with the side chains of Asp215 and His351, while the hydrazide group of **4b** did not form any significant interaction; however, its nitro group interacted with the side chain of Arg213 through H-bond.

The compounds **4g**, **4i**, **4l**, **4p**, **4j**, **4m**, and **4o** showed moderate inhibitory activities with IC_50_ in the range of 11.42 to 22.72 µM. The docked orientation of **4g** reflects that the substituted bromo–flouro–benzene ring is responsible to decrease the activity of this compound since the bulky group is not favorable for binding at this position. The addition of dibromophenol ring at the R position in **4i** further reduced the inhibitory activity of **4i**. The compound **4i** further slipped towards the entrance of the active site, thereby its carbonyl only interacted with the side chain of Arg213. The docked orientations of compounds **4g**, **4i**, **4j**, **4l**, **4m,** and **4p** are quite similar; however, the hydrazide moiety of **4g**, **4l**, and **4m** interacted with the Glu277 and His351 like compound **4h**, while the hydrazide group of **4p** mediated H-bonding with Asp215 and His351. The hydrazide carbonyl group of **4j** was stabilized by His351, whereas the ethoxy group of **4j** and hydrazide carbonyl of **4o** accepted H-bond with the side chain of Tyr347. One of the −OH groups of **4c** (IC_50_ = 48.39 µM) exhibited H-bonding with Asp352, while its hydrazide group interacted with Asp215 and His351. The compounds **4k**, **4n,** and **4f** exhibited the least activity with IC_50_ values of 146.78, 147.26, and 426.82 µM, respectively. The compound **4k** slipped more towards the outside of the active site while **4n** adopt a bent position, the carbonyl of **4k** was stabilized by the side chain of Arg442 and the hydrazide of **4n** mediated H-bonding with Asp215 and Arg213. The docked orientation of **4f** was twisted and its biphenyl ring acquired a position in opposite direction, due to the reason this compound did not form any significant interaction in the active site, which may be the reason for the least activity in this compound.

**Figure 3:**
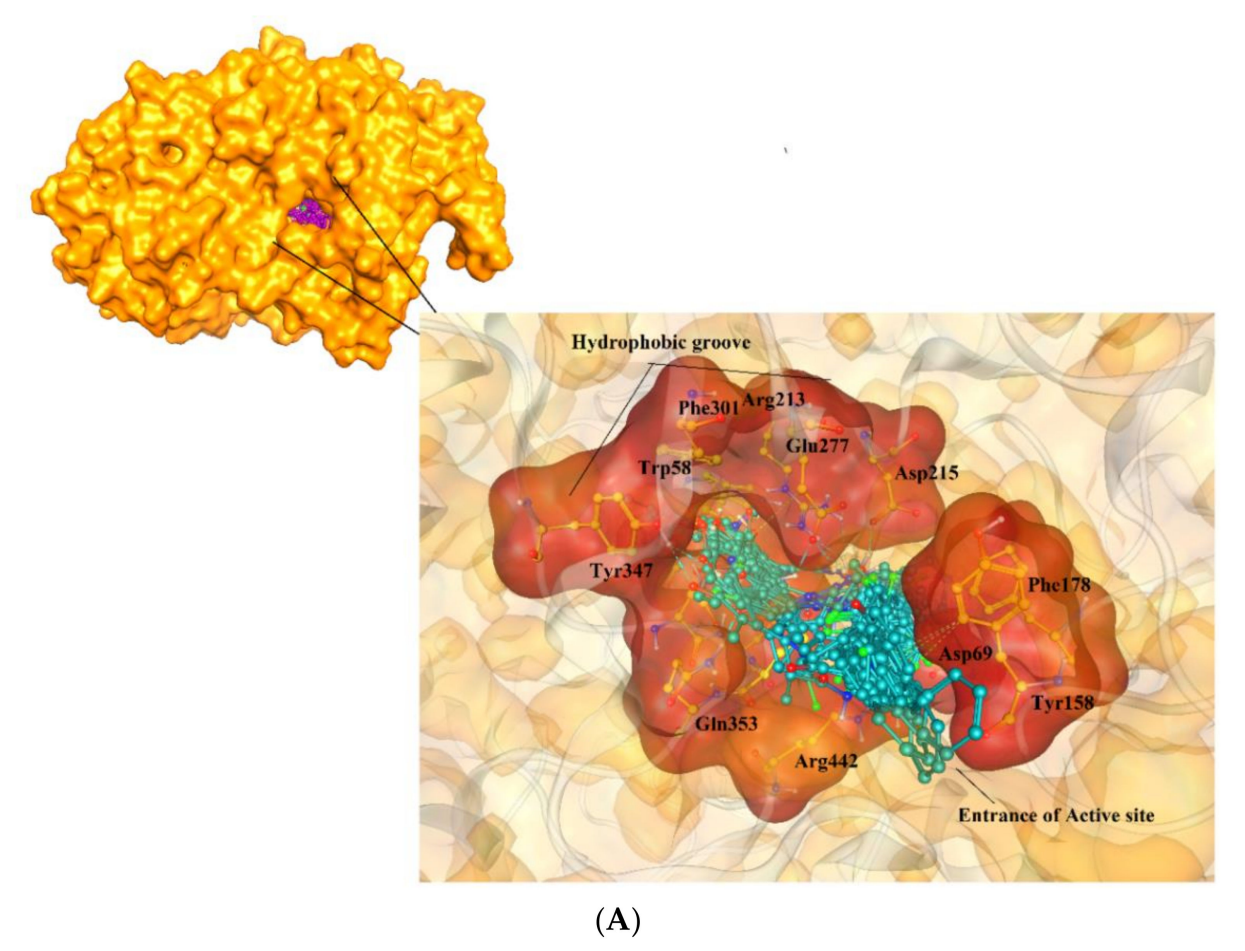

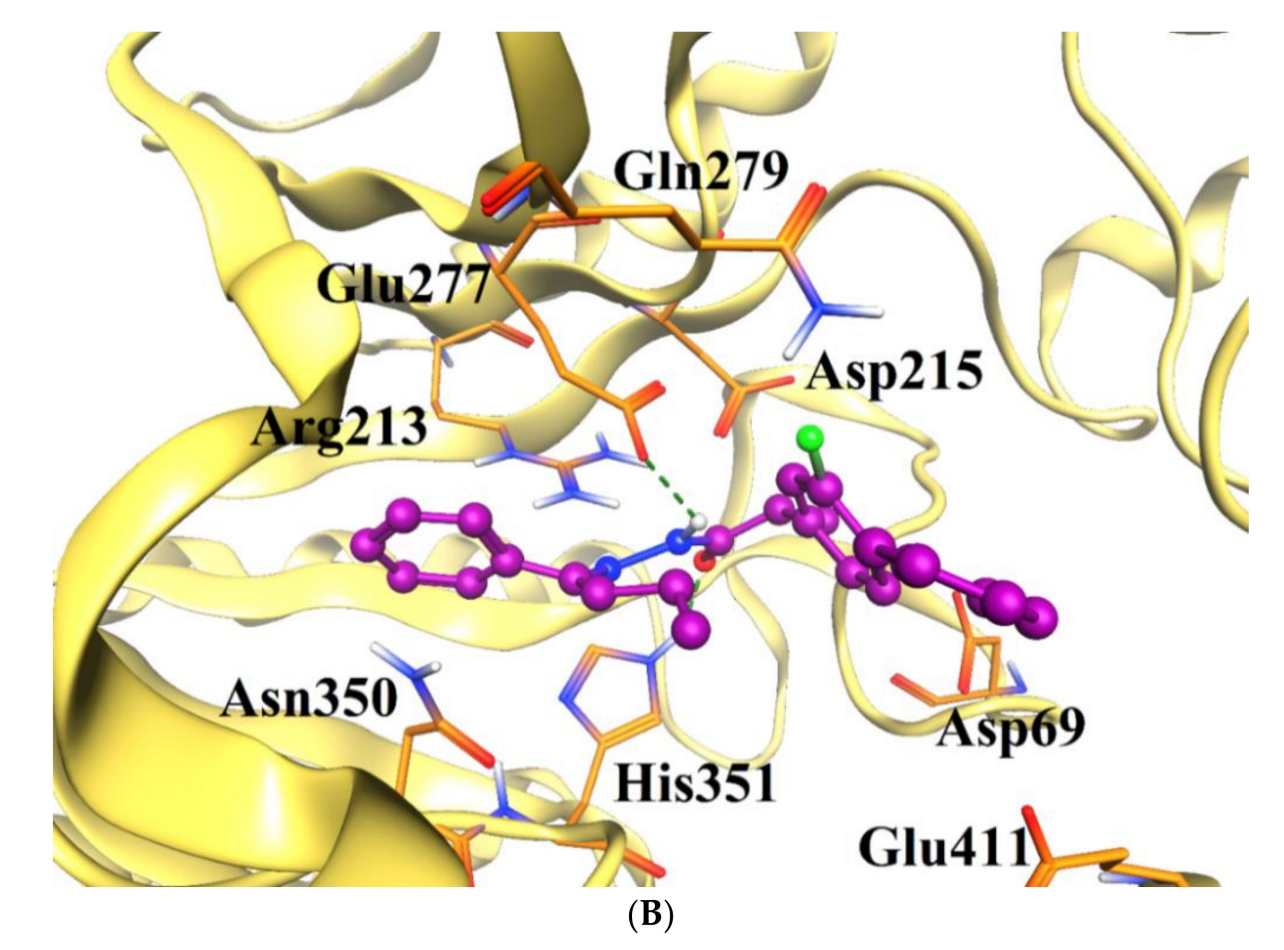
(**A**) The docked orientations of all the active compounds are shown in the active site of α-glucosidase enzyme. The entrance of the active site and the hydrophobic pocket is displayed in the surface model, the compounds are presented in cyan ball and stick model. (**B**) The binding mode of the most active compound (**5k**, shown in magenta ball and stick model) is displayed in the active site of α-glucosidase. The active site residues are presented in orange stick model, and the protein is shown in gold ribbon. The H-bonds are displayed in green dotted line.

In this series, compound **5k** was identified as the most active inhibitor against *α*-glucosidase. Like **4h**, the hydrazide group of **5k** mediated H-bonds with the side chains of Glu277 and His351, and the side chains of Trp58, and Phe301 provide hydrophobic interaction to the R group of **5k**, and the biphenyl ring of the compound formed hydrophobic interaction with the side chain of Phe178 at the entrance of the active site of the enzyme. The docked mode of **5h** was found more twisted, therefore the carbonyl group of **5h** interacted with the side chain of Arg213. The addition of pentyl-benzene moiety at the R position of **5i** made the compound more flexible. Due to high flexibility, **5i** moved more towards the entrance of the active site, however, the hydrazide group of **5i** retained its H-bonding with Glu277 and His351, and π-π interaction with Phe303. The hydrazide moiety of **5l, 5n, 5j, 5c,** and **5f** interacted with the Glu277 and His351-like compounds **5k** and **4h**. The biphenyl ring of **5m** was tilted towards Arg69, His112, and Phe178, and its hydrazide group mediated H-bond with the Asn350 and Glu277. Like **4f**, the biphenyl ring of **5d** was tilted in opposite direction, therefore **5d** could only make hydrophobic interaction with Glu277 and Phe303, as a result, its activity was decreased. Similarly, the biphenyl ring of **5g** slipped more outside the active site, while its R-group acquired the position near the biphenyl ring of other compounds, instead of residing in the hydrophobic groove. Due to this conformational change, the compound showed the least inhibitory activity. The docking results are tabulated in Table 2. In docking studies, it was observed that Glu277 and HIS351 play a crucial role in the stabilization of binding of those compounds in the active site of the enzyme (Figure 3). The docking scores of the compounds are in the range of −7.51 to −3.36 kcal/mol, which indicates that these compounds are good binders of protein, therefore exhibit excellent inhibitory potential for α-glucosidase.

## 3. Experimental Part

### 3.1. General Instrumentation

All the chemicals, solvents, and reagents used in this research work were analytical grade and purchased from Sigma-Aldrich, MO, USA. For thin-layer chromatography (TLC), pre-coated aluminum sheets (silica gel 60F-254, Merck, Darmstadt, Hesse, Germany) were used and visualized with the help of UV light at 254 nm. LC-HRESIMS (liquid chromatography high-resolution electrospray ionization mass spectrometry) spectra were recorded on a mass spectrometer (Waters Quattro Premier XE, Waters, Milford, MA, USA). Melting point apparatus Stuart^®^ SMP10 was used for the determination of melting points of the synthesized derivatives. The ^1^H- and ^13^C NMR spectra were recorded on a nuclear magnetic resonance (NMR) spectrometer (Bruker, Zürich, Switzerland) operating at 600 MHz (150 MHz for ^13^C; 600 MHz for ^1^H; chemical shift (δ) = ppm; coupling constants (*J*) = Hz) using the solvent peaks as internal references (CDCl_3_, δ_H_: 7.26; δ_C_: 77.0). Column chromatography was carried out by using silica gel of the selected particle size of 100–200 mesh.

### 3.2. 2-(2-Fluorobiphenyl-4-yl)propanehydrazide *(**1**)*

Coupling reagent CDI (1,1′-carbonyldiimidazole) was used for the synthesis of hydrazide by reacting an appropriate amount of flurbiprofen with hydrazine hydrate in the presence of triethyl amine in THF solvent. In this reaction, flurbiprofen (0.01 M) and CDI (0.012 M) were taken in a round bottom flask and dissolved in minimum amount of tetrahydrofuran (THF). The reaction mixture was stirred for 30–40 min to activate the carboxylic group of flurbiprofen. Finally, hydrazine hydrates (4 mL) were added and reflux it for 5 h, with continuous stirring. The completion of the reaction was checked through TLC (4:6 = ethyl acetate: hexane) from time to time. After completion the reaction mixture was cooled to room temperature (RT) and poured onto crushed ice (100 mL), precipitates were formed, filtered, and washed with excess distilled water to obtain pure hydrazide for further reactions, and the ppt. were dried, weighed and recrystallize with ethanol earlier reported by Momin khan et al. [15].

### 3.3. General Procedure for the Synthesis of Substituted Schiff’s Bases of Flurbiprofen (4a–p and 5a–n)

Acid hydrazide of flurbiprofen (0.0005 mole) and corresponding substituted aromatic/aliphatic aldehydes and ketones (0.0005 mole) were added to a 100 mL RB flask and dissolved in 15 mL ethanol in the presence of 4–5 drops glacial acetic acid as a catalyst. The reaction mixture was refluxed for 3–4 h with constant stirring. The reaction progress was checked through TLC (3:7 = ethyl acetate:hexane). After completion of reaction, it was poured into ice-cold distilled water, precipitates were formed, filtered, and washed with distilled water and hot hexane to remove unreacted aldehydes/ketones. The ppt. was dried under air, recrystallized with ethanol, and weighed. The desired products were further confirmed by different spectroscopic techniques like HRMS, and NMR (Figures S1–S30, Supplementary Materials).

### 3.4. Spectral Interpretation of the Synthesized Compounds

#### 3.4.1. 2-(2-Fluoro-[1,1′-biphenyl]-4-yl)propanehydrazide (**1**)

Solid; yield: 90%; M.P.: 120–121 °C; ^1^H-NMR (600 MHz, CDCl_3_; ⸹, ppm): 9.12 (s, 1H, NH), 7.42 (m, 7H, H-5, H-6, H-8, H-9, H-10, H-11, H-12), 7.22 (s, 1H, H-2), 4.24 (s, 2H, NH_2_), 3.60 (q, *J* = 7.2 Hz, 1H, -CH-CH_3_), 1.35 (d, *J* = 7.2 Hz, 3H, -CH_3_-CH-), ^13^C-NMR (150 MHz, CDCl_3_ δ (ppm): 172.3 (C-14), 157.8 (C-3), 137.0 (C-1), 134.9 (C-7), 130.1 (C-5), 129.0 (C-9, C-11), 128.0 (C-4, C-8, C-12), 127.5 (C-10), 125.5 (C-6), 118.2 (C-2), 42.7 (C-13), 18.1 (C-15). LC-HRMS (ESI^+^): Found [M + H]^+^: 258.2176; C_15_H_15_FN_2_O.

#### 3.4.2. (E)-2-(2-Fluoro-[1,1′-biphenyl]-4-yl)-N′-(3,4,5-trimethoxybenzylidene)propanehydrazide (**4a**)

Solid; yield: 88%; M.P.: 139–140 °C; ^1^H-NMR (600 MHz, CDCl_3_; ⸹, ppm): 9.68 (s, 1H, -CH=N-), 7.66 (s, 1H, -NH), 7.46 (d, *J* = 7.2 Hz, 2H, Ar-H, H-8, H-12), 7.39 (t, *J* = 7.2 Hz, 2H, Ar-H, H-9, H-11), 7.33 (m, 4H, Ar-H, H-2, H-5, H-6, H-10), 6.85 (s, 2H, Ar-H, H-2′, H-6′), 4.70 (q, *J* = 6.6 Hz, 1H, -CH-CH_3_), 3.91 (s, 6H, 2-OCH_3_), 3.86 (s, 3H, -OCH_3_), 1.57 (d, *J* = 7.2 Hz, 3H, CH_3_-CH-), ^13^C NMR 150 MHz, CDCl_3_ δ (ppm): 175.7 (C-14), 160.4 (C-3), 158.8 (C-3′, C-5′), 153.5 (C-1′′), 143.7 (C-4′), 139.9 (C-1), 135.4 (C-7), 130.7 (C-5), 128.9 (C-9, C-11), 128.4 (C-4, C-8, C-12), 123.5 (C-1′), 115.7 (C-10), 115.5 (C-2), 104.2 (C-2′, C-6′), 60.9 (C-3′′), 56.1 (C-2′′, C-4′′), 41.6 (C-13), 18.2 (C-15). LC-HRMS (ESI^+^): Found [M + H]^+^: 437.1862; C_22_H_25_FN_2_O_4_.

#### 3.4.3. (E)-2-(2-Fluoro-[1,1′-biphenyl]-4-yl)-N′-(3-nitrobenzylidene)propanehydrazide (**4b**)

Solid; yield: 70%; M.P.: 145–147 °C; ^1^H-NMR (600 MHz, CDCl_3_; ⸹, ppm): 9.64 (s, 1H, -CH=N-), 8.52 (s, 1H, -NH), 8.26 (d, *J* = 8.4 Hz, 1H, Ar-H, H-5), 7.95 (d, *J* = 7.8 Hz, 1H, Ar-H, H-4′), 7.83 (s, 1H, H-2′), 7.61 (t, *J* = 7.2 Hz, 1H, Ar-H, H-5′), 7.52 (d, *J* = 7.2 Hz,, 2H, Ar-H, H-8, H-12), 7.41 (m, 2H, H-9, H-11), 7.36 (t, *J* = 7.8 Hz, 1H, Ar-H, H-10), 7.31 (d, *J* = 7.8 Hz, 1H, Ar-H, H-6), 7.30 (s, 1H, Ar-H, H-2), 7.26 (d, *J* = 7.2 Hz, 1H, H-6′), 3.84 (q, *J* = 7.2 Hz, 1H, -CH-CH_3_), 1.66 (d, *J* = 7.2 Hz, 3H, CH_3_-CH-), ^13^C-NMR 150 MHz, CDCl_3_ δ (ppm): 176.0 (C-14), 158.9 (C-3), 148.6 (C-3′), 140.8 (C-1′′), 135.5 (C-1), 135.4 (C-7), 132.5 (C-6′), 130.8 (C-5), 130.8 (C-5′), 128.9 (C-9, C-11), 128.4 (C-8), 127.6 (C-4), 127.6 (C-10), 127.6 (C-1′), 124.4 (C-6), 123.9 (C-4′), 121.6 (C-2′), 115.4 (C-2), 41.6 (C-13), 18.3 (C-15). LC-HRMS (ESI^+^): Found [M + H]^+^: 392.1387; C_22_H_18_FN_3_O_3_.

#### 3.4.4. (E)-N′-(2,4-Dihydroxybenzylidene)-2-(2-fluoro-[1,1′-biphenyl]-4-yl)propanehydrazide (**4c**)

Solid; yield: 67%; M.P.: 133–135 °C; ^1^H-NMR (600 MHz, CDCl_3_; ⸹, ppm): 10.16 (s, 1H, -CH=N-), 8.84 (s, 1H, -OH), 8.14 (s, 1H, -OH), 7.77 (s, 1H, -NH), 7.53 (m, 6H, Ar-H, H-5, H-6, H-9, H-10, H-11, H-6′), 7.05 (d, *J* = 7.8 Hz, 1H, Ar-H, H-5′), 6.47 (s, 1H, H-3′), 6.43 (d, *J* = 8.4 Hz, 2H, Ar-H, H-8, H-12), 6.33 (s, 1H, Ar-H, H-2), 4.38 (q, *J* = 6.6 Hz, 1H, -CH-CH_3_), 1.69 (d, *J* = 7.2 Hz, 3H, CH_3_-CH-), ^13^C-NMR 150 MHz, CDCl_3_ δ (ppm): 173.7 (C-14), 169.1 (C-2′), 160.7 (C-4′), 158.6 (C-3), 154.8 (C-1′′), 150.3 (C-1), 147.5 (C-7), 142.8 (C-6′), 135.3 (C-5), 130.6 (C-9, C-12), 128.7 (C-4, C-8, C-12), 127.4 (C-10), 123.6 (C-6), 115.2 (C-2), 110.2 (C-1′), 107.6 (C-5′), 103.3 (C-3′), 29.4 (C-13), 18.8 (C-15). LC-HRMS (ESI^+^): Found [M + H]^+^: 379.1430; C_22_H_19_FN_3_O_3_.

#### 3.4.5. (E)-2-(2-Fluoro-[1,1′-biphenyl]-4-yl)-N′-(2-hydroxy-3-methoxybenzylidene)propanehydrazide (**4d**)

Solid; yield: 80%; M.P.: 154–155 °C; ^1^H-NMR (600 MHz, CDCl_3_; ⸹, ppm): 9.94 (s, 1H, -CH=N-), 9.81 (s, 1H, -NH), 7.98 (s, 1H, Ar-H, H-2), 7.54 (m, 3H, Ar-H, H-6, H-8, H-12), 7.28 (m, 3H, Ar-H, H-9, H-10, H-11), 7.03 (m, 3H, Ar-H, H-4′, H-5′, H-6′), 4.49 (q, *J* = 6.6 Hz, 1H, -CH-CH_3_), 3.95 (d, *J* = 7.2 Hz, 3H, CH_3_-CH-), 1.69 (s, 3H, -OCH_3_), ^13^C-NMR 150 MHz, CDCl_3_ δ (ppm): 196.6 (C-14), 160.6 (C-3), 151.6 (C-2′), 148.0 (C-3′), 147.1 (C-1′′), 141.6 (C-1), 135.4 (C-7), 131.0 (C-5), 128.9 (C-9, C-11), 128.3 (C-4, C-8, C-12), 127.5 (C-10), 124.5 (C-6), 123.9 (C-6′), 122.2 (C-1′), 119.6 (C-2), 117.9 (C-5′), 115.2 (C-4′), 56.2 (C-2′′), 41.6 (C-13), 18.8 (C-15). LC-HRMS (ESI^+^): Found [M + H]^+^: 393.1653; C_23_H_21_FN_3_O_3_.

#### 3.4.6. (E)-N′-(4-(Diethylamino)benzylidene)-2-(2-fluoro-[1,1′-biphenyl]-4-yl)propanehydrazide (**4e**)

Solid; yield: 84%; M.P.: 144–145 °C; ^1^H-NMR (600 MHz, CDCl_3_; ⸹, ppm): 8.81 (s, 1H, -NH), 7.68 (s, 1H, -CH=N-), 7.57 (s, 1H, Ar-H, H-2), 7.49 (m, 4H, Ar-H, H-8, H-9, H-11, H-12), 7.43 (t, *J* = 7.8 Hz, 1H, Ar-H, H-10), 7.35 (m, 2H, Ar-H, H-5, H-6), 7.31 (d, *J* = 7.2 Hz, 2H, Ar-H, H-2′, H-6′), 6.66 (d, *J* = 8.4 Hz, 2H, Ar-H, H-3′, H-5′), 4.75 (q, *J* = 6.6 Hz, 1H, -CH-CH_3_), 4.22 (m, 4H, 2 x -CH_2_-CH_3_), 1.67 (d, *J* = 6.6 Hz, CH_3_-CH-), 1.18 (m, 6H, 2 x CH_3_-CH_2_), ^13^C-NMR 150 MHz, CDCl_3_ δ (ppm): 190.0 (C-14), 175.1 (C-3), 167.7 (C-4′), 160.4 (C-1′′), 158.8 (C-1), 143.0 (C-7), 135.7 (C-5), 128.9 (C-9, C-11), 128.3 (C-2′, C-6′), 128.4 (C-4, C-8, C-12), 127.4 (C-3′, C-5′), 123.9 (C-10), 115.7 (C-6), 111.2 (C-1′), 110.5 (C-2), 68.1 (C-2′′, C-4′′), 44.6 (C-13), 18.1 (C-15), 10.9 (C-3′, C-5′′). LC-HRMS (ESI^+^): Found [M + H]^+^: 418.2281; C_26_H_28_FN_3_O.

#### 3.4.7. (E)-2-(2-Fluoro-[1, 1′-biphenyl]-4-yl)-N′-(3-hydroxy-4-methoxybenzylidene)propanehydrazide (**4f**)

Solid; yield: 74%; M.P.: 126–127 °C; ^1^H-NMR (600 MHz, CDCl_3_; ⸹, ppm): 9.42 (s, 1H, -NH), 7.63 (s, 1H, -CH=N-), 7.49 (d, *J* = 7.2 Hz, 2H, Ar-H, H-8, H-12), 7.39 (t, *J* = 9.0 Hz, 3H, Ar-H, H-9, H-10, H-11), 7.32 (m, 2H, Ar-H, H-5, H-6), 7.27 (d, *J* = 8.4 Hz, 1H, Ar-H, H-5′), 6.84 (d, *J* = 8.4 Hz, 1H, H-6′), 5.73 (s, 1H, -OH), 4.76 (q, *J* = 7.2 Hz, 1H, -CH-CH_3_), 3.90 (s, 3H, -OCH_3_), 1.57 (d, *J* = 6.6 Hz, 3H, CH_3_-CH-), ^13^C-NMR 150 MHz, CDCl_3_ δ (ppm): 175.8 (C-14), 160.4 (C-3), 158.8 (C-4′), 148.5 (C-3′), 146.0 (C-1′′), 143.7 (C-1), 142.7 (C-7), 135.6 (C-1′), 130.6 (C-9, C-11), 128.9 (C-4, C-8, C-12), 127.4 (C-10), 124.0 (C-5), 120.8 (C-6′), 115.6 (C-2), 111.9 (C-2′), 110.4 (C-5′), 56.0 (C-2′′), 41.0 (C-13), 18.1 (C-15). LC-HRMS (ESI^+^): Found [M + H]^+^: 393.1625; C_23_H_21_FN_2_O_3_.

#### 3.4.8. (E)-N′-(4-Bromo-2-fluorobenzylidene)-2-(2-fluoro-[1,1′-biphenyl]-4-yl)propanehydrazide (**4g**)

Solid; yield: 85%; M.P.: 166–168 °C; ^1^H-NMR (600 MHz, CDCl_3_; ⸹, ppm): 9.34 (s, 1H, -NH), 8.25 (s, 1H, -CH=N-), 7.89 (s, 1H, Ar-H, H-3′), 7.73 (t, *J* = 7.8 Hz, 1H, Ar-H, H-10), 7.51 (d, *J* = 7.2 Hz, 2H, Ar-H, H-8, H-12), 7.42 (t, *J* = 7.8 Hz, 2H, Ar-H, H-5, H-6), 7.28 (d, *J* = 9.6 Hz, 1H, Ar-H, H-5′), 7.21 (m, 2H, Ar-H, H-2, H-6′), 4.69 (q, *J* = 6.6 Hz, 1H, -CH-CH_3_), 1.57 (d, *J* = 6.6 Hz, 3H, CH_3_-CH-), ^13^C-NMR 150 MHz, CDCl_3_ δ (ppm): 175.6 (C-14), 161.7 (2′), 160.5 (C-3), 160.0 (1′′), 158.8 (C-1), 142.4 (C-7), 136.1 (C-6′), 135.5 (C-5), 128.9 (C-9, C-11), 128.4 (C-4, C-8, C-12), 127.6 (C-10), 127.5 (C-4′), 123.7 (C-5′), 119.7 (C-6), 119.6 (C-2′), 115.6 (C-1′), 41.4 (C-13), 18.2 (C-15). LC-HRMS (ESI^+^): Found [M + H]^+^: 445.0526; C_22_H_17_BrF_2_N_2_O

#### 3.4.9. (E)-N′-(3-Bromobenzylidene)-2-(2-fluoro-[1,1′-biphenyl]-4-yl)propanehydrazide (**4h**)

Solid; yield: 70%; M.P.: 147–148 °C; ^1^H-NMR (600 MHz, CDCl_3_; ⸹, ppm): 9.04 (s, 1H, -NH), 7.78 (s, 1H, -CH=N-), 7.63 (s, 1H, Ar-H, H-2′), 7.51 (m, 5H, Ar-H, H-4′, H-5, H-6, H-8, H-12), 7.40 (t, *J* = 7.2 Hz, 3H, Ar-H, H-9, H-10, H-11), 7.33 (t, *J* = 7.2 Hz, 3H, 1H, Ar-H, H-5′), 7.27 (d, *J* = 7.8 Hz, 1H, Ar-H, H-6′), 7.20 (s, 1H, Ar-H, H-2), 4.71 (q, *J* = 6.6 Hz, 1H, -CH-CH_3_), 1.57 (d, *J* = 6.6 Hz, 3H, CH_3_-CH-), ^13^C-NMR 150 MHz, CDCl_3_ δ (ppm): 190.7 (C-14), 175.6 (C-3), 160.5 (C-1′′), 158.8 (C-1), 141.7 (C-7), 137.3 (C-4′), 135.6 (C-2′) 133.0 (C-5), 132.3 (C-5′), 130.7 (C-3′), 130.3 (C-6′), 129.7 (C-4), 128.9 (C-9, C-11), 128.3 (C-8, C-12), 127.5 (C-6), 125.8 (C-2), 123.8 (C-10), 123.0 (C-1′), 41.5 (C-13), 18.2 (C-15). LC-HRMS (ESI^+^): Found [M + H]^+^: 425.0662; C_22_H_18_BrFN_2_O.

#### 3.4.10. (E)-N′-(3,5-Dibromo-4-hydroxybenzylidene)-2-(2-fluoro-[1,1′-biphenyl]-4- yl)propane hydrazide (**4i**)

Solid; yield: 95%; M.P.: 161–162 °C; ^1^H-NMR (600 MHz, CDCl_3_; ⸹, ppm): 9.19 (s, 1H, -NH), 7.98 (s, 1H, -CH=N-), 7.78 (s, 1H, -OH), 7.69 (s, 2H, Ar-H, H-2′, H-6′), 7.54 (s, 1H, Ar-H, H-2), 7.49 (d, *J* = 7.8 Hz, 2H, H-8, H-12), 7.40 (m, 4H, Ar-H, H-5, H-6, H-9, H-11), 7.33 (t, *J* = 7.2 Hz, 1H, Ar-H, H-10), 4.67 (q, *J* = 7.2 Hz, 1H, -CH-CH_3_), 1.56 (d, *J* = 7.2 Hz, 3H, CH_3_-CH-), ^13^C-NMR 150 MHz, CDCl_3_ δ (ppm): 188.0 (C-14), 175.6 (C-3), 160.5 (C-4′), 158.8 (C-1′′), 135.5 (C-1) 133.6 (C-7), 132.3 (C-2′, C-6′), 128.9 (C-4, C-8, C-12), 128.4 (C-9, C-11), 127.5 (C-3′, C-5′), 123.8 (C-1′), 123.0 (C-5), 110.7 (C-10), 41.5 (C-13), 18.2 (C-15). HR-EIMS (ESI^+^): Found [M + H]^+^: 520.9690; C_22_H_17_Br_2_FN_2_O_2_.

#### 3.4.11. (E)-N′-(3-Ethoxy-4-hydroxybenzylidene)-2-(2-fluoro-[1,1′-biphenyl]-4-yl)propanehydrazide (**4j**)

Solid; yield: 90%; M.P.: 173–174 °C; ^1^H-NMR (600 MHz, MeOD ; ⸹, ppm): 8.01 (s, 1H, -NH), 7.60 (s, 1H, -CH=N-), 7.53 (d, *J* = 7.8 Hz, 2H, Ar-H, H-8, H-12), 7.45 (t, *J* = 8.4 Hz, 3H, Ar-H, H-9, H-10, H-11), 7.38 (d, *J* = 7.8 Hz, 1H, Ar-H, H-5), 7.31 (m, 2H, Ar-H, H-5′, H-6′), 7.05 (d, *J* = 7.8 Hz, 1H, Ar-H, H-6), 6.84 (m, 1H, Ar-H, H-2′) 4.22 (m, 2H, -CH_2_-CH_3_), 3.79 (q, *J* = 6.6 Hz, 1H, -CH-CH_3_), 1.58 (d, *J* = 7.2 Hz, 3H, CH_3_-CH-), 1.47 (m, 3H, CH_3_-CH_2_-) ^13^C-NMR 150 MHz, MeOD δ (ppm): 177.2 (C-14), 172.7 (C-3), 161.8 (C-4′), 160.1 (C-3′), 150.8 (C-1′′), 150.3 (C-1), 148.7 (C-7), 146.0 (C-1′), 144.3 (C-5) 136.8 (C-10), 131.9 (C-6), 129.9 (C-9, C-11), 129.4 (C-8, C-12), 128.7 (C-4), 127.2 (C-6′), 124.8 (C-5′), 124.3 (C-2′), 123.2 (C-2), 65.4 (C-2′′), 49.1 (C-13), 19.0 (C-15), 15.0 (C-3′′). LC-HRMS (ESI^+^): Found [M + H]^+^: 407.1765; C_24_H_23_FN_2_O_3_.

#### 3.4.12. (E)-N′-Butylidene-2-(2-fluoro-[1,1′-biphenyl]-4-yl)propanehydrazide (**4k**)

Solid; yield: 87%; M.P.: 168–169 °C; ^1^H-NMR (600 MHz, MeOD ; ⸹, ppm): 7.53 (t, *J* = 7.8 Hz, 2H, Ar-H, H-9, H-11), 7.43 (d, *J* = 7.2 Hz, 2H, Ar-H, H-8, H-12), 7.40 (d, *J* = 7.8 Hz, 1H, Ar-H, H-5), 7.38 (t, *J* = 7.2 Hz, 1H, Ar-H, H-10), 7.28 (d, *J* = 7.8 Hz, 1H, Ar-H, H-6), 7.23 (s, 1H, Ar-H, H-2), 3.70 (q, *J* = 6.6 Hz, 1H, -CH-CH_3_), 2.21 (m, 2H, H-2′), 1.57 (d, *J* = 7.2 Hz, 3H, CH_3_-CH-), 1.42 (m, 2H, H-3′), 0.95 (t, *J* = 7.8 Hz, 3H, H-4′), ^13^C-NMR 150 MHz, MeOD δ (ppm): 177.2 (C-14), 172.4 (C-3), 161.8 (C-1′), 160.1 (C-1), 156.2 (C-7), 151.3 (C-5), 151.1 (C-10), 144.2 (C-6′), 136.8 (C-2) 131.9 (C-4), 129.9 (C-9, C-11), 129.4 (C-8, C-12), 128.7 (C-10), 45.5 (C-13), 38.1 (C-2′), 21.9 (C-3′), 19.5 (C-15), 12.2 (C-4′). LC-HRMS (ESI^+^): Found [M + H]^+^: 313.1713; C_19_H_21_FN_2_O.

#### 3.4.13. (E)-2-(2-Fluoro-[1, 1′-biphenyl]-4-yl)-N′-hexylidenepropanehydrazide (**4l**)

Solid; yield: 76%; M.P.: 129–130 °C; ^1^H-NMR (600 MHz, CDCl_3_; ⸹, ppm): 8.90 (s, 1H, -NH), 7.50 (d, *J* = 7.2 Hz, 2H, Ar-H, H-8, H-12), 7.41 (t, *J* = 7.2 Hz, 2H, Ar-H, H-9, H-11), 7.34 (m, 2H, Ar-H, H-5, H-6), 7.23 (s, 1H, Ar-H, H-2), 7.11 (m, 1H, Ar-H, H-10), 4.62 (q, *J* = 7.2 Hz, 1H, CH-CH_3_), 2.23 (t, *J* = 7.2 Hz, 1H, -CH=N-), 1.58 (m, 4H, H-2′, H-3′), 1.29 (m, 4H, H-4′, H-5′), 0.88 (m, 3H, H-6′), ^13^C-NMR 150 MHz, CDCl_3_ δ (ppm): 175.1 (C-14), 160.2 (C-3), 158.5 (C-1′), 147.5 (C-1), 142.6 (C-7), 135.5 (C-5), 128.7 (C-9, C-11), 128.3 (C-4, C-8, C-12), 127.3 (C-10), 123.7 (C-6), 115.6 (C-2), 40.9 (C-13), 32.0 (C-3′), 31.2 (C-4′), 25.8 (C-2′), 22.4 (C-5′), 18.0 (C-15), 13.9 (C-6′). LC-HRMS (ESI^+^): Found [M + H]^+^: 341.2017; C_21_H_25_FN_2_O.

#### 3.4.14. (E)-2-(2-Fluoro-[1,1′-biphenyl]-4-yl)-N′-(2-methoxybenzylidene)propanehydrazide (**4m**)

Solid; yield: 69%; M.P.: 131–132 °C; ^1^H-NMR (600 MHz, CDCl_3_; ⸹, ppm): 10.45 (s, 1H, -NH), 9.05 (s, 1H, -CH=N-), 8.12 (s, 1H, Ar-H, H-2), 7.87 (d, *J* = 7.8 Hz, 1H, Ar-H, H-5), 7.81 (d, *J* = 7.2 Hz, 3H, Ar-H, H-6, H-8, H-12), 7.54 (t, *J* = 8.4 Hz, 3H, Ar-H, H-4′, H-5′, H-10), 7.49 (d, *J* = 7.8 Hz, 2H, Ar-H, H-3′, H-6′), 7.39 (t, *J* = 7.8 Hz, 2H, Ar-H, H-9, H-11), 4.76 (q, *J* = 7.2 Hz, 1H, -CH-CH_3_), 3.82 (s, 3H, -OCH_3_), 1.57 (d, *J* = 7.2 Hz, 3H, CH_3_-CH-), ^13^C-NMR 150 MHz, CDCl_3_ δ (ppm): 189.7 (C-14), 175.3 (C-3), 161.6 (C-2′), 157.8 (C-1′′), 139.7 (C-1), 135.8 (C-7), 131.3 (C-4′), 130.4 (C-6′), 128.7 (C-9, C-11), 128.4 (C-4, C-8, C-12), 127.3 (C-5), 125.9 (C-10), 124.6 (C-6), 123.7 (C-5′), 121.9 (C-1′), 55.6 (C-2′′), 41.2 (C-13), 18.2 (C-15). LC-HRMS (ESI^+^): Found [M + H]^+^: 377.1641; C_23_H_21_FN_2_O_2_.

#### 3.4.15. (E)-2-(2-Fluoro-[1,1′-biphenyl]-4-yl)-N′-(naphthalen-1-ylmethylene)propanehydrazide (**4n**)

Solid; yield: 92%; M.P.: 168–170 °C; ^1^H-NMR (600 MHz, CDCl_3_; ⸹, ppm): 10.39 (s, 1H, -NH), 8.60 (d, *J* = 8.4 Hz, 1H, Ar-H, H-5), 8.34 (s, 1H, -CH=N-), 8.10 (d, *J* = 8.4 Hz, 1H, Ar-H, H-6), 7.79 (d, *J* = 7.2 Hz, 1H, Ar-H, H-3′), 7.69 (t, *J* = 7.8 Hz, 1H, Ar-H, H-10), 7.59 (t, *J* = 7.2 Hz, 3H, Ar-H, H-4′, H-5′, H-9′), 7.53 (m, 2H, Ar-H, H-9, H-11), 7.35 (m, 2H, Ar-H, H-8, H-12), 3.47 (q, *J* = 7.2 Hz, 1H, -CH-CH_3_), 1.62 (d, *J* = 6.6 Hz, 3H, CH_3_-CH-), ^13^C-NMR 150 MHz, CDCl_3_ δ (ppm): 175.5 (C-14), 160.5 C-3), 158.9 (C-1′′), 143.8 (C-1), 136.6 (C-7), 135.3 (C-7′), 133.8 (C-8′), 131.4 (C-5), 130.7 (C-2′), 129.0 (C-1′), 128.9 (C-9, C-11), 128.4 (C-4, C-8, C-12), 128.1 (C-4′, C-5′), 127.5 (C-10), 126.9 (C-10′), 125.2 (C-9′), 124.8 (C-6), 123.8 (C-3′), 115.6 (C-2), 41.6 (C-13), 18.6 (C-15). LC-HRMS (ESI^+^): Found [M + H]^+^: 397.1704; C_26_H_21_FN_2_O.

#### 3.4.16. (E)-2-(2-Fluoro-[1,1′-biphenyl]-4-yl)-N′-octylidenepropanehydrazide (**4o**)

Solid; yield: 62%; M.P.: 132–134 °C; ^1^H-NMR (600 MHz, CDCl_3_; ⸹, ppm): 9.30 (s, 1H, -NH), 7.50 (d, *J* = 7.2 Hz, 2H, Ar-H, H-8, H-12), 7.47 (m, 2H, Ar-H, H-5, H-6), 7.39 (t, *J* = 7.8 Hz, 1H, Ar-H, H-10), 7.21 (m, 1H, Ar-H, H-2), 4.63 (q, *J* = 6.6 Hz, 1H, CH-CH_3_), 2.32 (d, *J* = 7.2 Hz, 3H, CH_3_-CH), 1.61 (m, 2H, H-3′), 1.26 (m, 4H, H-2′, H-4′), 1.21 (m, 9H, H-5′, H-6′, H-7′, H-8′), ^13^C-NMR 150 MHz, CDCl_3_ δ (ppm): 178.2 (C-14), 160.4 (C-3), 158.7 (C-1′), 148.1 (C-1), 142.7 (C-7), 135.6 (C-5), 128.9 (C-9, C-11), 128.4 (C-4, C-8, C-12), 127.5 (C-10), 123.9 (C-6), 115.8 (C-2), 40.8 (C-13), 32.4 (C-6′), 31.7 (C-4′), 29.5 (C-5′), 28.9 (C-2′), 24.8 (C-3′), 22.6 (C-7′), 17.9 (C-15), 14.0 (C-8′). LC-HRMS (ESI^+^): Found [M + H]^+^: 369.2315; C_23_H_29_FN_2_O.

#### 3.4.17. (E)-4-((2-(2-(2-Fluoro-[1,1′-biphenyl]-4-yl)propanoyl)hydrazono)methyl)benzoic acid (**4p**)

Solid; yield: 90%; M.P.: 176–177 °C; ^1^H-NMR (600 MHz, MeOD; ⸹, ppm): 8.20 (s, 1H, -NH), 8.09 (t, *J* = 7.8 Hz, 2H, Ar-H, H-9, H-11), 7.97 (s, 1H, -CH=N-), 7.89 (d, *J* = 7.8 Hz, 1H, Ar-H, H-5), 7.81 (d, *J* = 7.8 Hz, 1H, Ar-H, H-6), 7.54 (d, *J* = 7.2 Hz, 2H, Ar-H, H-8, H-12), 7.46 (m, 4H, Ar-H, H-2′, H-3′, H-5′, H-6′), 7.38 (t, *J* = 7.2 Hz, 1H, Ar-H, H-10), 7.27 (s, 1H, Ar-H, H-2), 3.83 (q, *J* = 6.6 Hz, 1H, CH-CH_3_), 1.60 (d, *J* = 6.6 Hz, 3H, CH_3_-CH), 1.61-1.26 (m, 16H, -CH=N, H-1′, H-2′, H-3′, H-4′, H-5′, H-6′, H-7′), ^13^C-NMR 150 MHz, MeOD δ (ppm): 177.7 (C-14), 173.1 (C-2′′), 148.4 (C-3), 144.3 (C-1′′), 139.9 (C-1′), 132.0 (C-1), 131.0 (C-7), 129.9 (C-3′, C-5′), 129.5 (C-9, C-11), 128.7 (C-2′, C-6′), 127.9 (C-4′), 125.1 (C-5), 124.7 (C-6), 116.3 (C-10), 115.9 (C-2), 57.4 (C-13), 42.5 (C-15). LC-HRMS (ESI^+^): Found [M + H]^+^: 391.1461; C_23_H_19_FN_2_O_3_. 

#### 3.4.18. (E)-2-(2-Fluoro-[1,1′-biphenyl]-4-yl)-N′-(1-(4-hydroxyphenyl)ethylidene)propanehydrazide (**5a**)

Solid; yield: 83%; M.P.: 152 °C; ^1^H-NMR (600 MHz, CDCl_3_; ⸹, ppm): 7.88 (d, *J* = 7.8 Hz, 2H, Ar-H, H-2′, H-6′), 7.60 (d, *J* = 7.8 Hz, 2H, Ar-H, H-3′, H-6′), 7.49 (d, *J* = 7.8 Hz, 2H, Ar-H, H-8, H-12), 7.42 (m, 3H, Ar-H, H-2, H-5, H-6), 6.86 (t, *J* = 7.2 Hz, 3H, Ar-H, H-9, H-10, H-11), 4.78 (q, *J* = 6.6 Hz, 1H, -CH-CH_3_), 2.53 (s, 3H, -CH_3_), 1.56 (d, *J* = 6.6 Hz, 3H, CH_3_-CH-), ^13^C-NMR 150 MHz, CDCl_3_ δ (ppm): 175.8 (C-14), 160.0 (C-4′), 156.9 (C-3), 143.5 (C-1′′), 130.9 (C-1), 130.6 (C-7), 130.4 (C-2′, C-6′), 128.9 (C-9, C-11), 128.4 (C-4, C-8, C-12), 127.8 (C-3′, C-5′), 127.5 (C-10), 115.7 (C-5), 115.5 (C-2), 41.3 (C-13), 18.3 (C-2′′), 12.6 (C-15). LC-HRMS (ESI^+^): Found [M + H]^+^: 377.1657; C_23_H_21_FN_2_O_2_.

#### 3.4.19. (E)-2-(2-Fluoro-[1,1′-biphenyl]-4-yl)-N′-(1-(4-nitrophenyl)ethylidene)propanehydrazide (**5b**)

Solid; yield: 75%; M.P.: 175 °C; ^1^H-NMR (600 MHz, CDCl_3_; ⸹, ppm): 8.75 (s, 1H, -NH), 8.30 (d, *J* = 7.8 Hz, 1H, Ar-H, H-5), 8.24 (d, *J* = 8.4 Hz, 2H, Ar-H, H-3′, H-5′), 8.10 (d, *J* = 7.8 Hz, 1H, Ar-H, H-6), 7.84 (d, *J* = 8.4 Hz, 2H, Ar-H, H-2′, H-6′), 7.49 (d, *J* = 7.8 Hz, 2H, Ar-H, H-8, H-12), 7.41 (t, *J* = 7.8 Hz, 2H, Ar-H, H-9, H-11), 7.21 (m, 2H, Ar-H, H-2, H-10), 4.73 (q, *J* = 6.6 Hz, 1H, -CH-CH_3_), 2.22 (s, 3H, -CH_3_), 1.58 (d, *J* = 6.6 Hz, 3H, CH_3_-CH-), ^13^C-NMR 150 MHz, CDCl_3_ δ (ppm): 175.8 (C-14), 148.1 (C-3), 144.7 (C-4′), 143.7 (C-1′′), 135.4 (C-1′), 130.8 (C-1), 129.3 (C-7), 128.9 (C-9, C-11), 128.4 (C-4, C-8, C-12), 127.6 (C-10), 126.8 (C-2′, C-6′), 123.7 (C-3′, C-5′), 123.7 (C-6), 115.5 (C-2), 41.7 (C-13), 18.5 (C-2′′), 12.7 (C-15). LC-HRMS (ESI^+^): Found [M + H]^+^: 406.1557; C_23_H_20_FN_3_O_3_.

#### 3.4.20. (E)-2-(2-Fluoro-[1,1′-biphenyl]-4-yl)-N′-(6-methoxy-3,4-dihydronaphthalen-1(2H) -ylidene)propanehydrazide (**5c**)

Solid; yield: 73%; M.P.: 187 °C; ^1^H-NMR (600 MHz, CDCl_3_; ⸹, ppm): 9.10 (s, 1H, -NH), 8.00 (d, *J* = 9.0 Hz, 1H, Ar-H, H-5), 7.49 (d, *J* = 7.2 Hz, 2H, Ar-H, H-8, H-12), 7.40 (t, *J* = 7.2 Hz, 2H, Ar-H, H-9, H-11), 7.34 (m, 2H, Ar-H, H-3′, H-4′), 7.25 (t, *J* = 7.8 Hz, 1H, Ar-H, H-10), 6.82 (d, *J* = 9.0 Hz, 1H, Ar-H, H-6), 6.62 (s, 1H, Ar-H, H-2), 3.81 (s, 3H, -OCH_3_), 2.72 (t, *J* = 6.0 Hz, 2H, H-8′), 2.49 (q, *J* = 6.6 Hz, 1H, -CH-CH_3_), 1.91 (m, 4H, H-9′, H-10′), 1.56 (d, *J* = 7.2 Hz, 3H, CH_3_-CH), ^13^C-NMR 150 MHz, CDCl_3_ δ (ppm): 176.4 (C-14), 175.3 (C-5′), 160.5 (C-3), 148.1 (C-1′), 142.9 (C-1), 142.8 (C-7), 141.7 (C-5), 130.6 (C-9, C-11), 128.9 (C-4, C-8, C-12), 127.5 (C-10), 126.4 (C-6), 125.0 (C-7′), 123.9 (C-2′), 115.6 (C-4′, C-6′), 113.2 (C-3′), 112.6 (C-2), 55.3 (C-11′), 41.0 (C-13), 29.6 (C-8′), 24.9 (C-9′), 21.5 (C-10′), 18.2 (C-15). LC-HRMS (ESI^+^): Found [M + H]^+^: 417.1989; C_26_H_25_FN_2_O_2_.

#### 3.4.21. N′-((1Z, 2Z)-1,3-Diphenylallylidene)-2-(2-fluoro-[1,1′-biphenyl]-4-yl)propanehydrazide (**5d**)

Solid; yield: 76%; M.P.: 189 °C; ^1^H-NMR (600 MHz, CDCl_3_; ⸹, ppm): 8.24 (s, 1H, -NH), 8.01 (d, *J* = 7.2 Hz, 1H, Ar-H, H-5), 7.81 (s, 1H, Ar-H, H-2), 7.63 (d, *J* = 7.2 Hz, 1H, Ar-H, H-6), 7.35 (t, *J* = 7.2 Hz, 6H, Ar-H, H-3′, H-4′, H-5′, H-9′, H-10′, H-11′), 7.28 (t, *J* = 6.6 Hz, 3H, Ar-H, H-9, H-10, H-11), 7.19 (d, *J* = 7.8 Hz, 2H, Ar-H, H-2′, H-6′), 7.11 (m, 2H, Ar-H, H-8′, H-12′), 4.82 (q, *J =* 7.2 Hz, 1H, -CH-CH_3_), 1.54 (m, 4H, -CH_2_-CH_2_-). ^13^C-NMR 150 MHz, CDCl_3_ δ (ppm): 175.0 (C-14), 160.8 (C-3), 158.8 (C-1′′), 144.8 (C-3′′), 138.2 (C-1), 137.4 (C-7), 136.0 (C-1), 135.7 (C-7′), 132.7 (C-6′, C-10′), 130.6 (C-9′, C-11′), 129.9 (C-4′, C-4, C-8, C-12), 128.9 (C-8′, C-12′, C-9, C-11), 127.9 (C-2′, C-6′), 123.5 (C-10), 120.8 (C-5), 115.8 (C-2), 40.8 (C-13), 18.2 (C-15). LC-HRMS (ESI^+^): Found [M + H]^+^: 449.2042; C_30_H_25_FN_2_O.

#### 3.4.22. (E)-N′-(Cyclohexyl(phenyl)methylene)-2-(2-fluoro-[1,1′-biphenyl]-4-yl)propanehydrazide (**5e**)

Solid; yield: 90%; M.P.: 167 °C; ^1^H-NMR (600 MHz, CDCl_3_; ⸹, ppm): 8.14 (s, 1H, -NH), 7.93 (d, *J* = 7.8 Hz, 1H, Ar-H, H-5), 7.54 (d, *J* = 7.8 Hz, 2H, Ar-H, H-2′, H-6′), 7.42 (t, *J* = 7.2 Hz, 6H, Ar-H, H-3′, H-4′, H-5′, H-9, H-10, H-11), 7.25 (m, 3H, Ar-H, H-2, H-8, H-12), 7.05 (d, *J* = 7.8 Hz, 1H, Ar-H, H-6), 4.73 (q, *J =* 7.2 Hz, 1H, -CH-CH_3_), 1.86 (m, 6H, H-7′′, H-8′′, H-9′′), 1.50 (d, *J* = 7.2 Hz, 3H, CH_3_-CH-), 1.24 (m, 4H, H-10′′, H-11′′), ^13^C-NMR 150 MHz, CDCl_3_ δ (ppm): 175.0 (C-14), 158.8 (C-3), 158.0 (C-1′′), 135.7 (C-1), 133.2 (C-7), 132.7 (C-4′), 130.5 (C-6), 129.4 (C-4, C-8, C-12), 128.9 (C-3′, C-5′), 128.8 (C-2′, C-6′), 127.7 (C-10), 115.8 (C-2), 45.8 (C-13), 30.5 (C-8′, C-12′), 29.4 (C-10′), 26.1 (C-9′, C-11′) 18.2 (C-15). LC-HRMS (ESI^+^): Found [M + H]^+^: 429.2332; C_28_H_29_FN_2_O.

#### 3.4.23. (E)-2-(2-Fluoro-[1,1′-biphenyl]-4-yl)-N′-(1-phenylethylidene)propanehydrazide (**5f**)

Solid; yield: 71%; M.P.: 181 °C; ^1^H-NMR (600 MHz, CDCl_3_; ⸹, ppm): 8.55 (s, 1H, -NH), 7.95 (d, *J* = 7.8 Hz, 1H, Ar-H, H-5), 7.70 (d, *J* = 7.2 Hz, 2H, Ar-H, H-2′, H-6′), 7.55 (d, *J* = 7.2 Hz, 2H, Ar-H, H-3′, H-5′), 7.50 (d, *J* = 7.2 Hz, 2H, Ar-H, H-8, H-12), 7.45 (t, *J* = 7.2 Hz, 1H, Ar-H, H-10), 7.39 (m, 3H, -CH_3_), 4.80 (q, *J* = 7.2 Hz, 1H, -CH-CH_3_), 2.17 (s, 3H, -CH_3_), 1.57 (d, *J* = 6.6 Hz, 3H, CH_3_-CH-), ^13^C-NMR 150 MHz, CDCl_3_ δ (ppm): 175.8 (C-14), 160.5 (C-3), 158.8 (C-16), 147.2 (C-1′), 142.8 (C-1), 137.8 (C-7), 135.6 (C-4′), 133.1 (C-5), 130.6 (C-9, C-11), 129.6 (C-3′, C-5′), 128.9 (C-4, C-8, C-12), 127.8 (C-2′, C-6′), 126.1 (C-10), 126.1 (C-10), 123.9 (C-6), 115.7 (C-2), 41.3 (C-13), 18.3 (C-15), 12.7 (C-17). LC-HRMS (ESI^+^): Found [M + H]^+^: 361.1707; C_23_H_21_FN_2_O.

#### 3.4.24. (E)-2-(2-Fluoro-[1,1′-biphenyl]-4-yl)-N′-(1-phenylpropylidene)propanehydrazide (**5g**)

Solid; yield: 78%; M.P.: 179 °C; ^1^H-NMR (600 MHz, CDCl_3_; ⸹, ppm): 7.95 (d, *J* = 7.8 Hz, 1H, Ar-H, H-5), 7.70 (d, *J* = 7.2 Hz, 2H, Ar-H, H-2′, H-6′), 7.54 (d, *J* = 6.6 Hz, 2H, Ar-H, H-8, H-12), 7.44 (t, *J* = 7.8 Hz, 6H, Ar-H, H-3′, H-4′, H-5′, H-9, H-10, H-11), 7.24 (s, 1H, Ar-H, H-2), 7.04 (m, 1H, Ar-H, H-6), 2.99 (q, *J* = 7.2 Hz, 1H, -CH-CH_3_), 1.41 (m, 2H, -CH_2_-), 1.15 (t, *J* = 7.8 Hz, 3H, CH_3_-CH_2_-), ^13^C-NMR 150 MHz, CDCl_3_ δ (ppm): 200.9 (C-14), 136.9 (C-7), 132.8 (C-1), 129.5 (C-9, C-11), 128.9 (C-4, C-8, C-12), 128.8 (C-5), 127.9 (C-2′, C-6′), 126.1 (C-4′), 31.7 (C-17), 19.4 (C-13), 8.2 (C-18). LC-HRMS (ESI^+^): Found [M + H]^+^: 375.1858; C_24_H_23_FN_2_O.

#### 3.4.25. (E)-2-(2-Fluoro-[1,1′-biphenyl]-4-yl)-N′-(2-methyl-1-phenylpropylidene)propanehydrazide (**5h**)

Solid; yield: 67%; M.P.: 177 °C; ^1^H-NMR (600 MHz, MeOD; ⸹, ppm): 7.55 (m, 4H, Ar-H, H-5, H-6, H-2′, H-6′), 7.49 (t, *J* = 6.6 Hz, 4H, Ar-H, H-4′, H-9, H-10, H-11), 7.41 (t, *J* = 7.2 Hz, 2H, Ar-H, H-3′, H-5′), 7.31 (m, 2H, H-8, H-12), 7.04 (m, 1H, Ar-H, H-2), 2.90 (m, 1H, H-2′′), 1.17 (m, 6H, 2 x –CH_3_), ^13^C-NMR 150 MHz, MeOD δ (ppm): 177.2 (C-14), 172.1 (C-3), 166.9 (C-1′′), 161.7 (C-1), 161.4 (C-7), 160.4 (C-4′), 136.9 (C-1′), 134.6 (C-5), 132.2 (C-10), 131.8 (C-6), 130.5 (C-9, C-11), 129.9 (C-2′, C-6′), 129.5 (C-8, C-12), 128.3 (C-3′, C-5′), 125.1 (C-2), 45.5 (C-13), 42.7 (C-2′′), 20.5 (C-3′′, C-4′′), 18.6 (C-15). LC-HRMS (ESI^+^): Found [M + H]^+^: 389.2032; C_25_H_25_FN_2_O.

#### 3.4.26. (E)-2-(2-Fluoro-[1,1′-biphenyl]-4-yl)-N′-(1-phenylpentylidene)propanehydrazide (**5i**)

Solid; yield: 89%; M.P.: 165 °C; ^1^H-NMR (600 MHz, MeOD; ⸹, ppm): 7.82 (s, 1H, -NH), 7.75 (d, *J* = 6.6 Hz, 1H, Ar-H, H-5), 7.54 (d, *J* = 7.8 Hz, Ar-H, 2H, H-2′, 6′), 7.50 (t, *J* = 8.4 Hz, 2H, Ar-H, H-9, H-11), 7.46 (t, *J* = 8.4 Hz, 2H, Ar-H, H-3′, H-5′), 7.41 (m, 4H, Ar-H, H-4′, H-8, H-10, H-12), 7.26 (d, *J* = 7.8 Hz, 1H, Ar-H, H-6), 7.21 (m, 1H, Ar-H, H-2), 4.06 (q, *J* = 7.2 Hz, 1H, -CH-CH_3_), 2.87 (m, 2H, H-2′′), 2.63 (t, *J* = 7.2 Hz, 1H, H-1′′), 1.35 (m, 4H, H-3′′, H-4′′), 0.90 (t, *J* = 7.2 Hz, 3H, H-5′′), ^13^C-NMR 150 MHz, MeOD δ (ppm): 173.2 (C-14), 160.9 (C-7′), 154.4 (C-3), 145.0 (C-1), 144.5 (C-7), 138.8 (C-3′), 138.5 (C-5), 136.9 (C-10), 132.0 (C-6), 131.7 (C-2), 129.9 (C-9, C-11), 129.5 (C-2′, C-6′), 129.4 (C-5, C-12), 128.7 (C-4), 127.5 (C-4′), 125.1 (C-13), 11.4 (C-1′), 29.7 (C-9′), 27.9 (C-8′, C-10′), 23.6 (C-15), 14.2 (C-11′). LC-HRMS (ESI^+^): Found [M + H]^+^: 403.2188; C_26_H_27_FN_2_O.

#### 3.4.27. (E)-N′-(1-Cyclopropylethylidene)-2-(2-fluoro-[1,1′-biphenyl]-4-yl)propanehydrazide (**5j**)

Solid; yield: 76%; M.P.: 160 °C; ^1^H-NMR (600 MHz, MeOD; ⸹, ppm): 7.53 (d, *J* = 7.2 Hz, 2H, Ar-H, H-8, H-12), 7.45 (t, *J* = 7.2 Hz, 3H, Ar-H, H-9, H-10, H-11), 7.38 (d, *J* = 7.2 Hz, 1H, Ar-H, H-5), 7.30 (m, 3H, H-1′, H-2′), 0.85 (m, 2H, H-2′), ^13^C-NMR 150 MHz, MeOD δ (ppm): 177.5 (C-14), 172.6 (C-4′), 165.2 (C-3), 161.7 (C-1), 160.1 (C-7), 156.8 (C-5), 144.6 (C-6), 136.9 (C-10), 131.8 (C-2), 129.9 (C-8, C-12), 129.4 (C-9), 128.9 (C-11), 124.7 (C-4), 49.5 (C-13), 49.1 (C-5′), 45.0 (C-15), 19.1 (C-1′), 12.3 (C-2′, C-3′). LC-HRMS (ESI^+^): Found [M + H]^+^: 325.1712; C_20_H_21_FN_2_O.

#### 3.4.28. (E)-2-(2-Fluoro-[1,1′-biphenyl]-4-yl)-N′-(1-phenylbutylidene)propanehydrazide (**5K**)

Solid; yield: 85%; M.P.: 183 °C; ^1^H-NMR (600 MHz, MeOD; ⸹, ppm): 7.83 (d, *J* = 7.8 Hz, 2H, Ar-H, H-2′, H-6′), 7.75 (d, *J* = 7.2 Hz, 1H, Ar-H, H-5), 7.54 (d, *J* = 7.2 Hz, 2H, Ar-H, H-8, H-12), 7.50 (t, *J* = 8.4 Hz, 1H, Ar-H, H-4′), 7.46 (t, *J* = 7.2 Hz, 3H, Ar-H, H-9, H-10, H-11), 7.34 (m, 2H, Ar-H, H-3′, H-5′), 7.21 (m, 1H, Ar-H, H-6), 7.13 (m, 1H, Ar-H, H-2), 4.07 (q, *J* = 6.6 Hz, 1H, -CH-CH_3_), 2.76 (t, *J* = 7.8 Hz, 2H, H-2′′), 1.60 (m, 2H, H-3′′), 0.96 (m, 3H, H-4′′), ^13^C-NMR 150 MHz, MeOD δ (ppm): 178.2 (C-14), 173.3 (C-1′′), 136.9 (C-3), 131.9 (C-1), 130.7 (C-7), 130.6 (C-4′), 129.9 (C-5), 129.5 (C-9, C-11), 128.1 (C-2′, C-6′), 127.6 (C-8, C-12), 125.1 (C-6), 124.8 (C-2), 11.4 (C-3′), 49.4 (C-2′′), 45.1 (C-3′′), 29.8 (C-15),14.1 (C-4′′). LC-HRMS (ESI^+^): Found [M + H]^+^: 389.2024; C_25_H_25_FN_2_O.

#### 3.4.29. N′-Cyclohexylidene-2-(2-fluoro-[1,1′-biphenyl]-4-yl)propanehydrazide (**5l**)

Solid; yield: 80%; M.P.: 177 °C; ^1^H-NMR (600 MHz, MeOD; ⸹, ppm): 7.53 (d, *J* = 7.2 Hz, 2H, Ar-H, H-8, H-12), 7.54 (t, *J* = 7.8 Hz, 3H, Ar-H, H-9, H-10, H-11), 7.38 (m, 1H, Ar-H, H-5), 7.29 (m, 2H, Ar-H, H-2, H-6), 3.87 (q, *J* = 6.6 Hz, 1H, -CH-CH_3_), 2.44 (m, 2H, H-2′), 2.37 (t, *J* = 6.0 Hz, 2H, H-6′), 1.74 (m, 2H, H-4′), 1.68 (m, 4H, H-3′, H-5′), 1.54 (d, *J* = 7.2 Hz, 3H, CH_3_-CH-), ^13^C-NMR 150 MHz, MeOD δ (ppm): 177.7 (C-14), 172.9 (C-1′), 167.7 (C-3), 161.7 (C-1), 160.1 (C-7), 144.5 (C-5), 136.9 (C-6), 131.8 (C-10), 129.9 (C-9, C-11), 129.4 (C-8, C-12), 128.7 (C-2), 124.7 (4), 45.0 (C-13), 36.1 (C-6′), 28.7 (C-2′), 28.3 (C-3′), 27.3 (C-5′), 26.5 (C-4′), 19.1 (C-15). LC-HRMS (ESI^+^): Found [M + H]^+^: 339.1870; C_21_H_23_FN_2_O.

#### 3.4.30. (E)-2-(2-Fluoro-[1,1′-biphenyl]-4-yl)-N′-(1-(2-methoxyphenyl)ethylidene)propanehydrazide (**5m**)

Solid; yield: 78%; M.P.: 198 °C; ^1^H-NMR (600 MHz, MeOD; ⸹, ppm): 7.55 (t, *J* = 7.2 Hz, 2H, Ar-H, H-9, H-11), 7.49 (m, 2H, Ar-H, H-5, H-6), 7.41 (m, 2H, Ar-H, H-8, H-12), 7.33 (d, *J* = 9.0 Hz, 1H, Ar-H, H-3′), 7.22 (t, *J* = 8.4 Hz, 1H, Ar-H, H -4′), 7.05 (t, *J* = 6.6 Hz, 1H, Ar-H, H-5′), 4.02 (q, *J* = 7.2 Hz, 1H, -CH-CH_3_), 3.70 (s, 3H, CH_3_O), 1.59 (d, *J* = 7.2 Hz, 3H, CH_3_-CH-), ^13^C-NMR 150 MHz, MeOD δ (ppm): 178.1 (C-14), 158.9 (C-1′′), 132.4 (C-2′), 132.1 (C-3), 131.9 (C-1), 131.7 (C-7), 131.4 (C-4′), 130.6 (C-6′), 129.9 (C-9, C-11), 129.4 (C-8, C-12), 128.9 (C-6), 128.6 (C-5), 125.2 (C-2), 124.8 (C-1′), 122.2 (C-3′), 55.9 (C-7′), 42.5 (C-13). LC-HRMS (ESI^+^): Found [M + H]^+^: 391.1813; C_24_H_23_FN_2_O_2_.

#### 3.4.31. (E)-2-(2-Fluoro-[1,1′-biphenyl]-4-yl)-N′-(1-(4-methoxyphenyl)ethylidene)propanehydrazide (**5n**)

Solid; yield: 70%; M.P.: 192 °C; ^1^H-NMR (600 MHz, MeOD; ⸹, ppm): 7.92 (d, *J* = 7.8 Hz, 2H, Ar-H, H-2′, H-6′), 7.53 (d, *J* = 7.8 Hz, 1H, Ar-H, H-5), 7.43 (t, *J* = 7.2 Hz, 2H, Ar-H, H-9, H-11), 7.35 (d, *J* = 7.2 Hz, 1H, Ar-H, H-6), 7.11 (m, 1H, Ar-H, H-2), 6.92 (d, *J* = 8.4 Hz, 2H, Ar-H, H-3′, H-5′), 4.02 (q, *J* = 7.2 Hz, 1H, -CH-CH_3_), 3.85 (s, 3H, CH_3_O), 2.54 (s, 3H, -CH_3_), 1.65 (d, *J* = 7.2 Hz, 3H, CH_3_-CH-), ^13^C-NMR 150 MHz, MeOD δ (ppm): 196.8 (C-14), 163.6 (C-4′), 132.4 (C-1′′), 130.6 (C-9, C-11), 128.9 (C-3), 128.4 (C-7), 127.8 (C-1), 127.8 (C-1), 113.6 (C-8, C-12), 55.4 (C-7′), 26.3 (C-2′′), 17.9 (C-15). LC-HRMS (ESI^+^): Found [M + H]^+^: 391.1819; C_24_H_23_FN_2_O_2_.

### 3.5. In Vitro α-Glucosidase Inhibition Assay

α-Glucosidase (E.C.3.2.1.20) enzyme inhibition assay was developed by using 0.1 M phosphate buffer (pH 6.8) at 37 °C [39,40,41]. The enzyme (0.2 μ/mL) was incubated in phosphate-buffered saline with different concentrations of tested compounds at 37 °C for 15 min. The substrate (0.7 mM, p-nitrophenyl-α-d-glucopyranoside) was then added and the variation in absorbance at 400 nm was observed for 30 min using a spectrophotometer (xMark™ Microplate Spectrophotometer, BIO-RAD). Test compounds were substituted with DMSO-d6 (7.5% final) in the control. Acarbose was used as the standard inhibitor. The % inhibition was calculated by using the following formula:

% Inhibition = 100 − (OD test well/OD control) × 100.

### 3.6. Molecular Docking

The docking study of the active compounds was conducted in molecular operating environment [42] on the X-ray crystal structure of isomaltase from Saccharomyces cerevisiae in complex with alpha-D-glucopyranose (PDB code: 3A4A, resolution: 1.60 Å) [43]. In our previous studies [11,40,44], we have thoroughly checked the docking performance of applied docking protocol via re-docking of acarbose in the active site of α-glucosidase where MOE was found efficient with an RMSD value of 0.36 Å. The enzyme file was handled through QuickPrep module of MOE to add missing hydrogen atoms on each residue and to calculate partial charges using pre-defined force field (Amber10:EHT force field). The structures of active compounds were drawn by ChemDraw and converted into three-dimensional (3D)-format by MOE-WASH module after importing all the prepared structures in the MOE database where hydrogen atoms and partial charges were added to all the compounds simultaneously and the structure of each ligand was minimized until RMS gradient of 0.1 RMS kcal/mol/Å was obtained. In subsequent step, triangle matcher docking algorithm in combination with London dG scoring function of MOE was applied during docking. In the end, the best-docked conformation of each ligand was selected based on the good docking score and appropriate binding interaction.

## 4. Conclusions

Novel acyl hydrazones of flurbiprofen (**4a-4p**) and (**5a-5n**) were synthesized and evaluated for in vitro inhibition against the α-glucosidase enzyme. Among the synthesized compounds, **5k**, **4h**, **5h**, **4d**, **4b**, and **5i** showed excellent inhibitory potential. The structure–activity relationship (SAR) advocated that the deviation in the activity of the synthesized compounds may be due to the attachment and nature of substituents. Likewise, in silico studies have shown that the synthesized hydrazones (ligands) have widespread binding interactions within the active site of the enzyme, and due to their different substituent, their conformation is different in the active site of the enzyme. This study recognized numerous lead candidates derived from flurbiprofen and further exploration of these molecules for coming research to obtain novel α-glucosidase inhibitors.

## Figures and Tables

**Table 1:** α-Glucosidase inhibitory potential of Schiff’s bases of flurbiprofen.

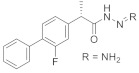 1					
Compounds	R	IC_50_ ± µM (S.E.M)	Compounds	R	IC_50_ ± µM (S.E.M)
**4a**	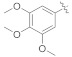	N/A	**5a**	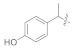	N/A
**4b**	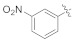	7.16 ± 3.25	**5b**	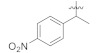	N/A
**4c**	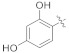	48.39 ± 3.75	**5c**	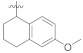	35.52 ± 2.04
**4d**	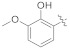	4.77 ± 2.03	**5d**	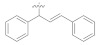	42.03 ± 3.51
**4e**	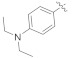	N/A	**5e**	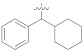	N/A
**4f**	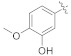	426.82 ± 4.63	**5f**	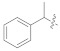	136.36 ± 2.57
**4g**	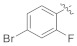	11.42 ± 1.09	**5g**	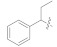	68.75 ± 1.42
**4h**	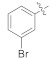	1.52 ± 0.07	**5h**	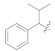	3.41 ± 0.05
**4i**	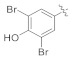	13.20 ± 2.47	**5i**	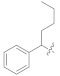	10.26 ± 0.13
**4j**	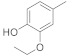	18.71 ± 0.25	**5j**	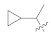	29.31 ± 0.60
**4k**	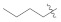	146.78 ± 0.86	**5k**	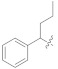	0.93 ± 0.06
**4l**	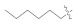	17.36 ± 1.68	**5l**	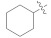	14.73 ± 0.27
**4m**	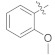	19.84 ± 1.89	**5m**	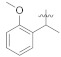	11.83 ± 0.18
**4n**	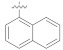	147.26 ± 2.78	**5n**	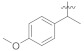	14.24 ± 0.16
**4o**	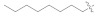	22.72 ± 1.46		**Acarbose**	875.75 ± 1.24
**4p**	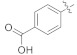	18.61 ± 0.14			

NA = Not active; S.E.M = standard error mean.

**Table 2:** Molecular Docking results of the Active α-glucosidase Inhibitors.

Compounds	Docking Score (kcal/mol)	Protein-Ligand Interaction			
		Ligand Atom	Receptor Atom	Interaction	Distance (Å)
**5k**	−7.51	N9 O8 6-ring	OE2-GLU277 NE2-HIS351 CE1-PHE178	HBD HBA π-H	1.97 1.74 3.21
**4h**	−6.98	N9 O8 6-ring	OE2-GLU277 NE2-HIS351 CD2-TYR158	HBD HBA π-H	1.84 1.69 2.72
**5h**	−6.71	O8 6-ring	NH2-ARG213 6-ring-PHE303	HBA π-π	2.65 2.49
**4d**	−6.41	N9 N11	OD1-ASP215 NE2-HIS351	HBD HBA	1.80 1.95
**4b**	−6.04	O26 6-ring	NE-ARG213 6-ring-PHE303	HBA π-π	3.09 3.75
**5i**	−5.70	N9 O8 6-ring	OE1-GLU277 NE2-HIS351 6-ring-PHE303	HBD HBA π-π	2.53 2.26 2.52
**4g**	−5.36	N9 O8	OE2-GLU277 NE2-HIS351	HBD HBA	2.62 2.67
**5m**	−5.70	N9 O8 6-ring 6-ring 6-ring	OE2-GLU277 ND2-ASN350 6-ring-TYR347 6-ring-PHE301 6-ring-PHE178	HBD HBA π-π π-π π-π	2.82 2.98 3.75 3.27 3.59
**4i**	−5.69	O8	NH2-ARG213	HBA	2.67
**5n**	−5.24	N9 O8	OE2-GLU277 NE2-HIS351	HBD HBA	1.84 1.70
**5l**	−5.70	N9 O8	OE2-GLU277 NE2-HIS351	HBD HBA	1.82 1.71
**4l**	−5.59	N9 O8	OE2-GLU277 NE2-HIS351	HBD HBA	2.09 2.59
**4p**	−5.59	N18 N17	OD1-ASP215 NE2-HIS351	HBD HBA	1.71 1.95
**4j**	−5.36	O8 O46	NE2-HIS351 OH-TYR347	HBA HBA	2.28 2.09
**4m**	−5.44	N9 O8	OE2-GLU277 NE2-HIS351	HBD HBA	1.95 1.73
**4o**	−2.12	O8	OH-TYR347	HBA	2.05
**1**	−5.11	N11 O8 6-ring	OD2-ASP69 NH1-ARG442 CD2-TYR158	HBD HBA π-H	1.96 1.93 3.64
**5j**	−4.77	N9 O8	OE2-GLU277 NE2-HIS351	HBD HBA	1.82 1.69
**5c**	−4.27	N9 O8	OE2-GLU277 NE2-HIS351	HBD HBA	1.85 1.70
**5d**	−4.24	6-ring 6-ring	CG-GLU277 6-ring-PHE303	π-H π-π	3.03 2.58
**4c**	−4.21	N9 O46 O8	OD1-ASP215 OD2-ASP352 NE2-HIS351	HBD HBD HBA	1.80 1.83 2.62
**5g**	−3.48	6-ring	6-ring TYR72	π-π	2.80
**5f**	−3.12	N9 O8	OE2-GLU277 NE2-HIS351	HBD HBA	2.08 2.44
**4k**	−3.50	O8	NH1-ARG442	HBA	2.46
**4n**	−3.64	N9	OD1-ASP215	HBD	2.73
**4f**	−3.36	6-ring	6-ring-PHE303	π-π	3.69

HBA = Hydrogen bond acceptor, HBD = Hydrogen bond donor.

## Data Availability

Data is contained within the article and Supplementary Materials.

## Supplementary Materials

The following are available online at https://www.mdpi.com/article/10.3390/ph15060672/s1, Figures S1–S16: ^1^H, ^13^C NMR and HRMS (ESI+) of compounds **4a**–**4p**, Figures S17–S30: ^1^H, ^13^C NMR and HRMS (ESI+) of compounds **5a**–**5n**.
