# Supplementary material for: Bio-Oriented Synthesis of Novel (S)-Flurbiprofen Clubbed Hydrazone Schiff’s Bases for Diabetic Management: In Vitro and In Silico Studies
Source: Pharmaceuticals (Basel). 2022 May 27;15(6):672. doi: 10.3390/ph15060672 (PMC9231348; doi:10.3390/ph15060672)
Supplement: Supplementary file 1 [file pharmaceuticals-15-00672-s001.zip › pharmaceuticals-1694702-supplementary.pdf]

Bio-Oriented Synthesis of Novel (S)-Flurbiprofen Clubbed Hydrazone Schiff's Bases for Diabetic Management: In Vitro and In Silico Studies

Aftab Alam [1], Mumtaz Ali [1], Najeeb Ur Rehman [2,*], Saeed Ullah [2,3], Sobia Ahsan Halim [2], Abdul Latif [1], Zainab [4], Ajmal Khan [2], Obaid Ullah [2], Shujaat Ahmad [5], Ahmed Al-Harrasi [2,*] and Manzoor Ahmad [1,*]

[1] Department of Chemistry, University of Malakand, Dir Lower P.O. Box 18800, Khyber Pakhtunkhwa, Pakistan; aftab.alam@uom.edu.pk (A.A.); mumtazali@uom.edu.pk (M.A.); drlatif2016@gmail.com (A.L.)

[2] Natural and Medical Sciences Research Center, University of Nizwa, PC 616, Birkat Al Mauz, Nizwa P.O. Box 33, Oman; saidkhan@unizwa.edu.om (S.U.); sobia_halim@unizwa.edu.om (S.A.H.); ajmalkhan@unizwa.edu.om (A.K.); obaidullah@unizwa.edu.om (O.U.)

[3] Husein Ebrahim Jamal Research Institute of Chemistry, International Center for Chemical and Biological Sciences, University of Karachi, Karachi 75270, Sindh, Pakistan

[4] College of Chemistry and Materials Science, Hebei Normal University, Shijiazhuang 050024, China; abidkhan4199@gmail.com

[5] Department of Pharmacy, Shaheed Benazir Bhutto University Sheringal, Dir Upper P.O. Box 18800, Khyber Pakhtunkhwa, Pakistan; shujaat@sbbu.edu.pk

* Correspondence: najeeb@unizwa.edu.om (N.U.R.); aharrasi@unizwa.edu.om (A.A.-H.); manzoorahmad@uom.edu.pk (M.A.)

Abstract: A new series of flurbiprofen derivatives **(4a–4p)** and **(5a–5n)** were synthesized with different aromatic or aliphatic aldehydes and ketones to produce Schiff's bases and their structures were confirmed through HR-ESI-MS, ^{1}H and ^{13}C-NMR spectroscopy. The α-glucosidase inhibitory activities of the newly synthesized compounds were scrutinized, in which six compounds **5k**, **4h**, **5h**, **4d**, **4b** and **5i** showed potent inhibition in the range of 0.93 to 10.26µM, respectively, whereas fifteen compounds **4c, 4g, 4i, 4j, 4l, 4m, 4o, 4p, 5c, 5d, 5j, 5l, 5m, 5n** and **1** exhibited significant inhibitory activity with IC_{50} in range of = 11.42 to 48.39 µM. In addition, compounds **5g, 5f**, **4k**, **4n**, and **4f** displayed moderate-to-low activities. The modes of binding of all the active compounds were determined through molecular docking approach which revealed that two residues, specifically Glu277 and His351 are important in the stabilization of the active compounds in the active site of α-glucosidase. Furthermore, these compounds block the active site with high binding energies (–7.51 to –3.36 kcal/mol) thereby inhibiting the function of the enzyme.

Keywords: (S)-flurbiprofen; Schiff's bases; α-glucosidase inhibition; molecular docking

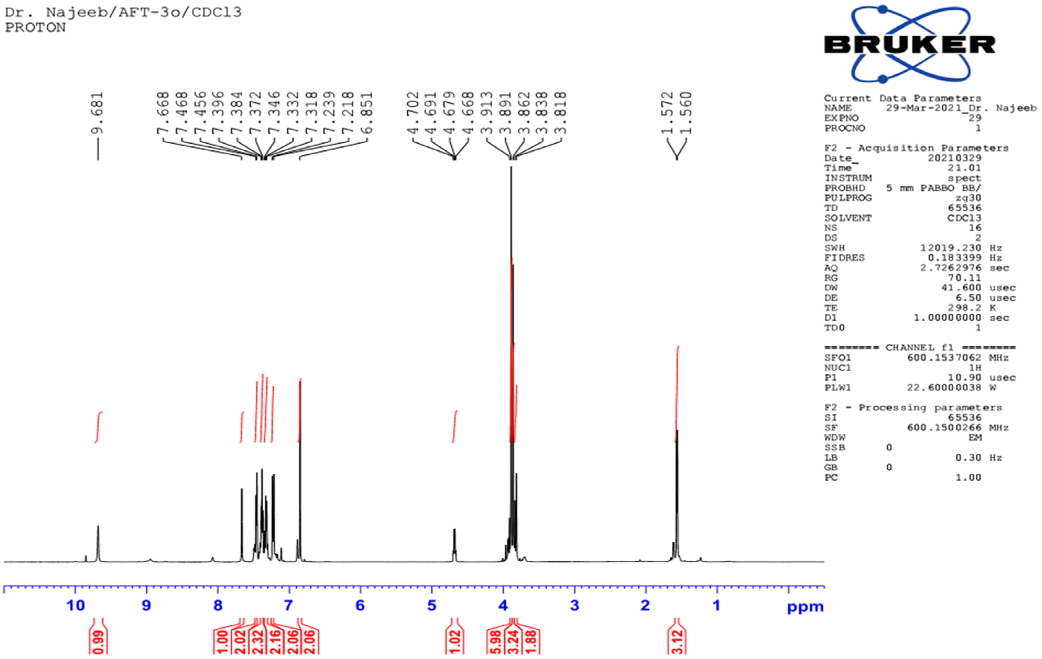
Dr. Najeeb/AFT-3o/CDCl3
PROTON
9.681
7.668
7.468
7.456
7.396
7.384
7.372
7.346
7.332
7.318
7.239
7.218
6.851
4.702
4.691
4.679
4.668
3.913
3.891
3.862
3.838
3.818
1.572
1.560
10
9
8
7
6
5
4
3
2
1
ppm
0.99
1.00
2.02
2.32
2.16
2.06
2.06
1.02
5.98
3.24
1.88
3.12
BRUKER
Current Data Parameters
NAME 29-Mar-2021_Dr. Najeeb
EXPNO 29
PROCNO 1
F2 - Acquisition Parameters
Date_ 20210329
Time 21.01
INSTRUM spect
PROBHD 5 mm PABBO BB/
PULPROG zg30
TD 65536
SOLVENT CDCl3
NS 16
DS 2
SWH 12019.230 Hz
FIDRES 0.183399 Hz
AQ 2.7262976 sec
RG 70.11
DW 41.600 usec
DE 6.50 usec
TE 298.2 K
D1 1.00000000 sec
TD0 1
======== CHANNEL f1 ========
SFO1 600.1537062 MHz
NUC1 1H
P1 10.90 usec
PLW1 22.60000038 W
F2 - Processing parameters
SI 65536
SF 600.1500266 MHz
WDW EM
SSB 0
LB 0.30 Hz
GB 0
PC 1.00

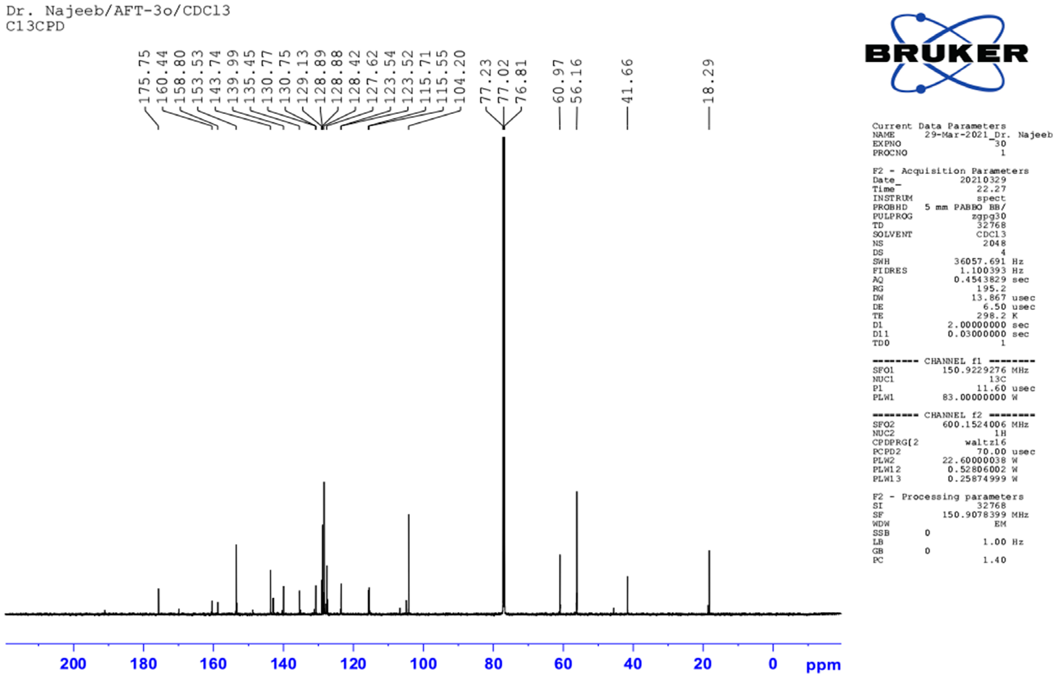
Dr. Najeeb/AFT-3o/CDCl3
C13CPD
175.75
160.44
158.80
153.53
143.74
139.99
135.45
130.77
130.75
129.13
128.89
128.88
128.42
127.62
123.54
123.52
115.71
115.55
104.20
77.23
77.02
76.81
60.97
56.16
41.66
18.29
200
180
160
140
120
100
80
60
40
20
0
ppm
BRUKER
Current Data Parameters
NAME 29-Mar-2021_Dr. Najeeb
EXPNO 30
PROCNO 1
F2 - Acquisition Parameters
Date_ 20210329
Time 22.27
INSTRUM spect
PROBHD 5 mm PABBO BB/
PULPROG zgpg30
TD 32768
SOLVENT CDCl3
NS 2048
DS 4
SWH 36057.691 Hz
FIDRES 1.100393 Hz
AQ 0.4543829 sec
RG 195.2
DW 13.867 usec
DE 6.50 usec
TE 298.2 K
D1 2.00000000 sec
D11 0.03000000 sec
TD0 1
======== CHANNEL f1 ========
SFO1 150.9229276 MHz
NUC1 13C
P1 11.60 usec
PLW1 83.00000000 W
======== CHANNEL f2 ========
SFO2 600.1524006 MHz
NUC2 1H
CPDPRG[2 waltz16
PCPD2 70.00 usec
PLW2 22.60000038 W
PLW12 0.52806002 W
PLW13 0.25874999 W
F2 - Processing parameters
SI 32768
SF 150.9078399 MHz
WDW EM
SSB 0
LB 1.00 Hz
GB 0
PC 1.40

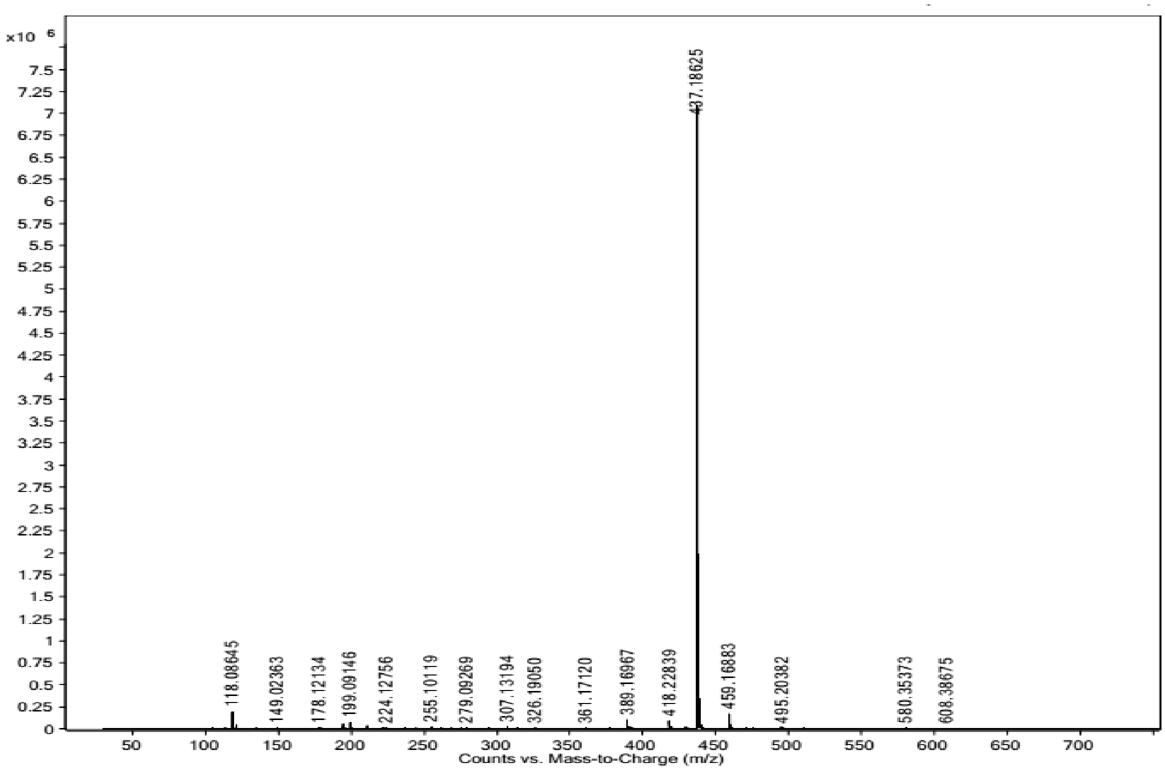

Figure S1: ^{1}H, ^{13}C NMR and HRMS (ESI^{+}) of compound **4a**

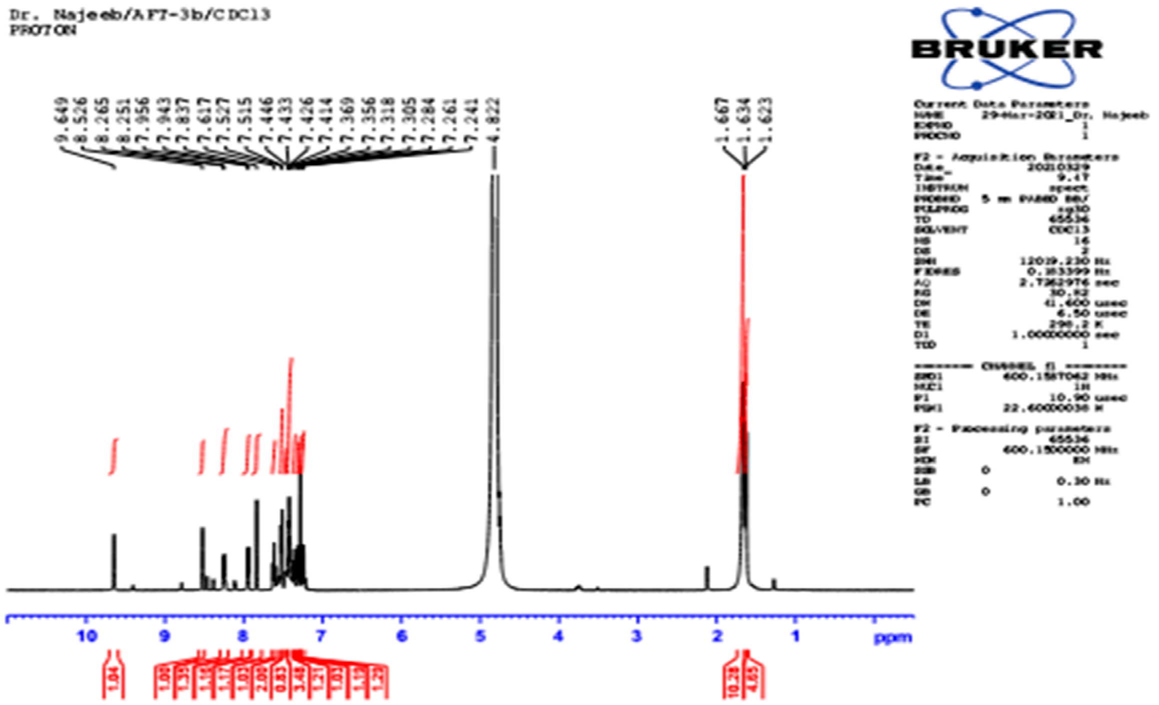

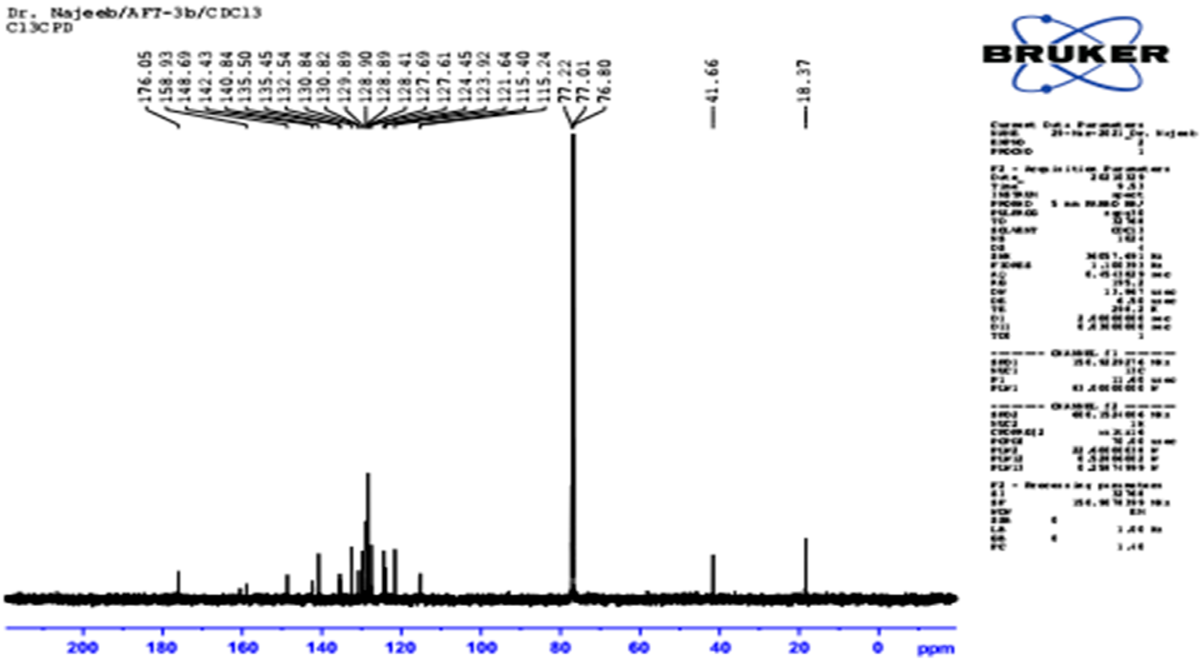

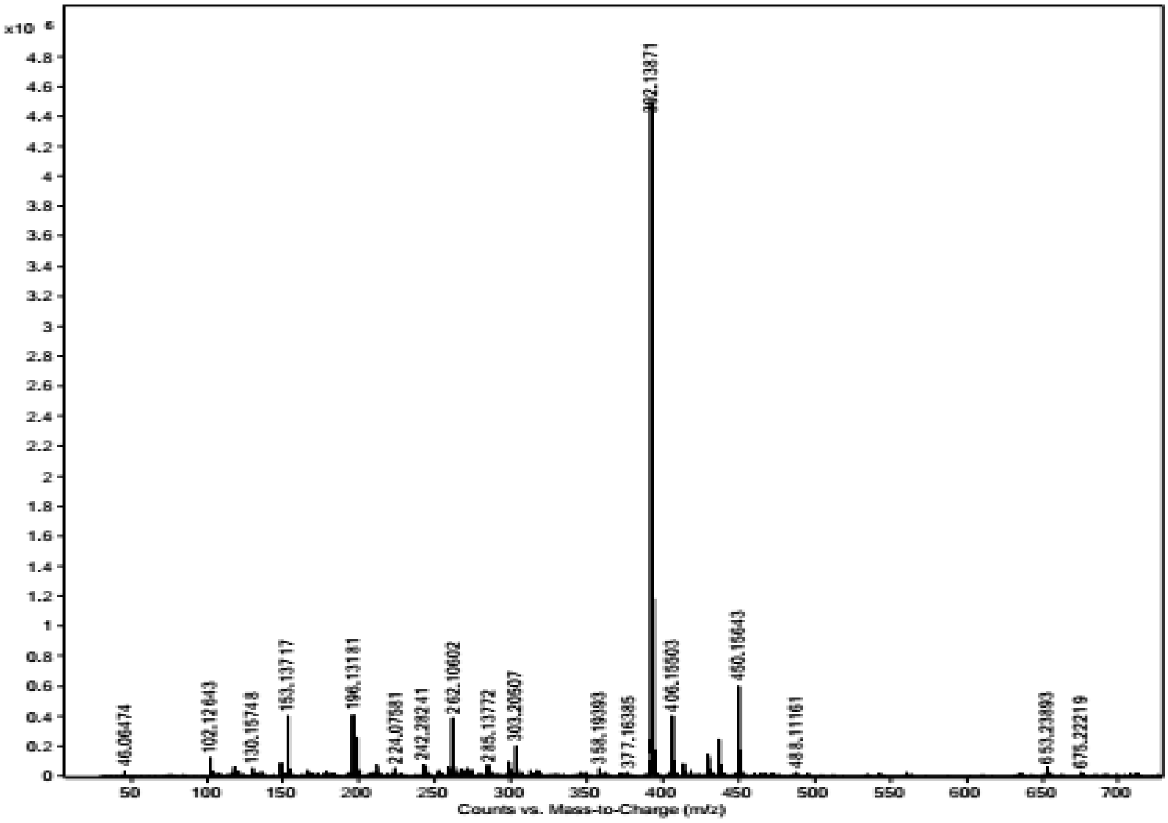

Figure S2: ^{1}H, ^{13}C NMR and HRMS (ESI^{+}) of compound **4b**

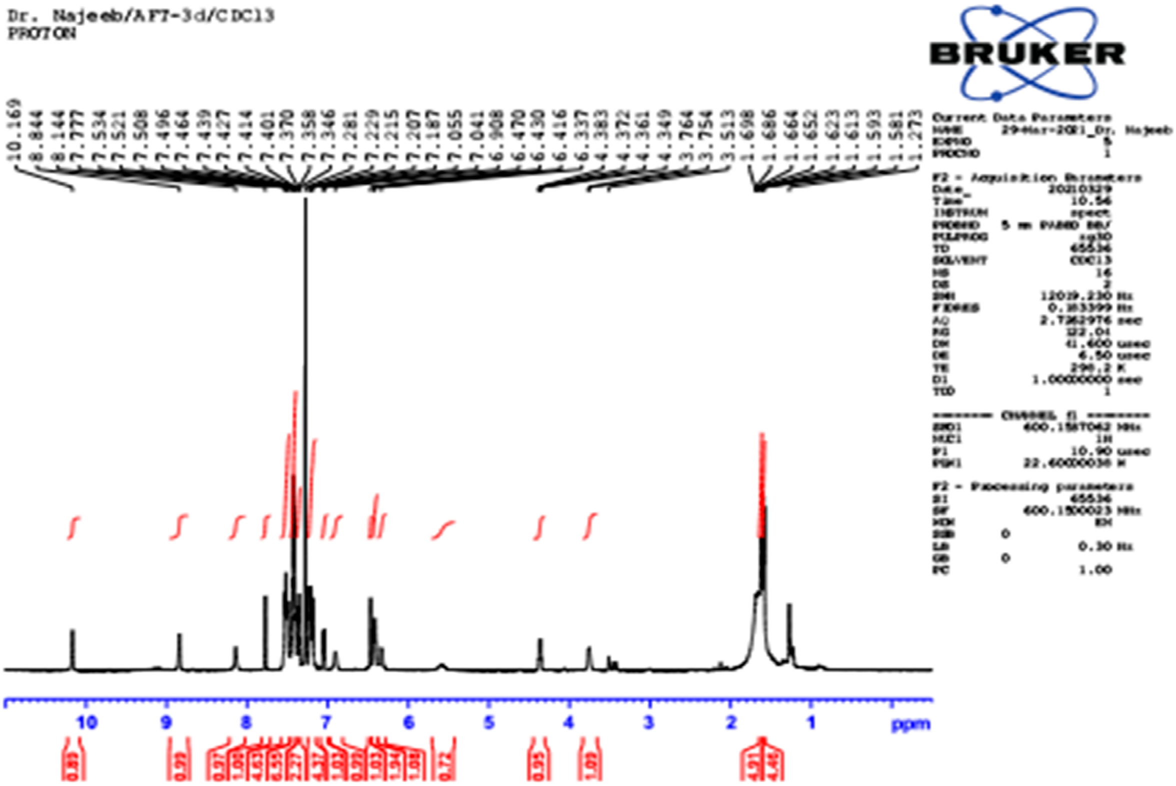
Dr. Najeeb/AFT-3d/CDCl3
PROTON
BRUKER
ppm

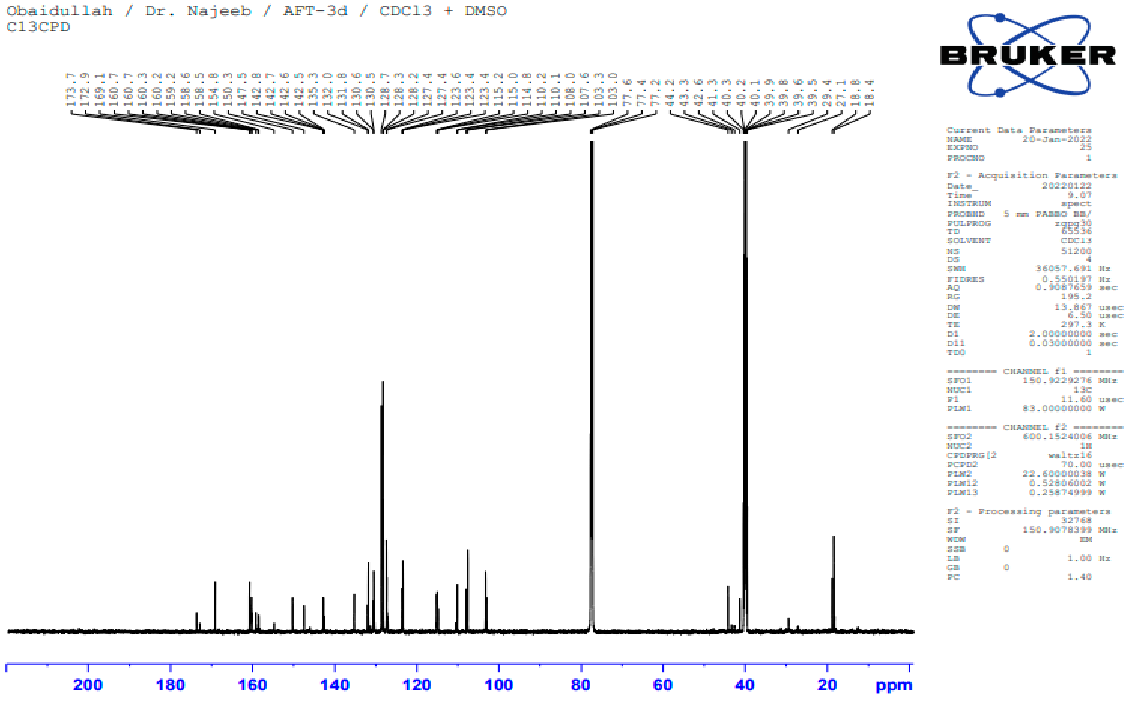
Obaidullah / Dr. Najeeb / AFT-3d / CDCl3 + DMSO
C13CPD
BRUKER
ppm

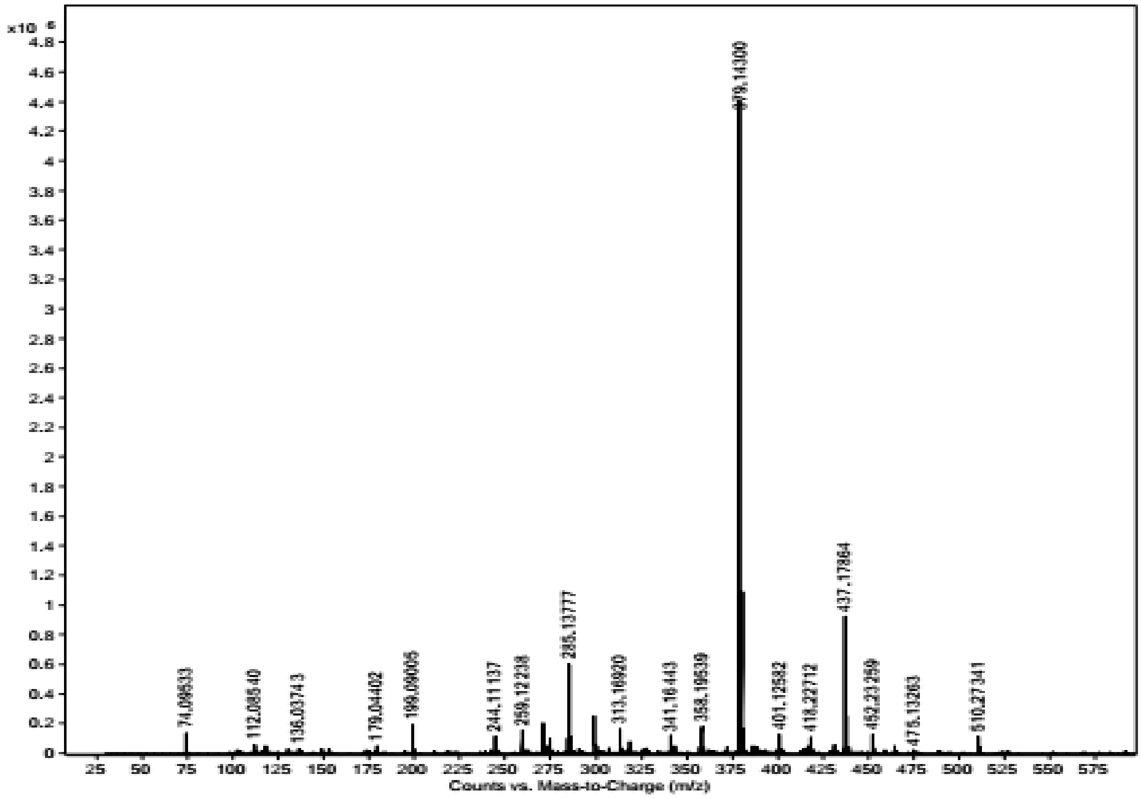

Figure S3: ^{1}H, ^{13}C NMR and HRMS (ESI^{+}) of compound **4c**

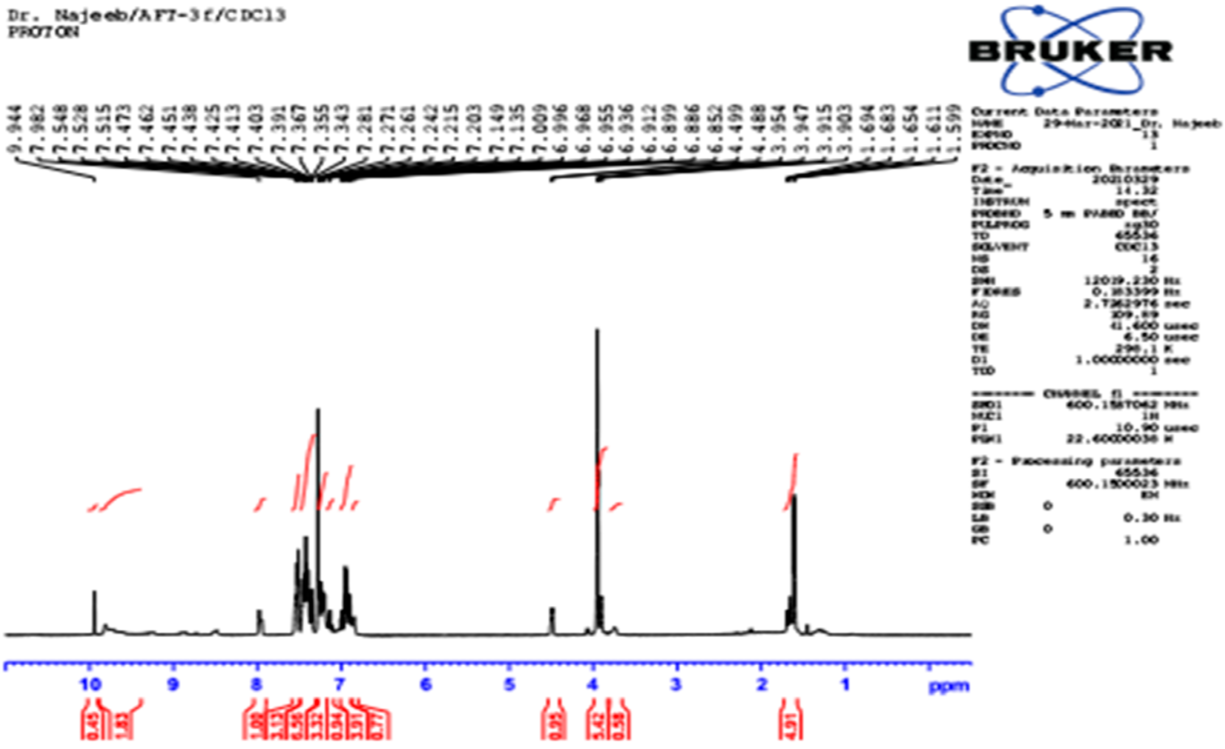

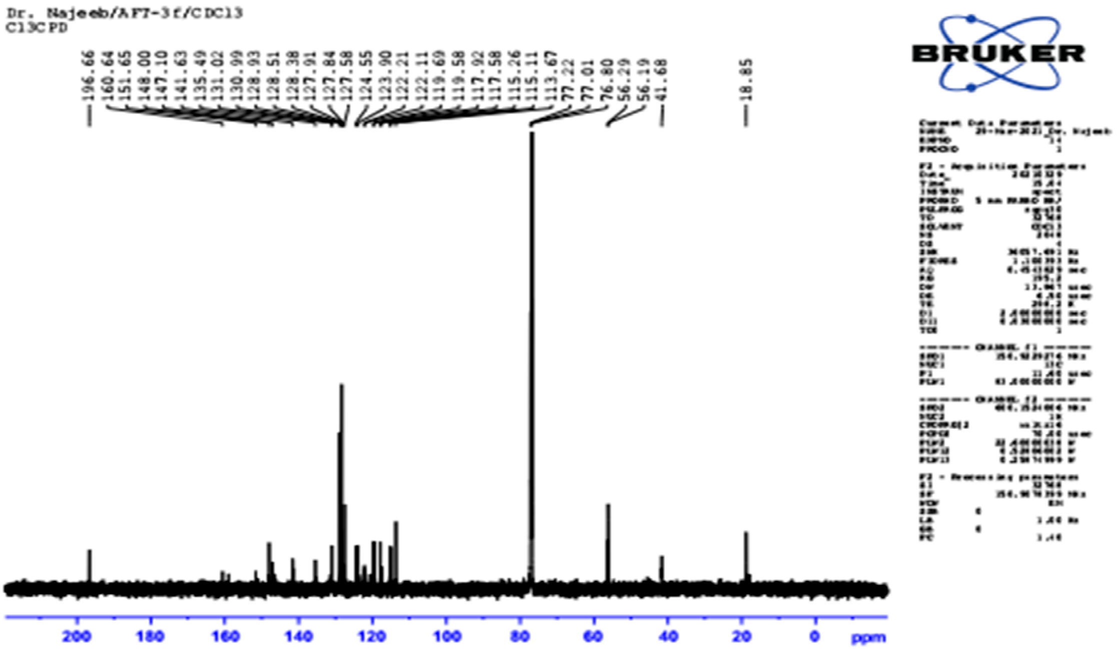

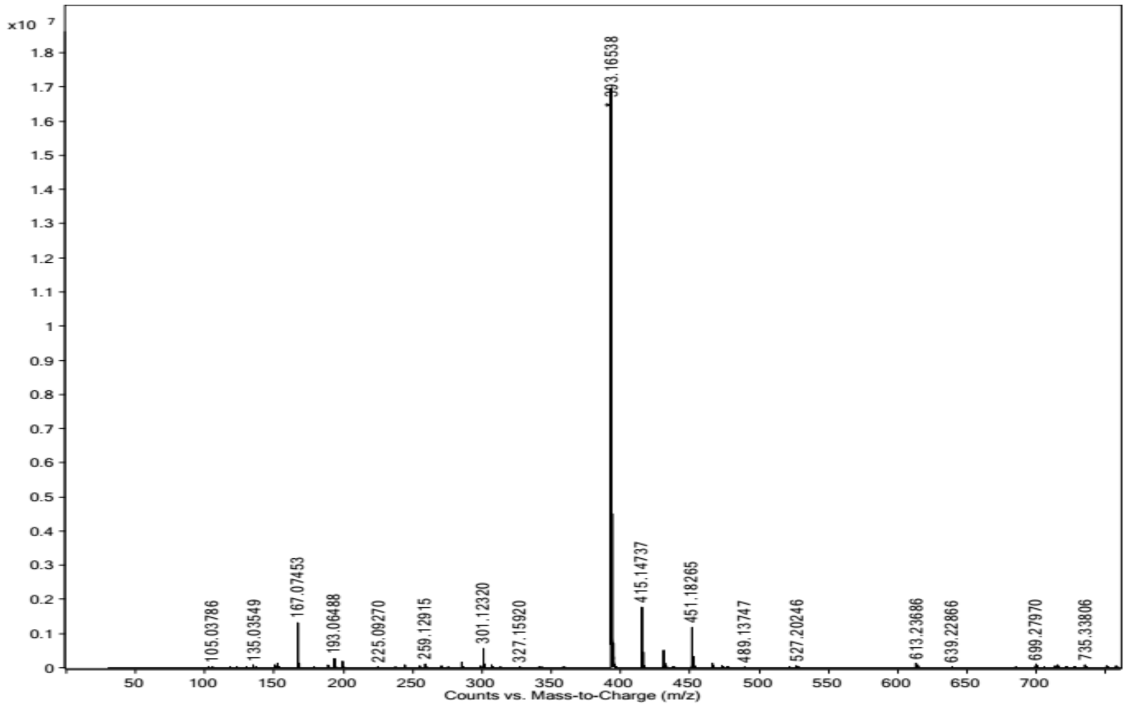

Figure S4: ^{1}H, ^{13}C NMR and HRMS (ESI^{+}) of compound **4d**

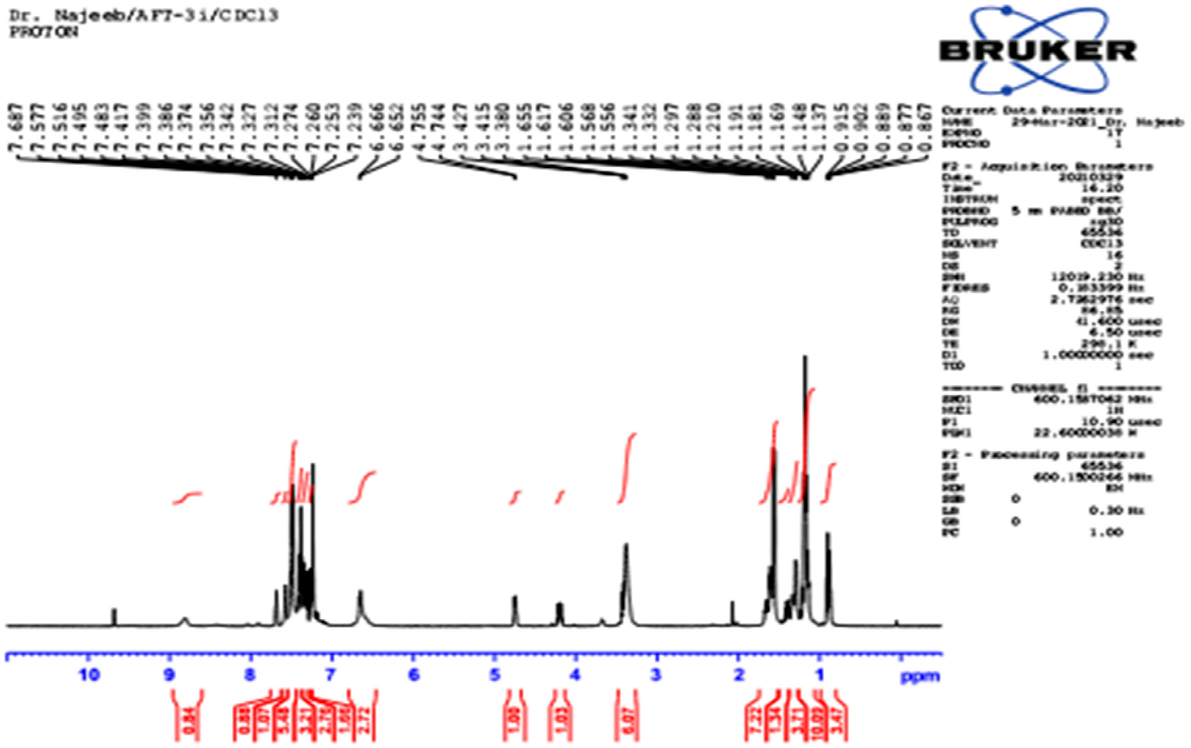
Dr. Najeeb/AF7-3i/CDCl3
PROTON
BRUKER
7.687 7.577 7.516 7.495 7.483 7.417 7.399 7.386 7.374 7.356 7.342 7.327 7.312 7.274 7.260 7.253 7.239 6.666 6.652 4.755 4.744 3.427 3.415 3.380 1.655 1.617 1.606 1.568 1.556 1.341 1.332 1.297 1.288 1.210 1.191 1.181 1.169 1.148 1.137 0.915 0.902 0.889 0.877 0.867
10 9 8 7 6 5 4 3 2 1 ppm

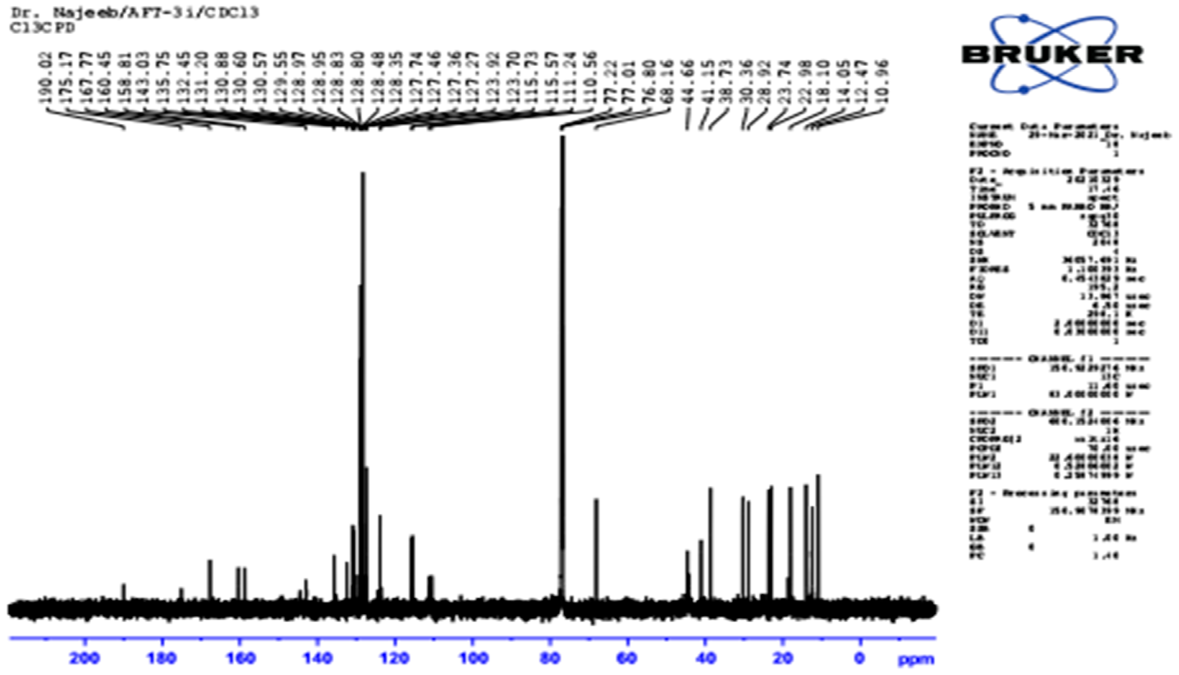
Dr. Najeeb/AF7-3i/CDCl3
C13CPD
BRUKER
190.02 175.17 167.77 160.45 158.81 143.03 135.75 132.45 131.20 130.88 130.60 130.57 129.55 128.97 128.95 128.83 128.80 128.48 128.35 127.74 127.46 127.36 127.27 123.92 123.70 115.73 115.57 111.24 110.56 77.22 77.01 76.80 68.16 44.66 41.15 38.73 30.36 28.92 23.74 22.98 18.10 14.05 12.47 10.96
200 180 160 140 120 100 80 60 40 20 0 ppm

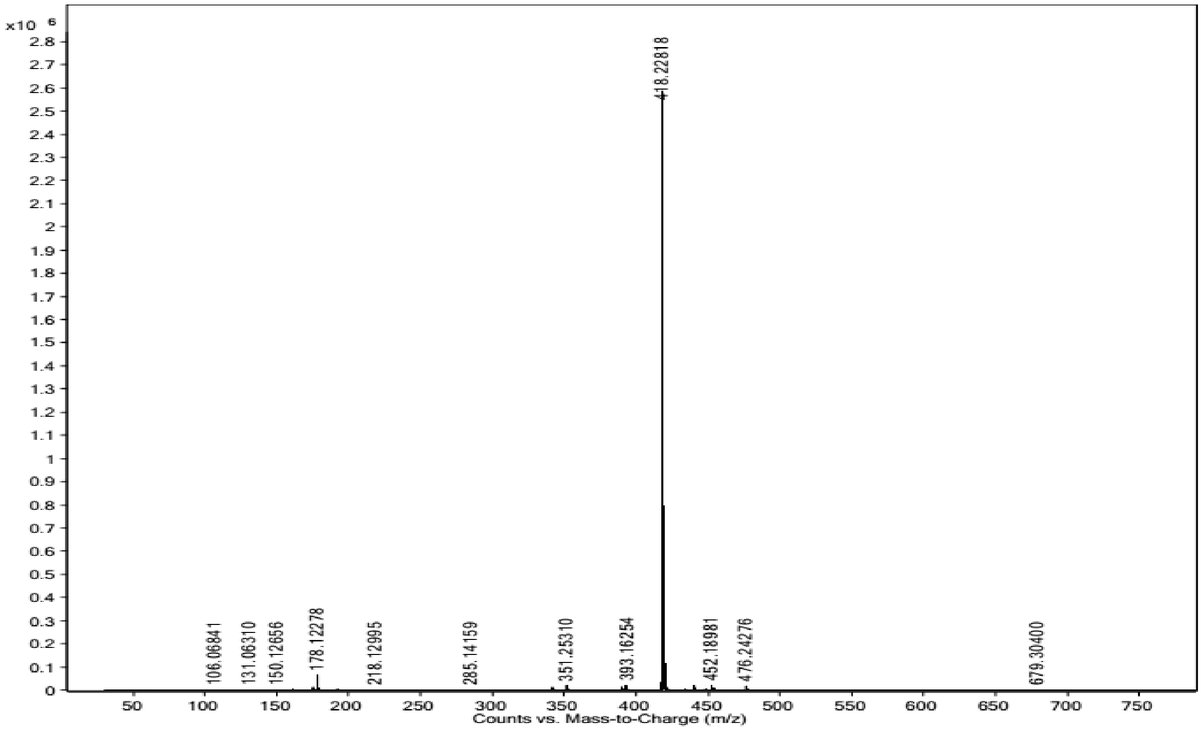

Figure S5: ^{1}H, ^{13}C NMR and HRMS (ESI^{+}) of compound **4e**

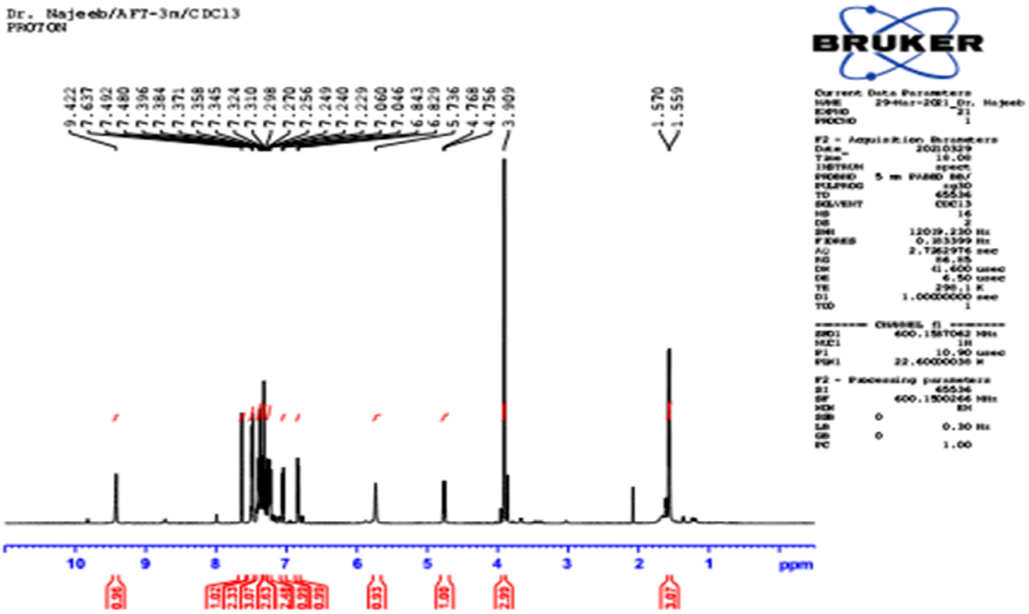

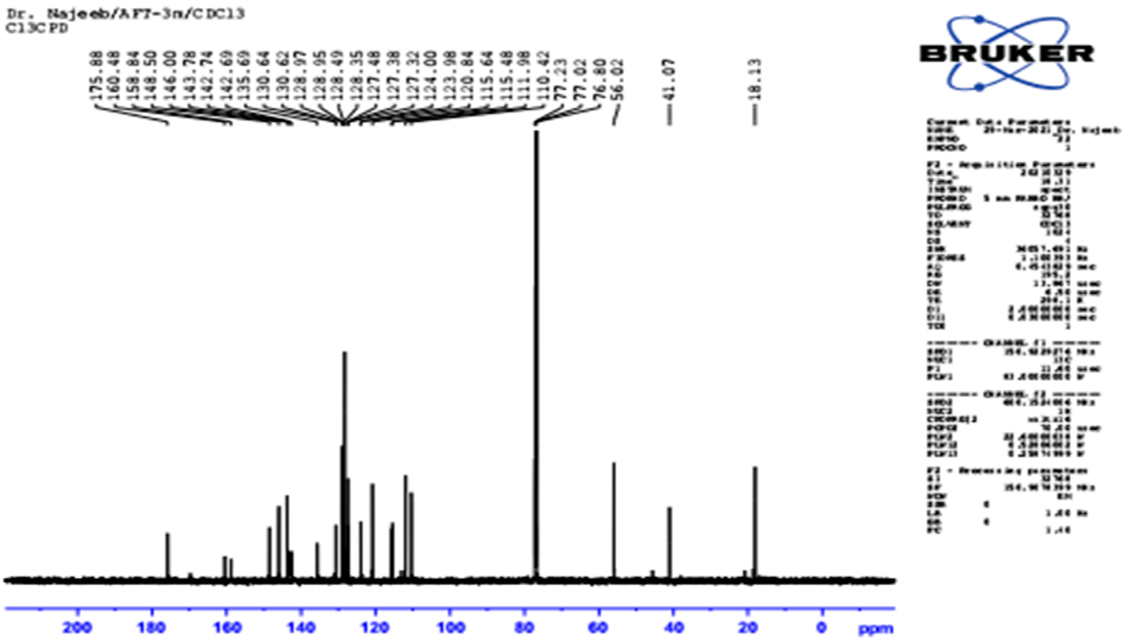

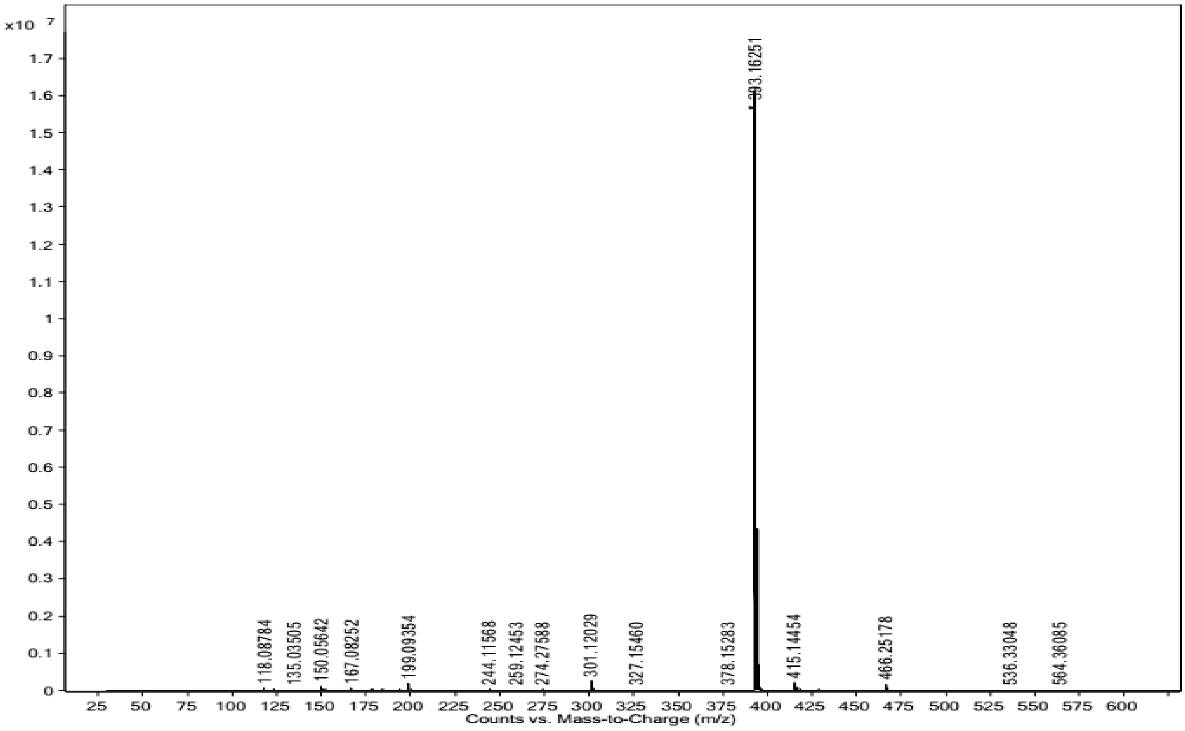

Figure S6: ^{1}H, ^{13}C NMR and HRMS (ESI^{+}) of compound **4f**

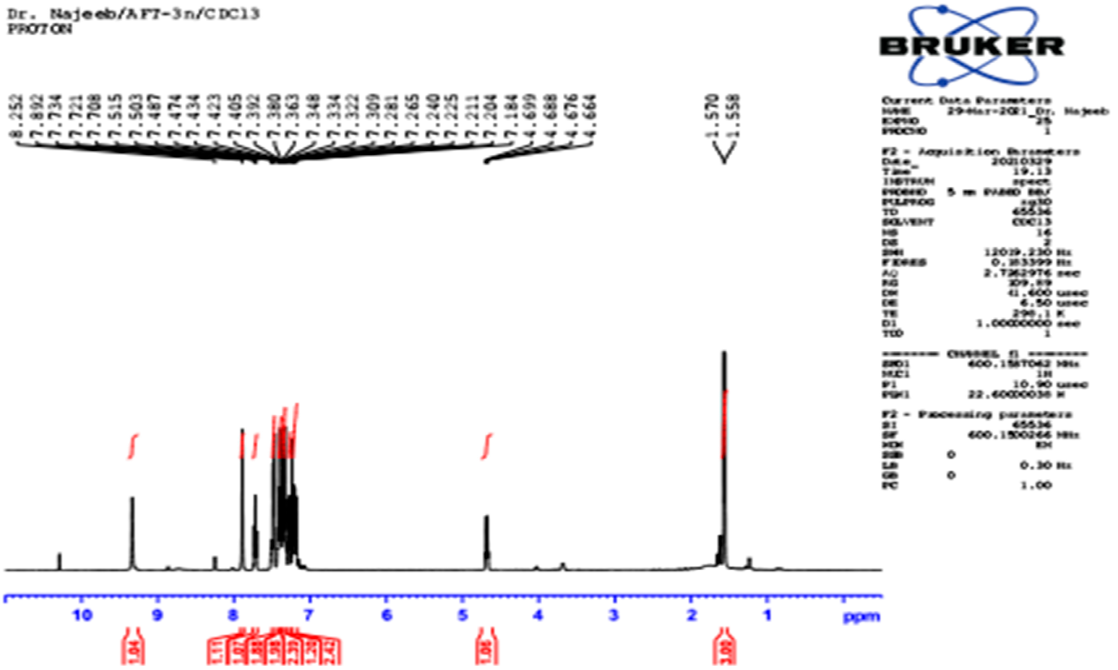

Dr. Najeeb/AF7-3n/CDCl3
PROTON
8.252
7.892
7.734
7.721
7.708
7.515
7.503
7.487
7.474
7.434
7.423
7.405
7.392
7.380
7.363
7.348
7.334
7.322
7.309
7.281
7.265
7.240
7.225
7.211
7.204
7.184
4.699
4.688
4.676
4.664
1.570
1.558
BRUKER
10 9 8 7 6 5 4 3 2 1 ppm

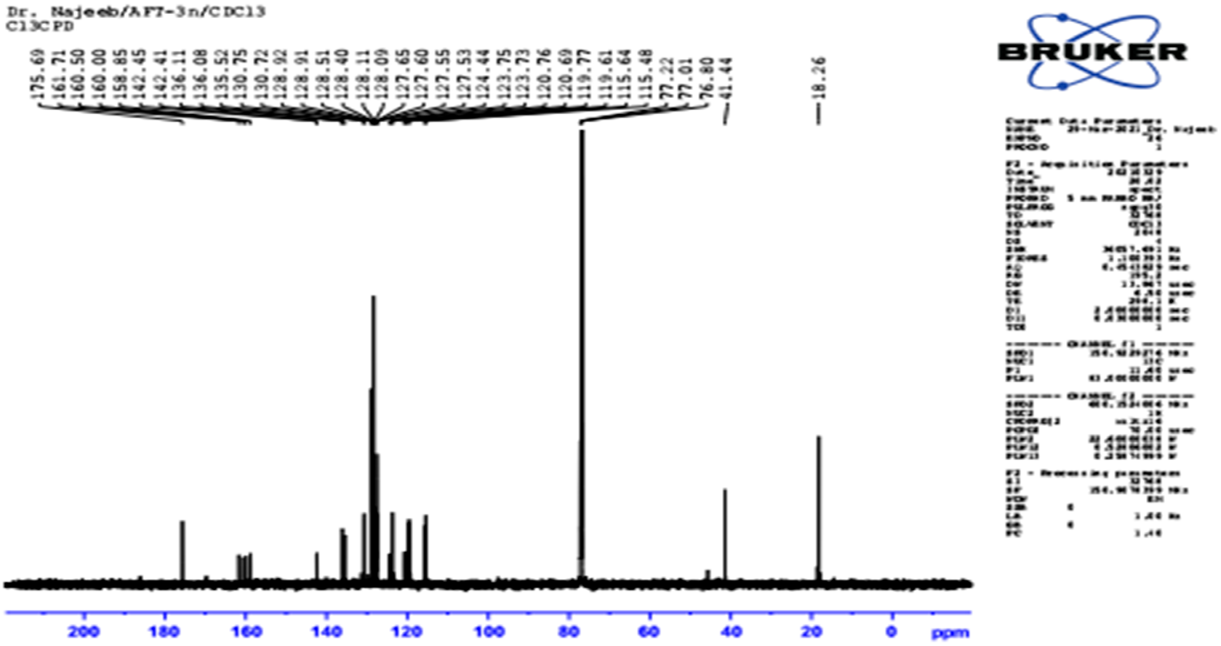

Dr. Najeeb/AF7-3n/CDCl3
C13CPD
175.69
161.71
160.50
160.00
158.85
142.45
142.41
136.11
136.08
135.52
130.75
130.72
128.92
128.91
128.51
128.40
128.11
128.09
127.65
127.60
127.55
127.53
124.44
123.75
123.73
120.76
120.69
119.77
119.61
115.64
115.48
77.22
77.01
76.80
41.44
18.26
BRUKER
200 180 160 140 120 100 80 60 40 20 0 ppm

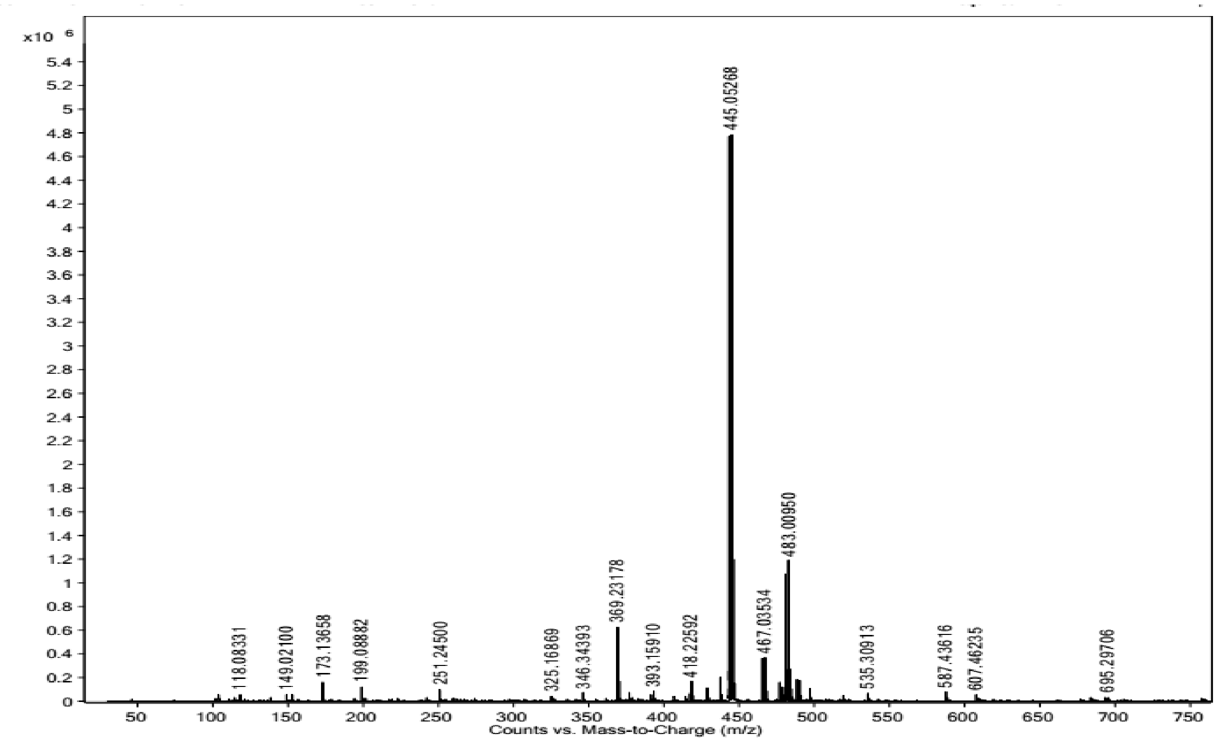

Figure S7: ^{1}H, ^{13}C NMR and HRMS (ESI^{+}) of compound **4g**

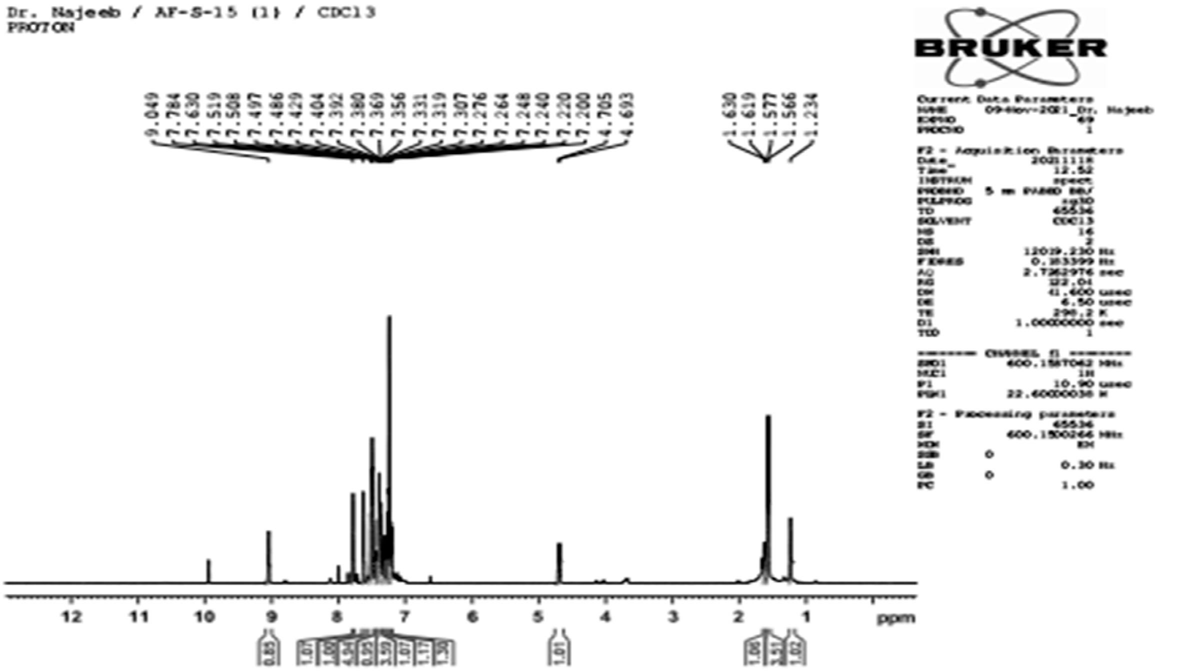

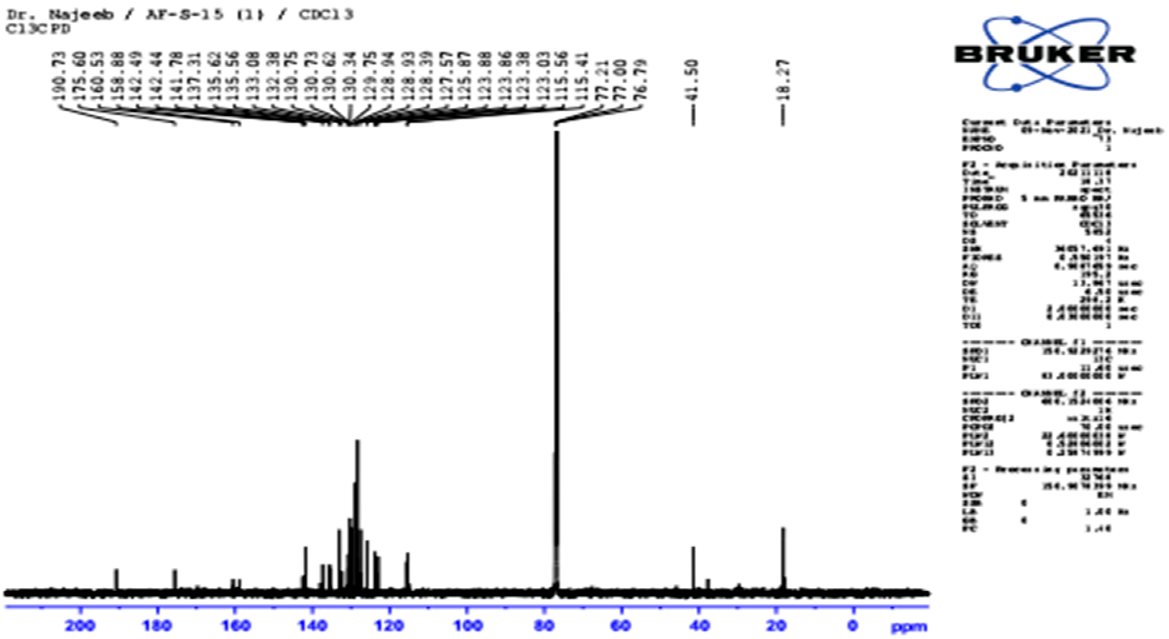

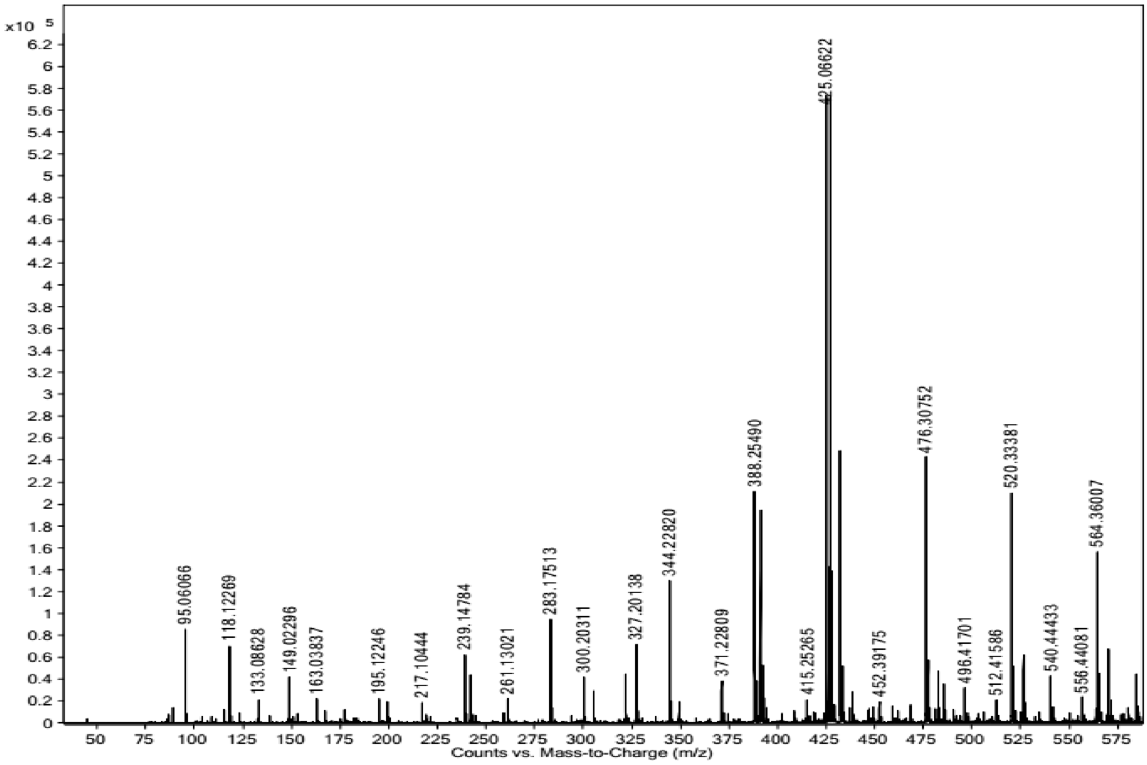

Figure S8: ^{1}H, ^{13}C NMR and HRMS (ESI^{+}) of compound **4h**

Dr. Najeeb/AF7-3p/CDCl3
PROTON

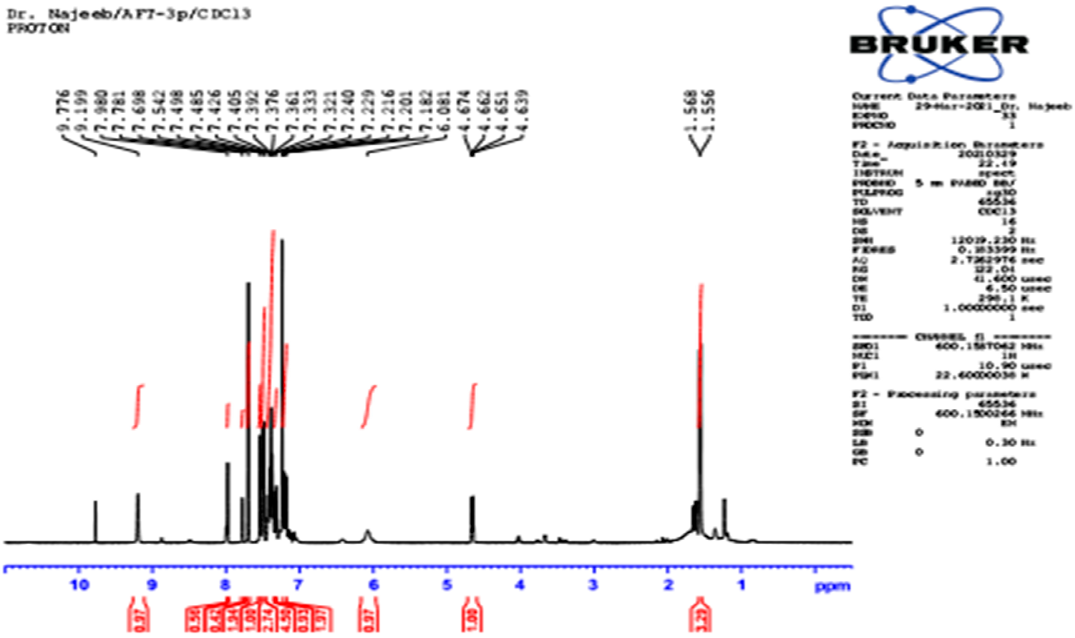

Dr. Najeeb/AF7-3p/CDCl3
C13CPD

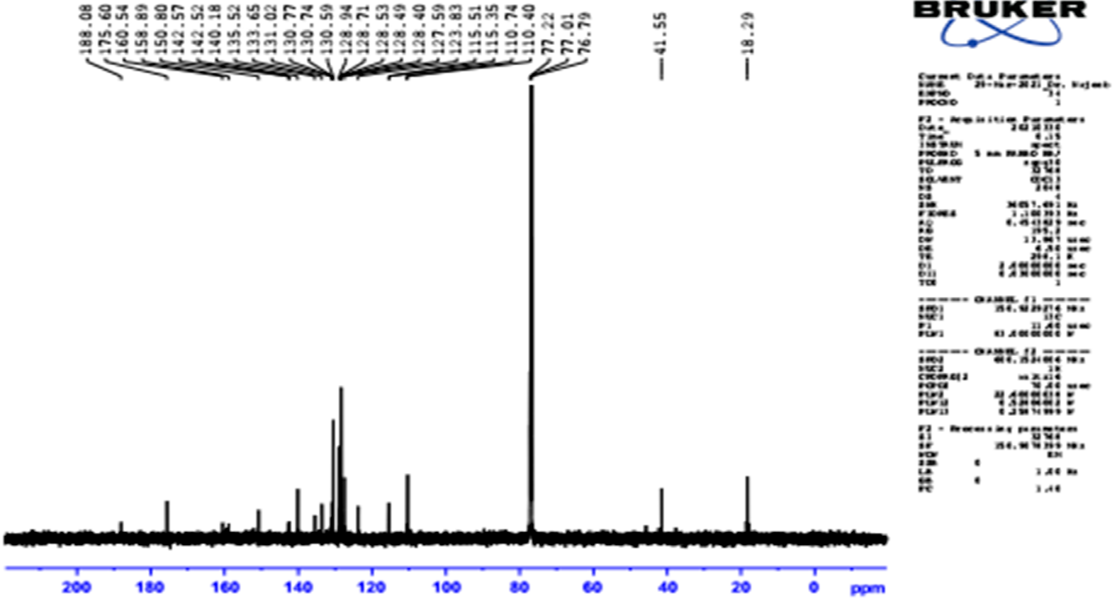

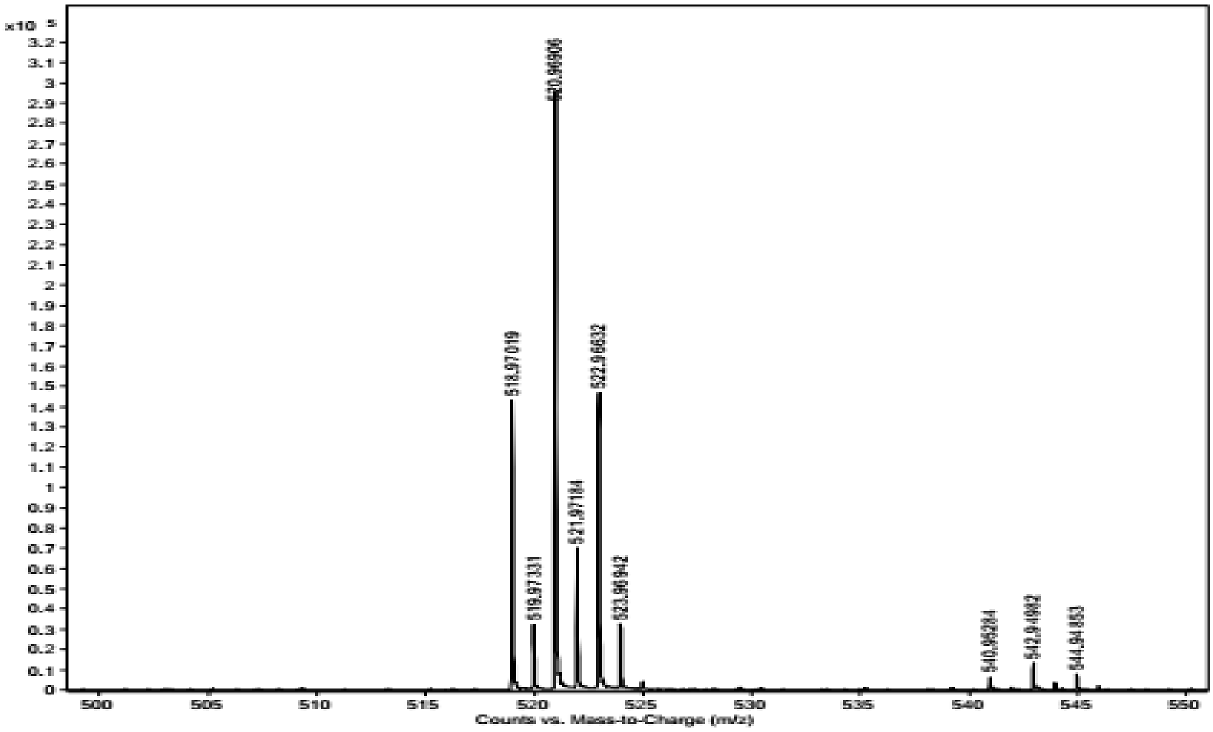

Figure S9. ^{1}H, ^{13}C NMR and HRMS (ESI^{+}) of compound **4i**

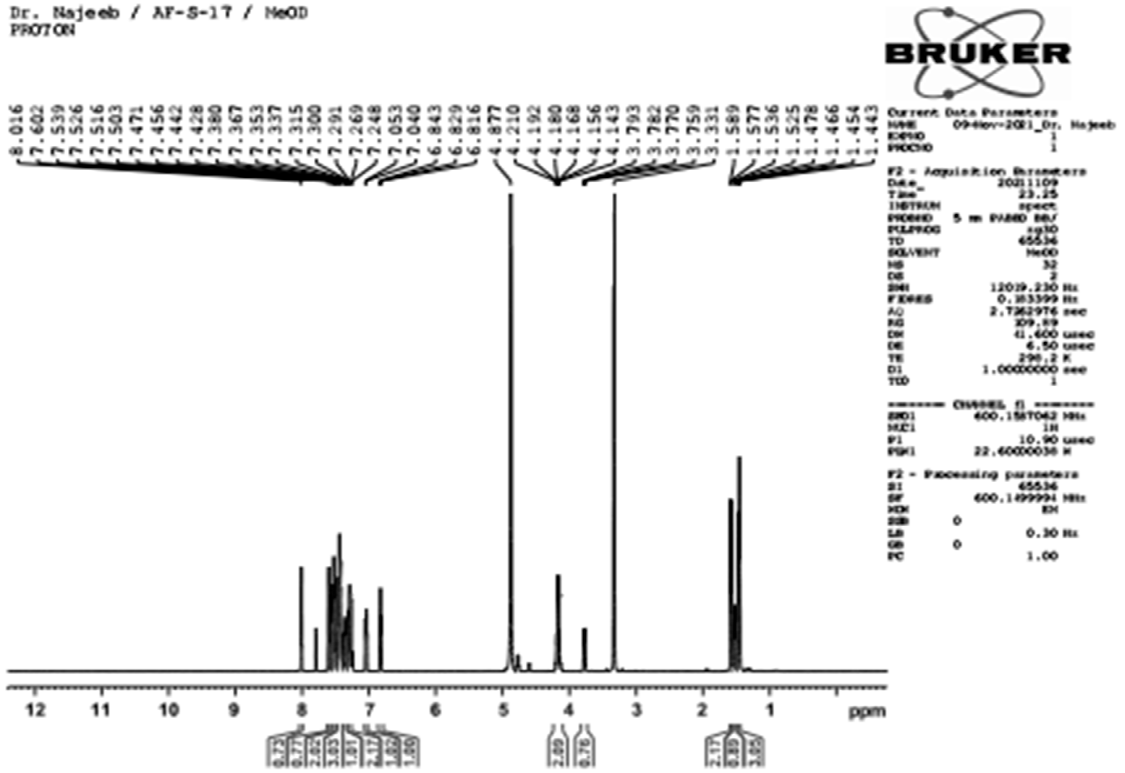

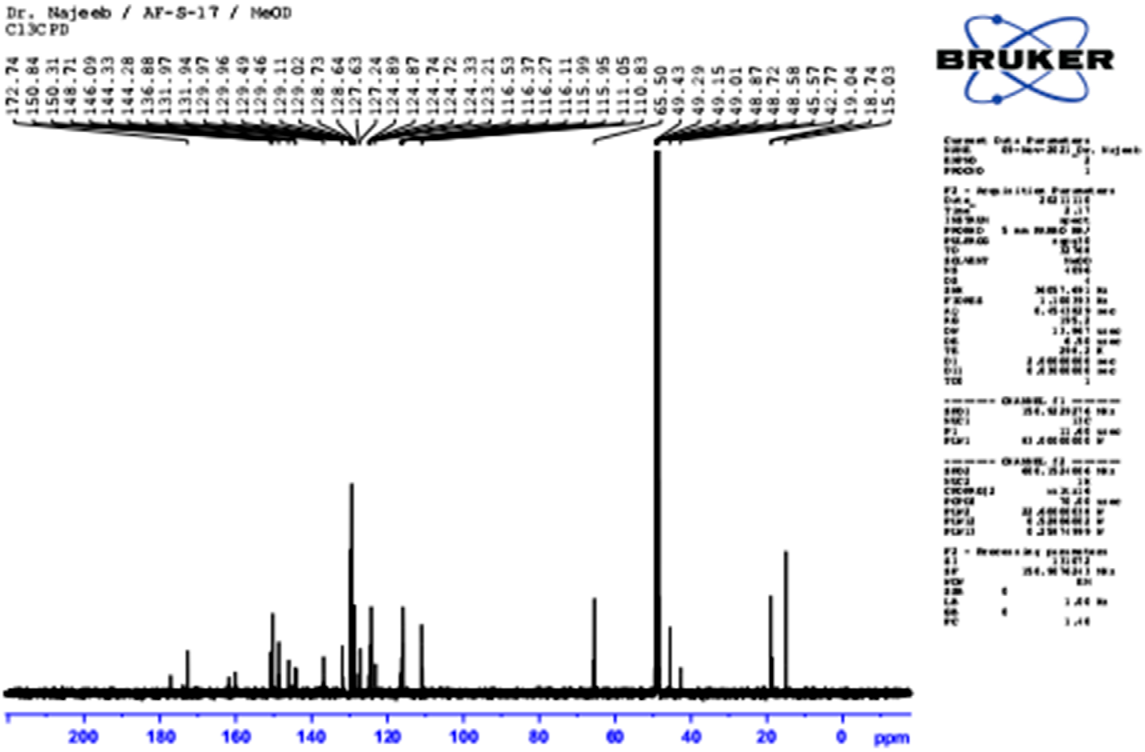

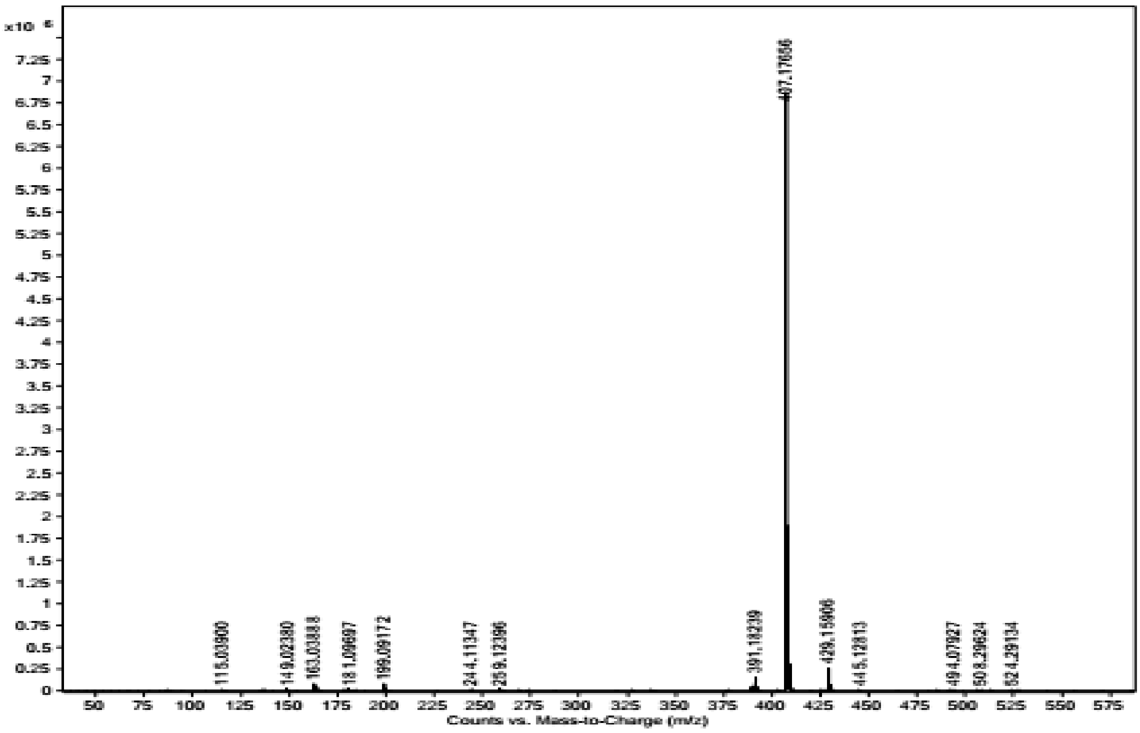

Figure S10: ^{1}H, ^{13}C NMR and HRMS (ESI^{+}) of compound **4j**

Dr. Najeeb / AF-S-18 / MeOD
PROTON

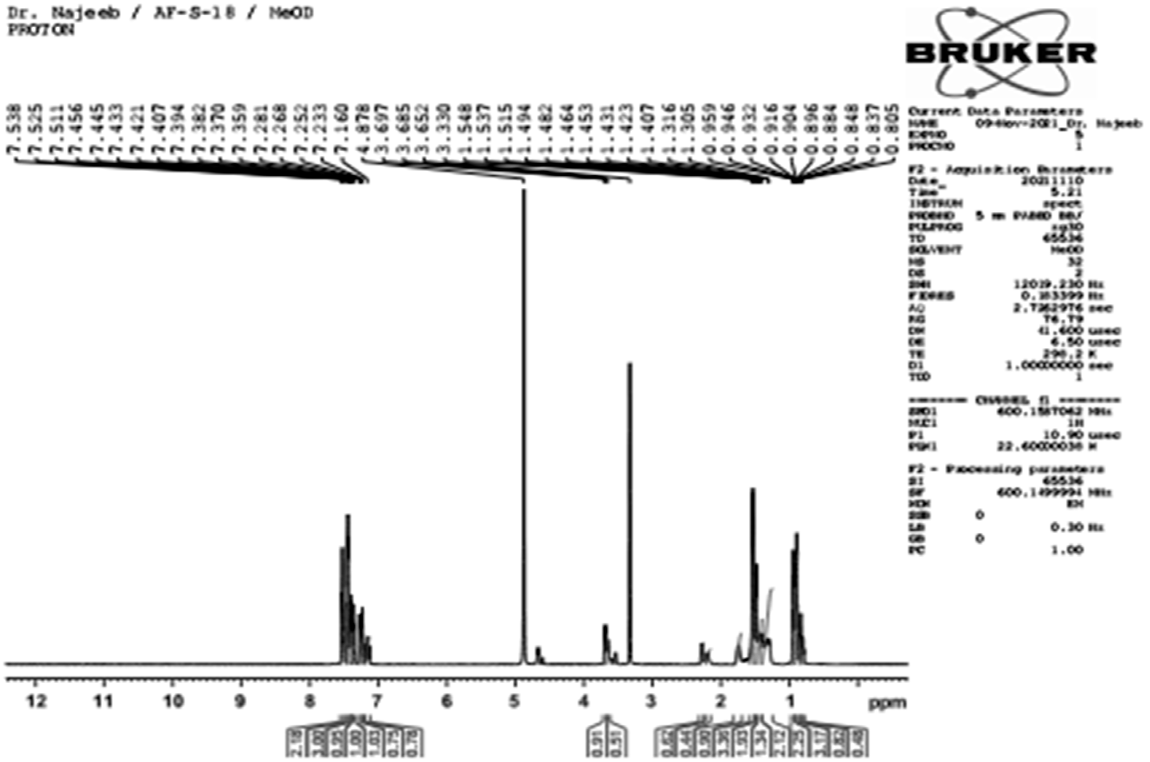

Dr. Najeeb / AF-S-18 / MeOD
C13CPD

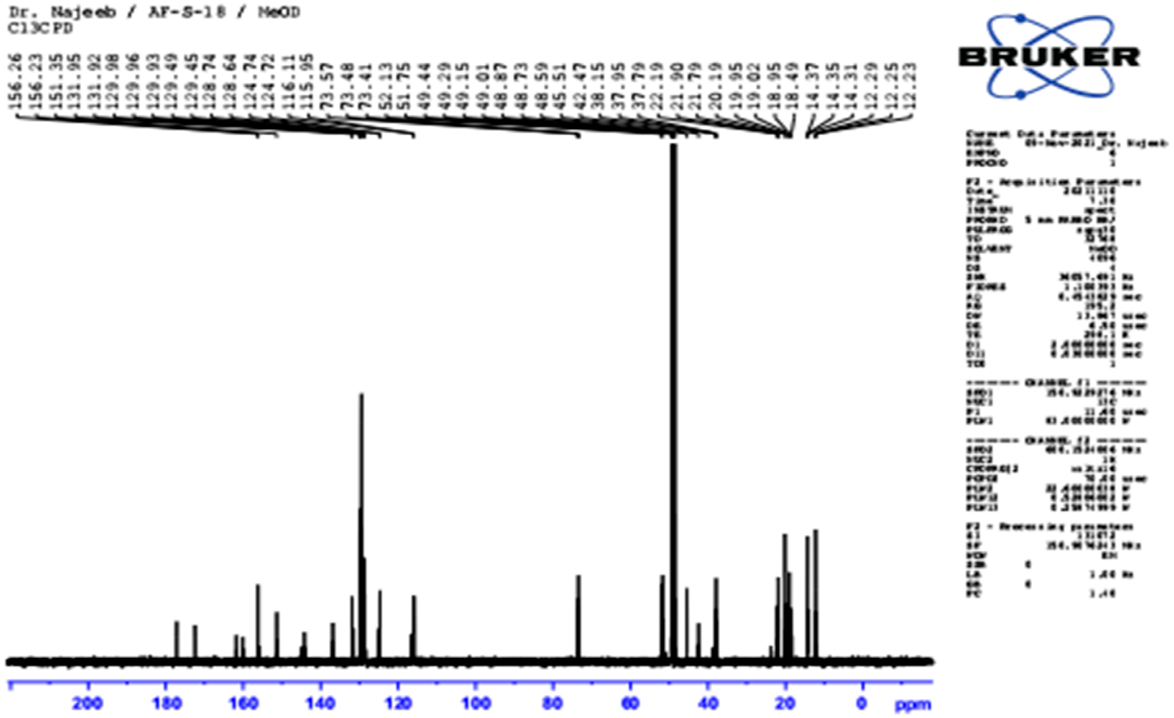

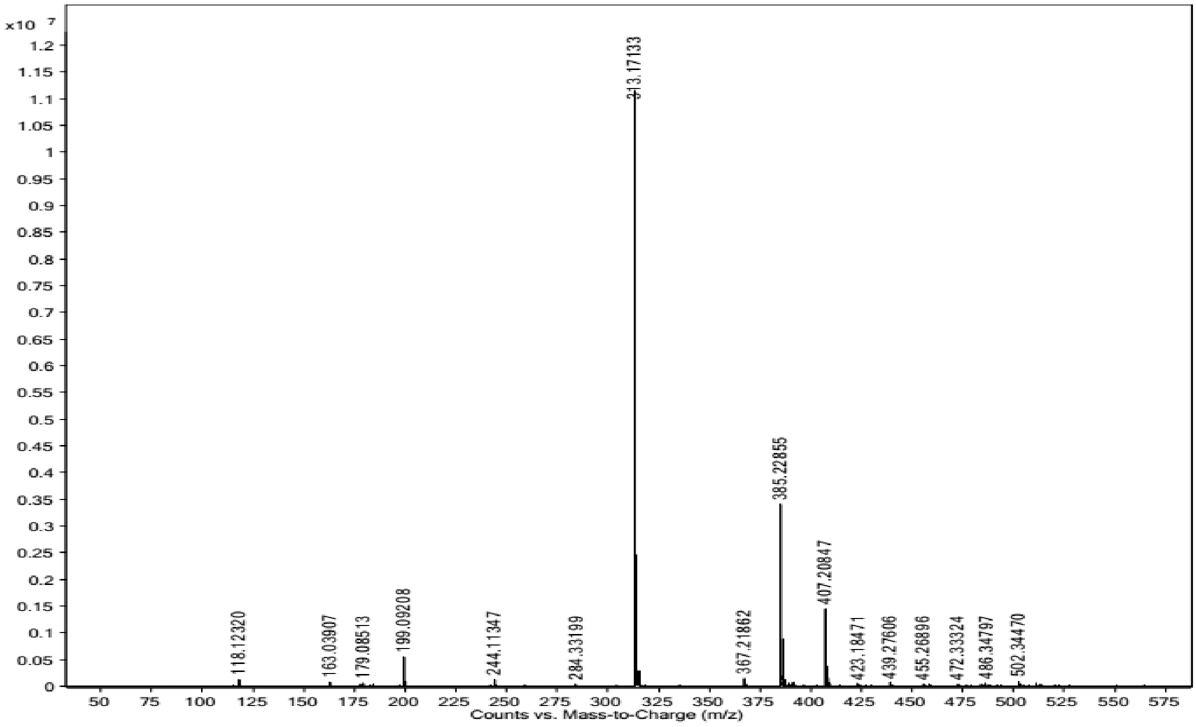

Figure S11: ^{1}H, ^{13}C NMR and HRMS (ESI^{+}) of compound **4k**

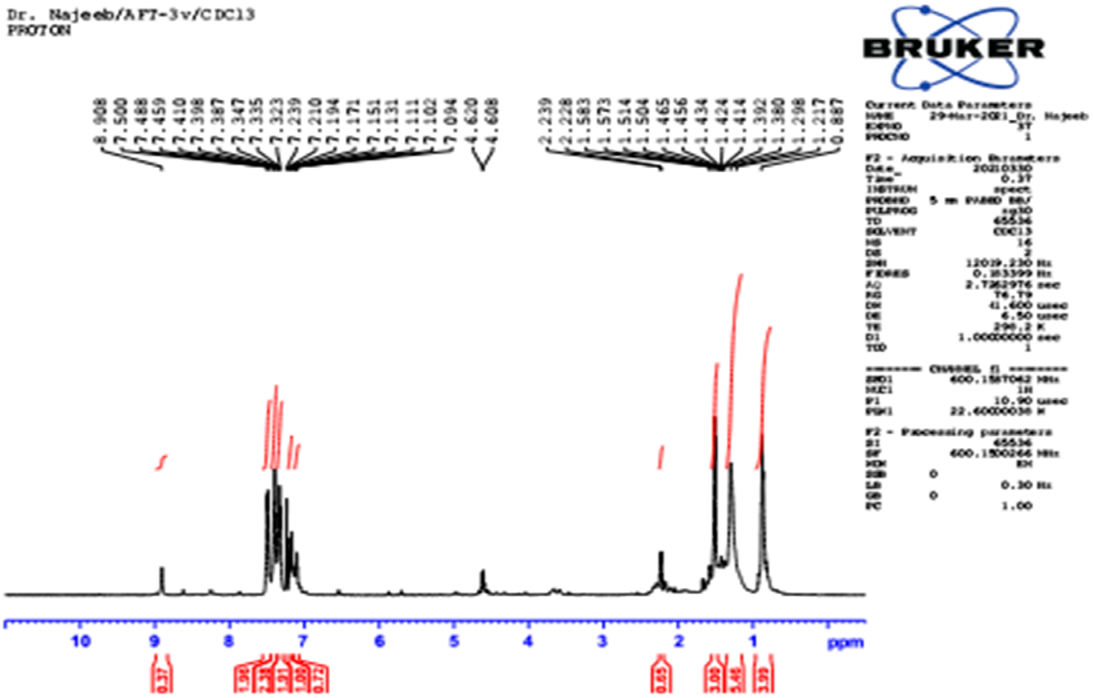

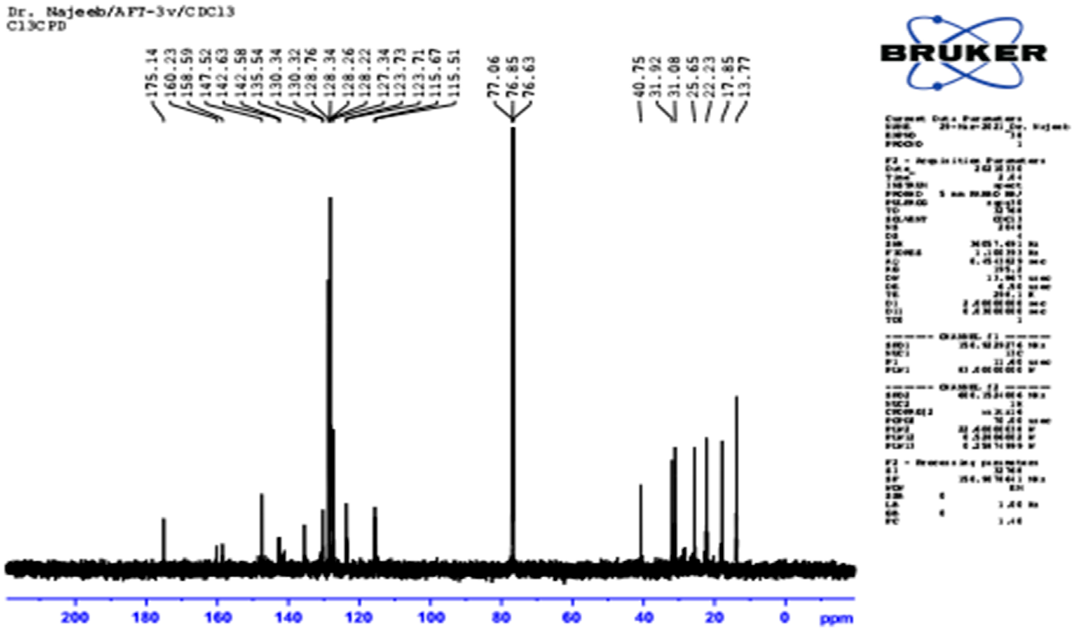

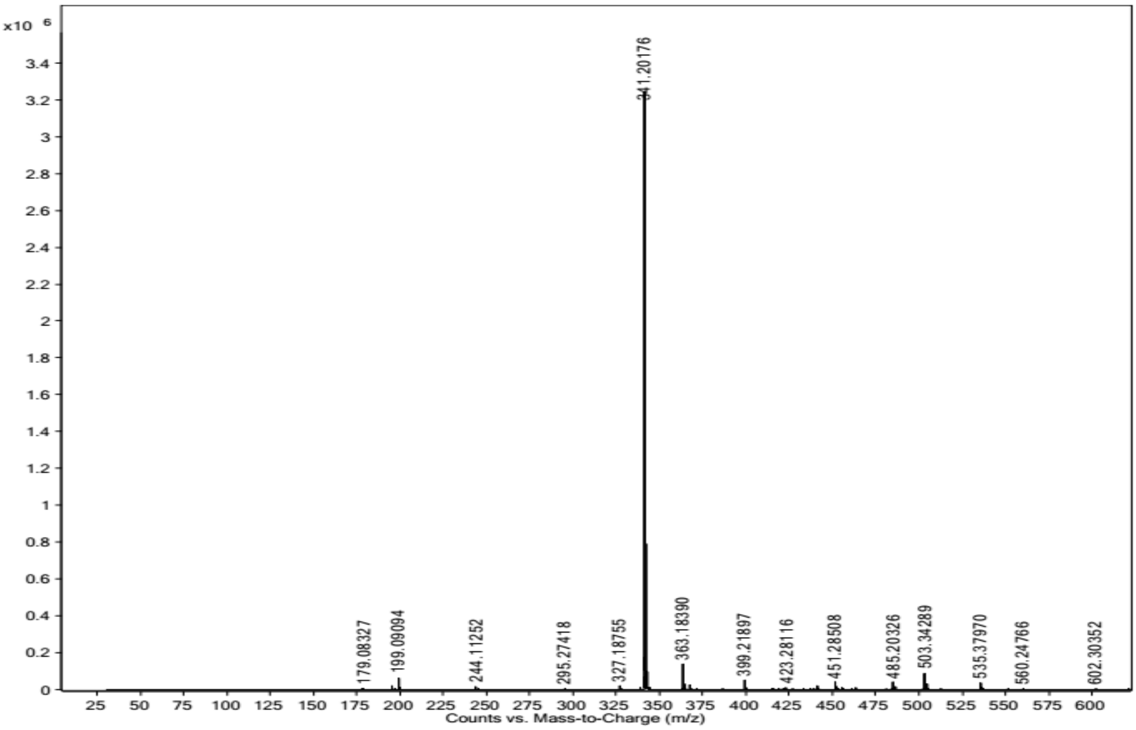

Figure S12: ^{1}H, ^{13}C NMR and HRMS (ESI^{+}) of compound **4l**

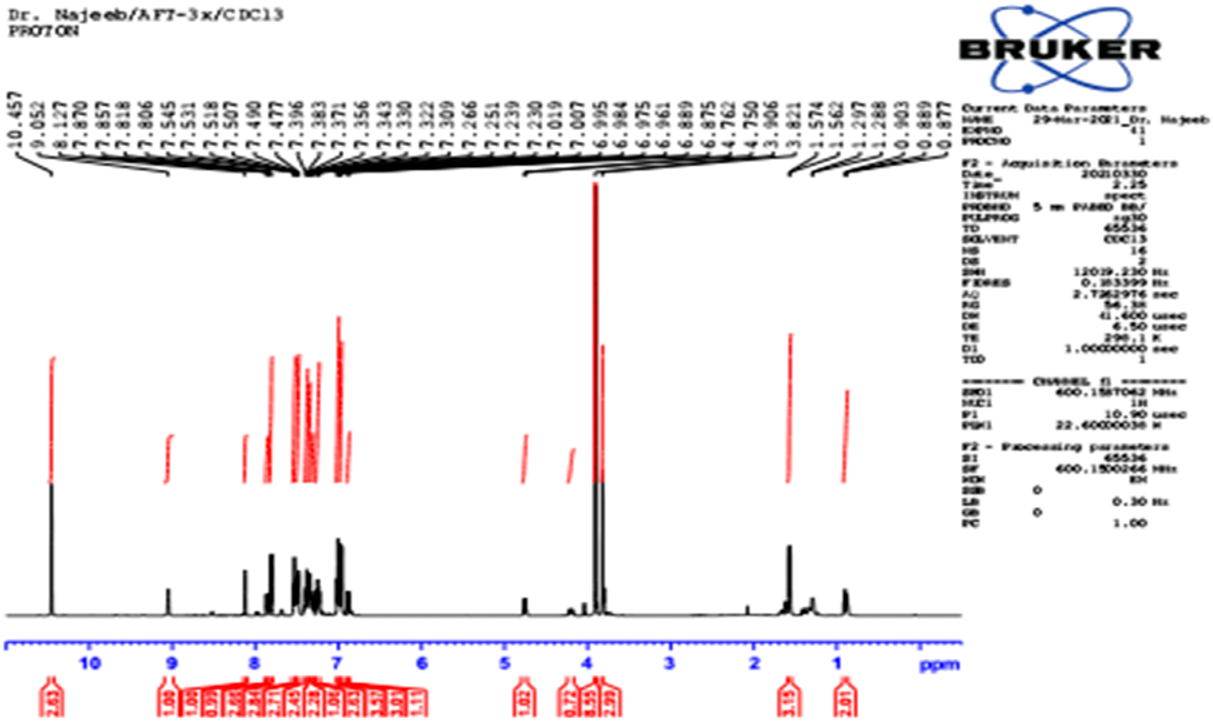
Dr. Najeeb/AF7-3x/CDCl3
PROTON
BRUKER
ppm

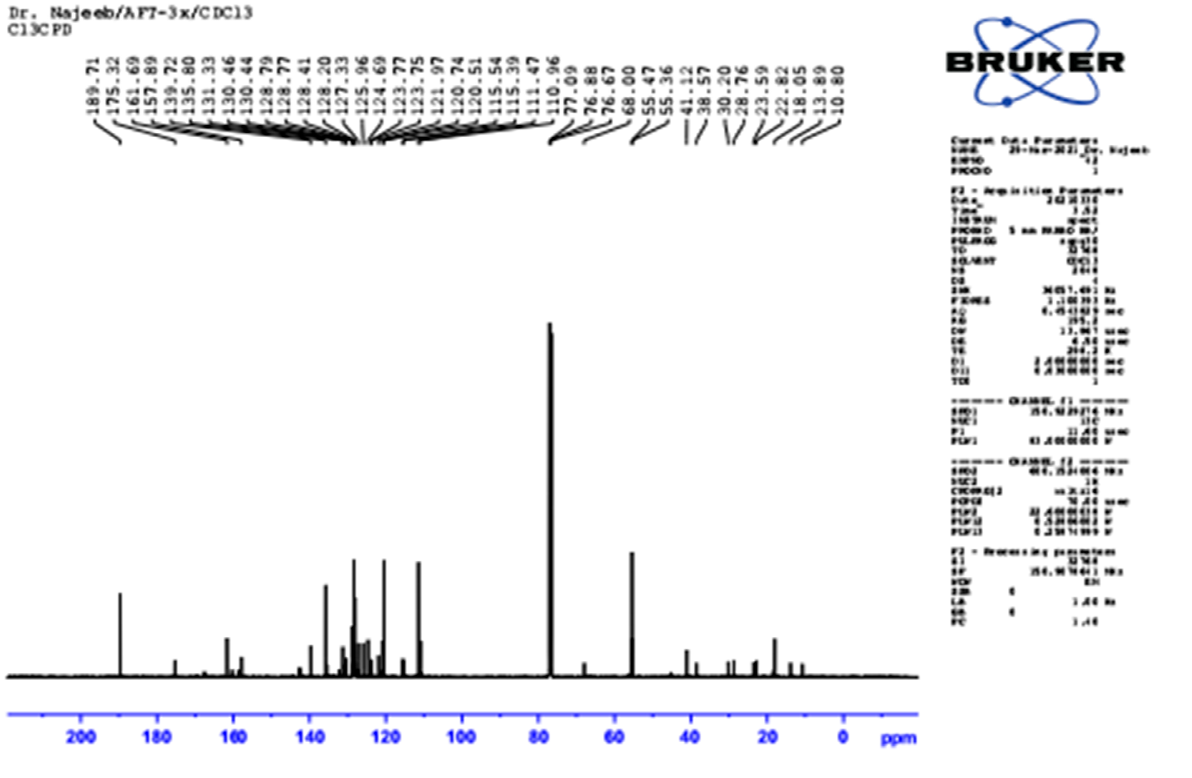
Dr. Najeeb/AF7-3x/CDCl3
C13CPD
BRUKER
ppm

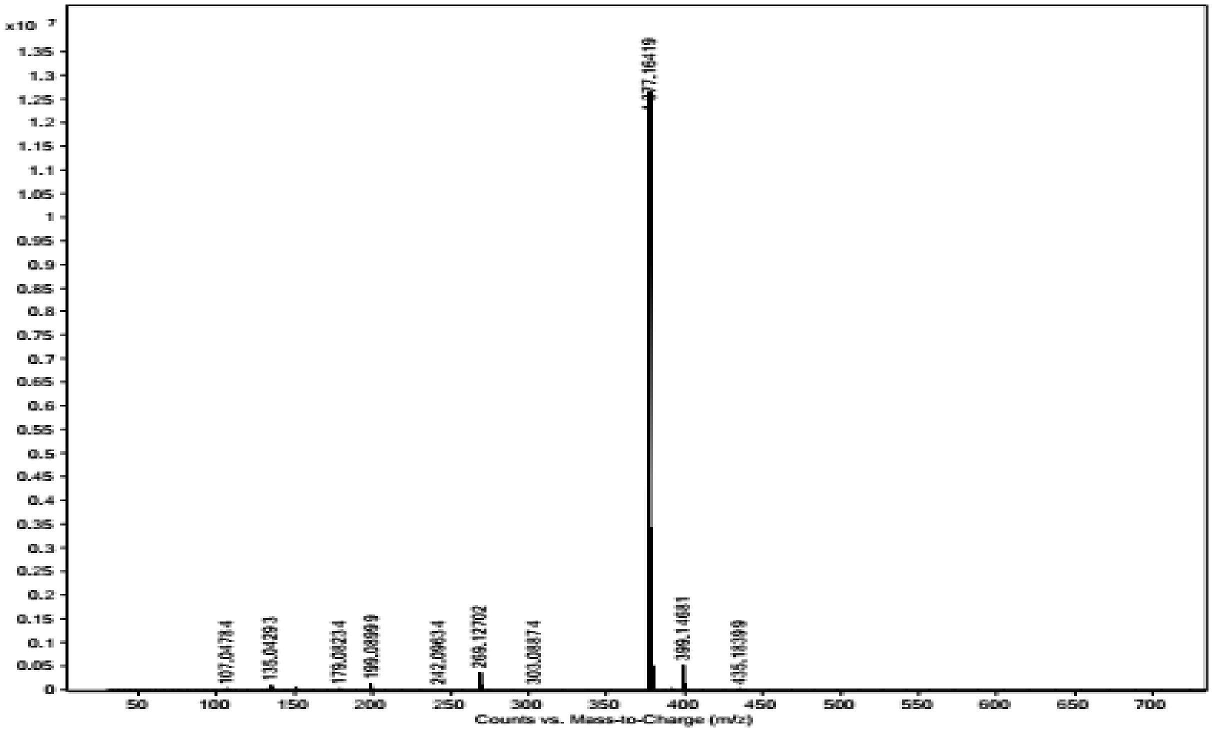

Figure S13: ^{1}H, ^{13}C NMR and HRMS (ESI^{+}) of compound **4m**

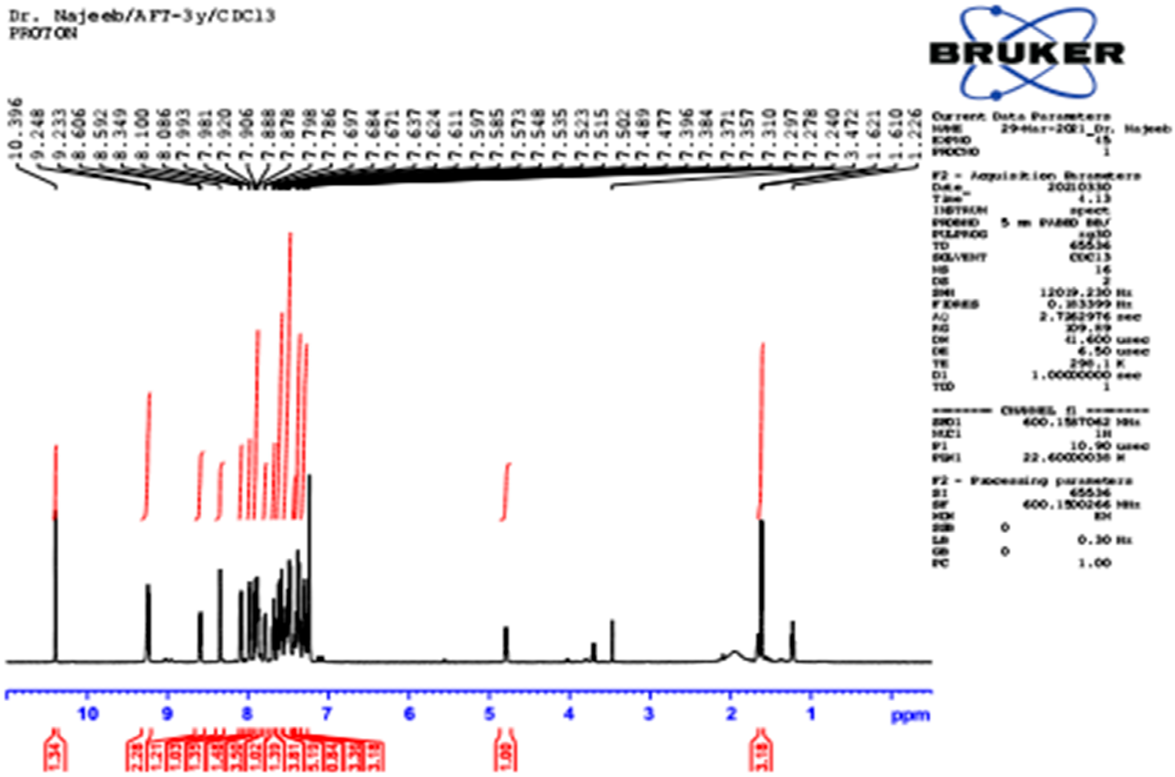

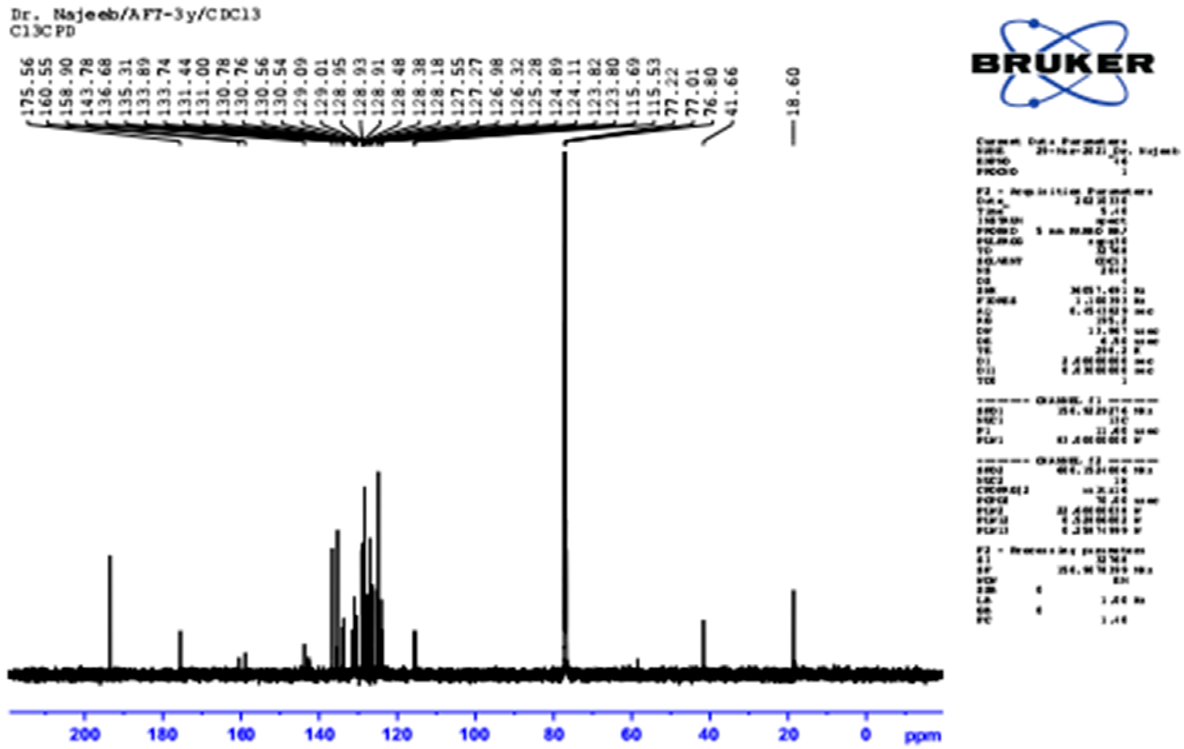

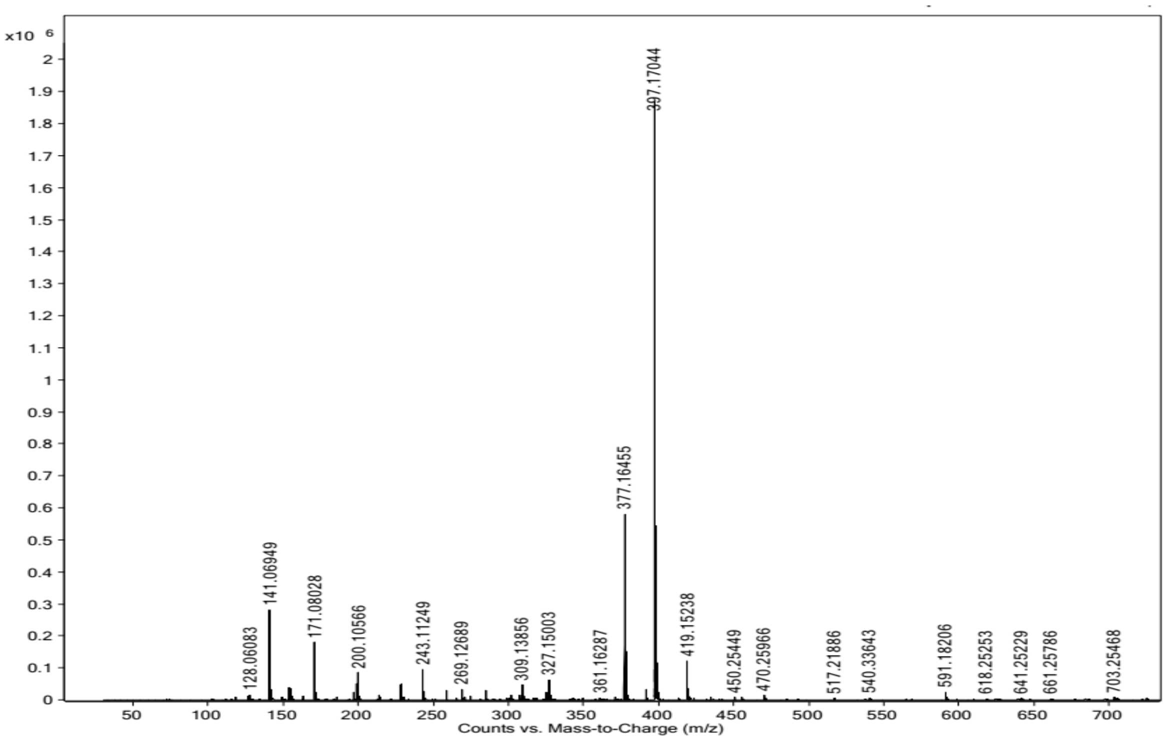

Figure S14: ^{1}H, ^{13}C NMR and HRMS (ESI^{+}) of compound **4n**

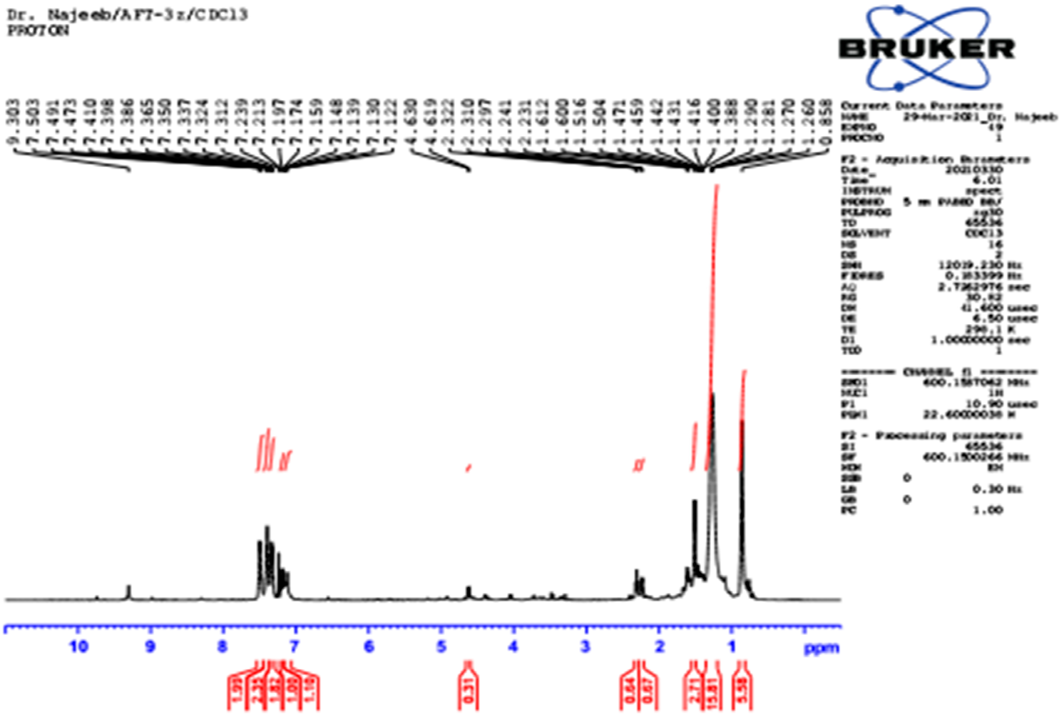
Dr. Najeeb/AF7-3z/CDCl3
PROTON
BRUKER
ppm

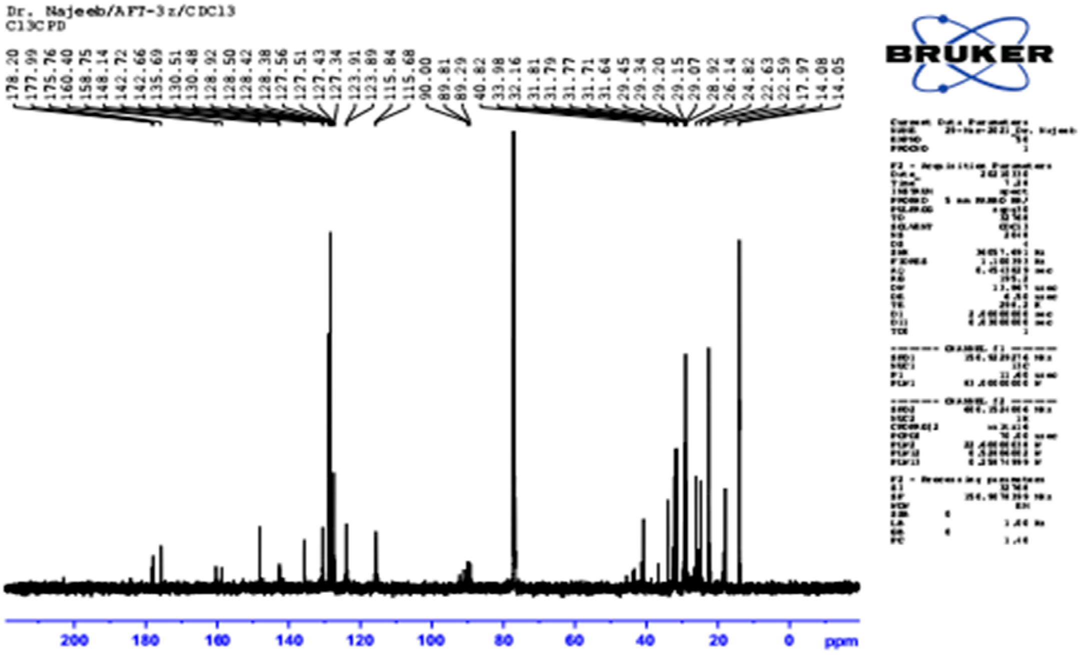
Dr. Najeeb/AF7-3z/CDCl3
C13CPD
BRUKER
ppm

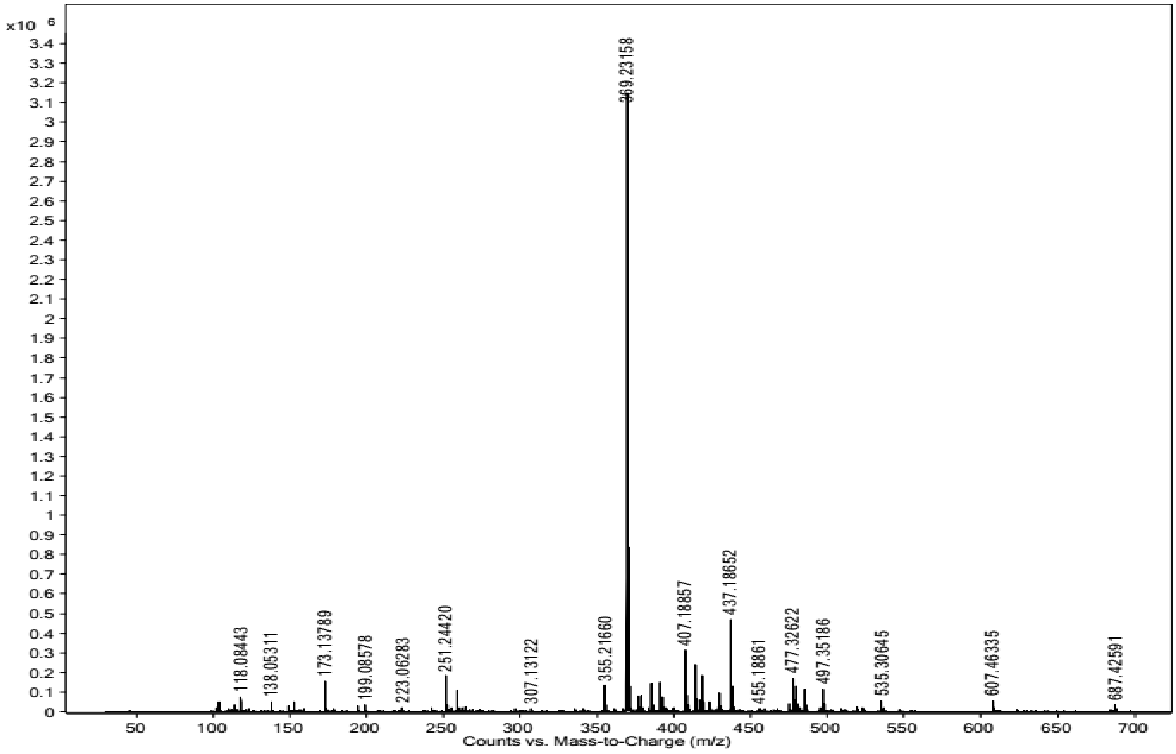

Figure S15: ^{1}H, ^{13}C NMR and HRMS (ESI^{+}) of compound **4o**

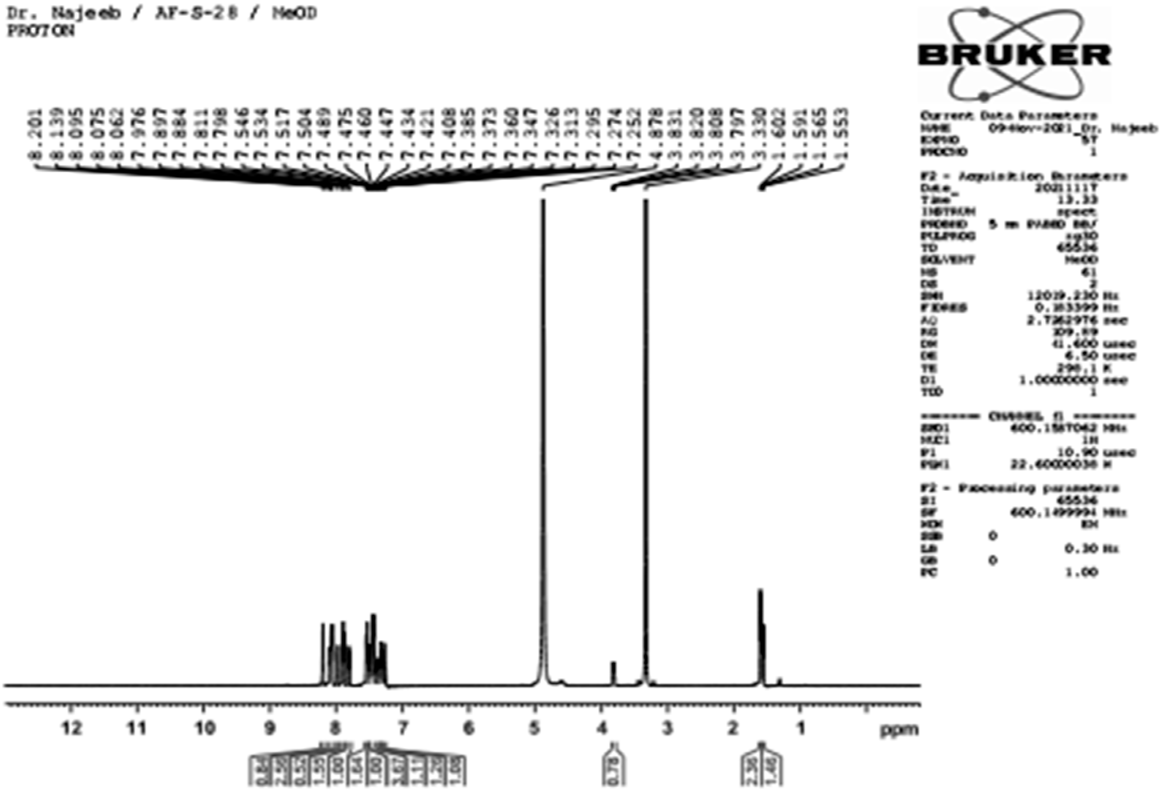

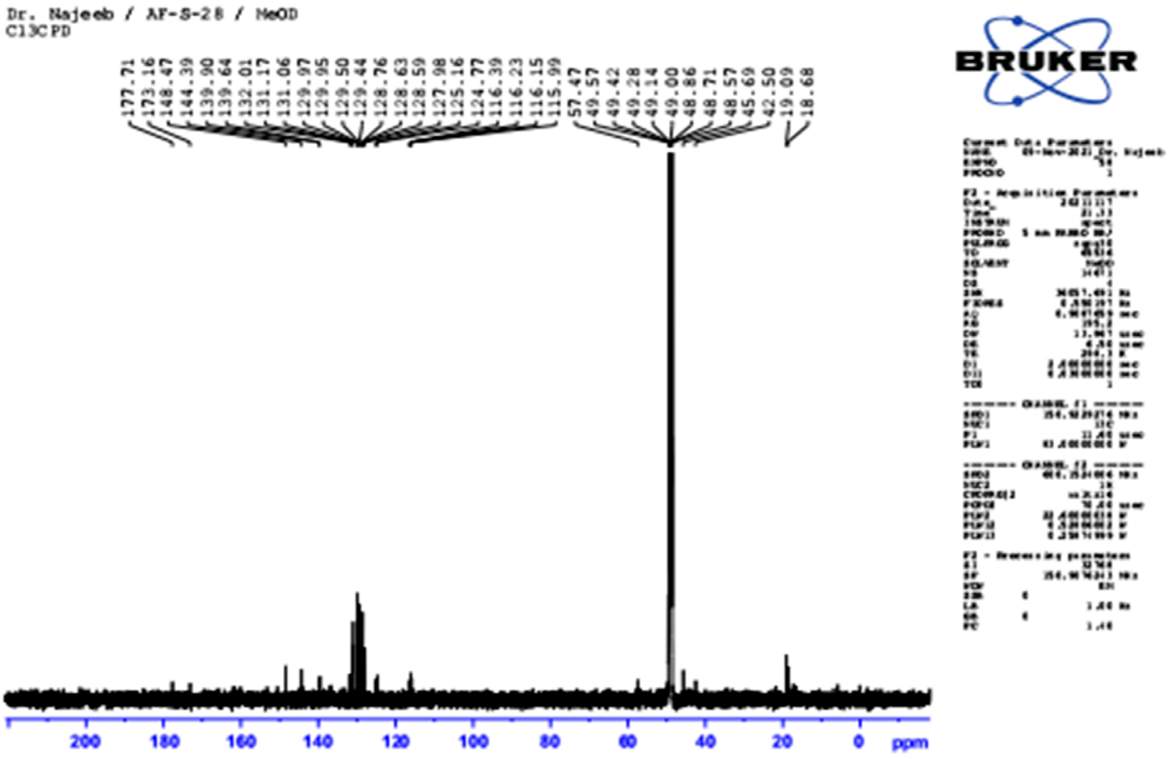

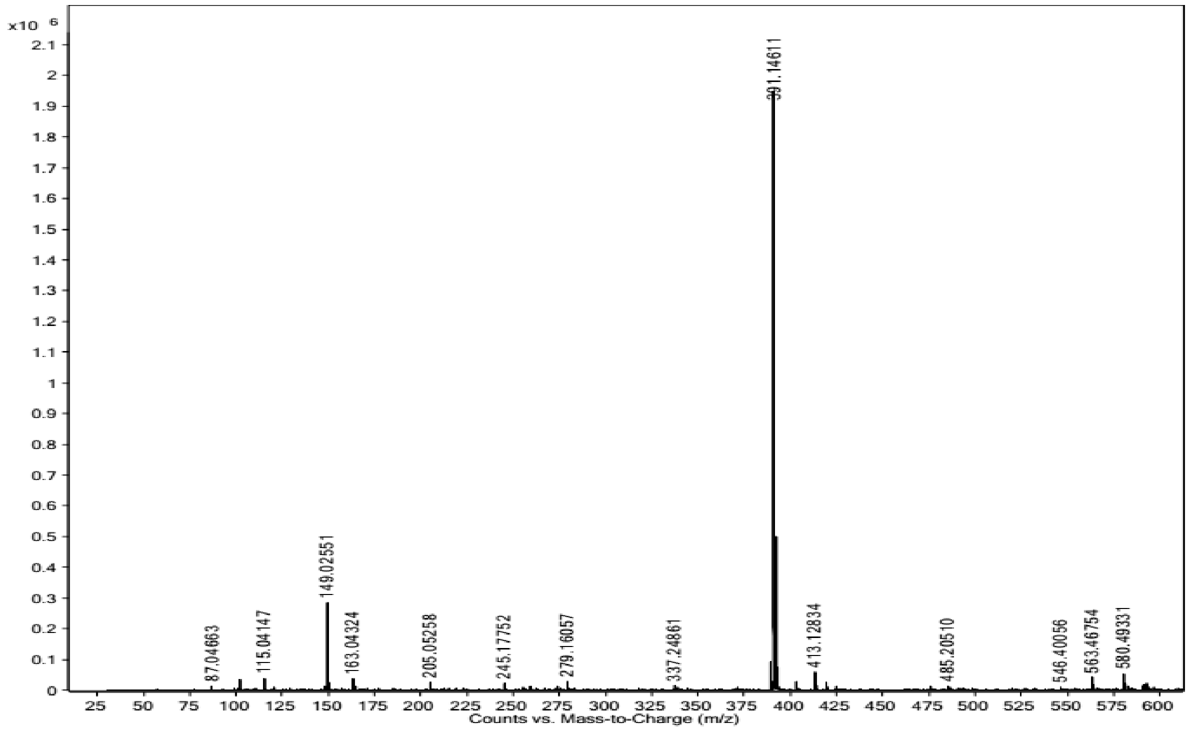

Figure S16: ^{1}H, ^{13}C NMR and HRMS (ESI^{+}) of compound **4p**

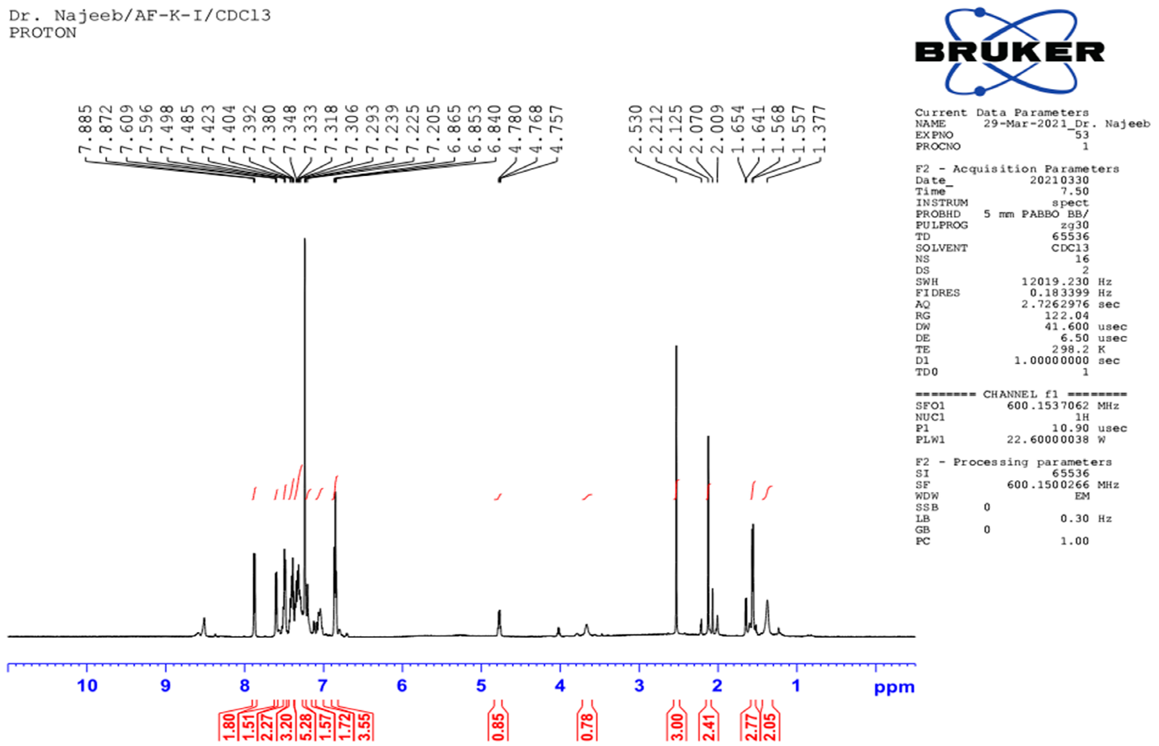
Dr. Najeeb/AF-K-I/CDCl3
PROTON
7.885 7.872 7.609 7.596 7.498 7.485 7.423 7.404 7.392 7.380 7.348 7.333 7.318 7.306 7.293 7.239 7.225 7.205 6.865 6.853 6.840 4.780 4.768 4.757
2.530 2.212 2.125 2.070 2.009 1.654 1.641 1.568 1.557 1.377
BRUKER
Current Data Parameters
NAME 29-Mar-2021_Dr. Najeeb
EXPNO 53
PROCNO 1
F2 - Acquisition Parameters
Date_ 20210330
Time 7.50
INSTRUM spect
PROBHD 5 mm PABBO BB/
PULPROG zg30
TD 65536
SOLVENT CDCl3
NS 16
DS 2
SWH 12019.230 Hz
FIDRES 0.183399 Hz
AQ 2.7262976 sec
RG 122.04
DW 41.600 usec
DE 6.50 usec
TE 298.2 K
D1 1.00000000 sec
TD0 1
======== CHANNEL f1 ========
SFO1 600.1537062 MHz
NUC1 1H
P1 10.90 usec
PLW1 22.60000038 W
F2 - Processing parameters
SI 65536
SF 600.1500266 MHz
WDW EM
SSB 0
LB 0.30 Hz
GB 0
PC 1.00
10 9 8 7 6 5 4 3 2 1 ppm
1.80 1.51 2.27 3.20 5.28 1.57 1.72 3.55 0.85 0.78 3.00 2.41 2.77 2.05

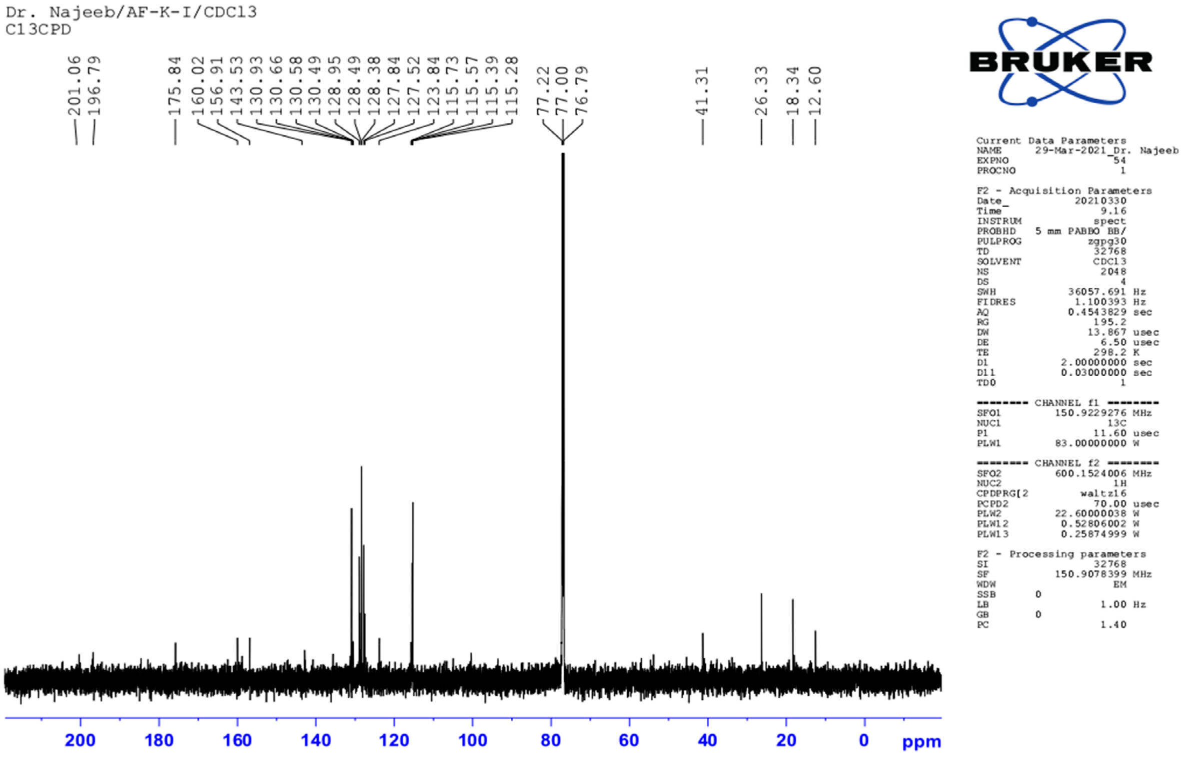
Dr. Najeeb/AF-K-I/CDCl3
C13CPD
201.06 196.79 175.84 160.02 156.91 143.53 130.93 130.66 130.58 130.49 128.95 128.49 128.38 127.84 127.52 123.84 115.73 115.57 115.39 115.28 77.22 77.00 76.79 41.31 26.33 18.34 12.60
BRUKER
Current Data Parameters
NAME 29-Mar-2021_Dr. Najeeb
EXPNO 54
PROCNO 1
F2 - Acquisition Parameters
Date_ 20210330
Time 9.16
INSTRUM spect
PROBHD 5 mm PABBO BB/
PULPROG zgpg30
TD 32768
SOLVENT CDCl3
NS 2048
DS 4
SWH 36057.691 Hz
FIDRES 1.100393 Hz
AQ 0.4543829 sec
RG 195.2
DW 13.867 usec
DE 6.50 usec
TE 298.2 K
D1 2.00000000 sec
D11 0.03000000 sec
TD0 1
======== CHANNEL f1 ========
SFO1 150.9229276 MHz
NUC1 13C
P1 11.60 usec
PLW1 83.00000000 W
======== CHANNEL f2 ========
SFO2 600.1524006 MHz
NUC2 1H
CPDPRG[2 waltz16
PCPD2 70.00 usec
PLW2 22.60000038 W
PLW12 0.52806002 W
PLW13 0.25874999 W
F2 - Processing parameters
SI 32768
SF 150.9078399 MHz
WDW EM
SSB 0
LB 1.00 Hz
GB 0
PC 1.40
200 180 160 140 120 100 80 60 40 20 0 ppm

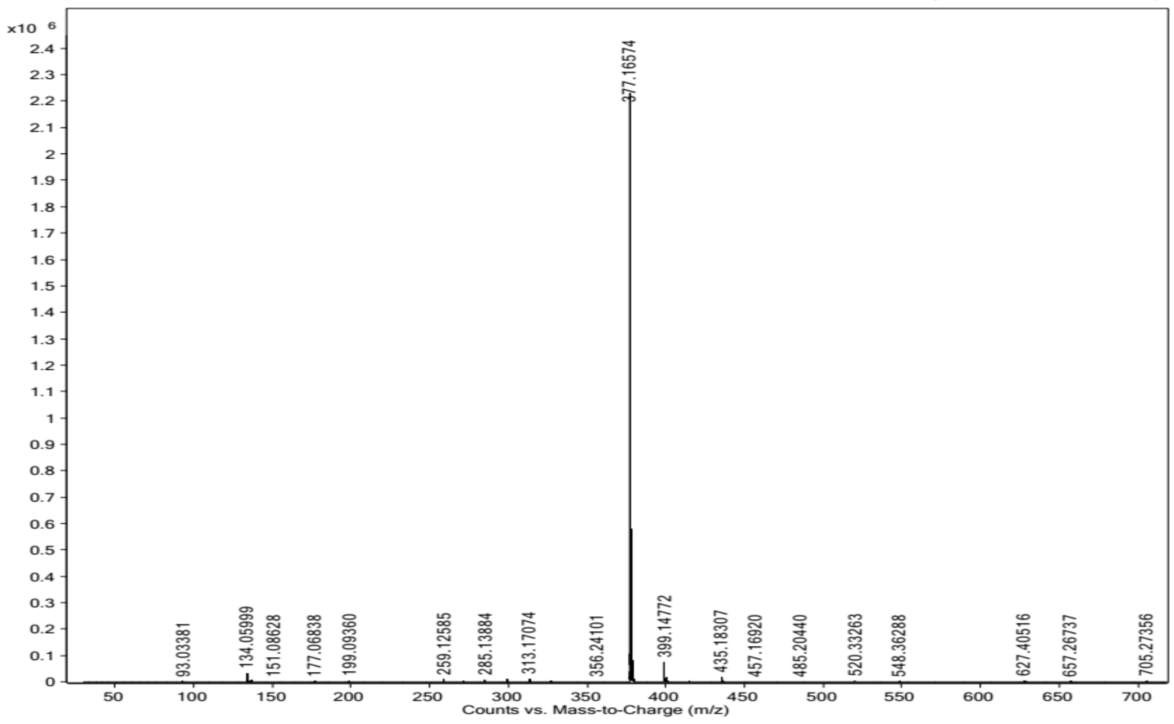

Figure S17. ^{1}H, ^{13}C NMR and HRMS (ESI^{+}) of compound **5a**

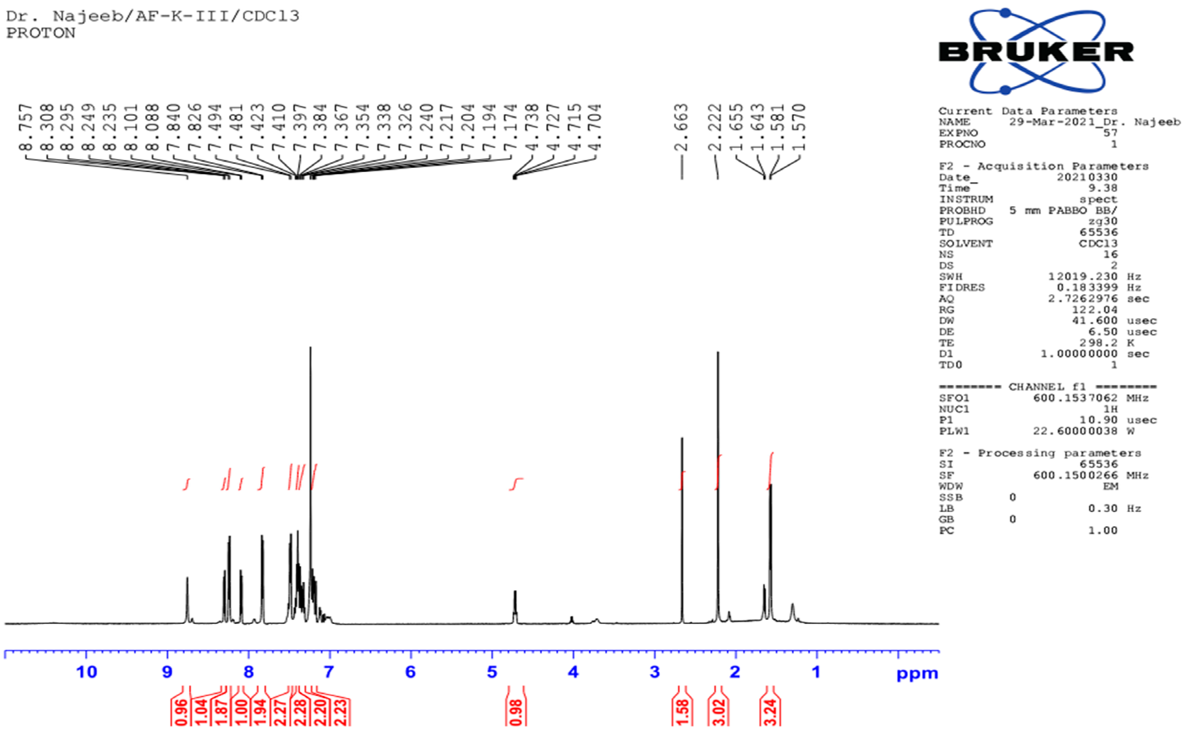

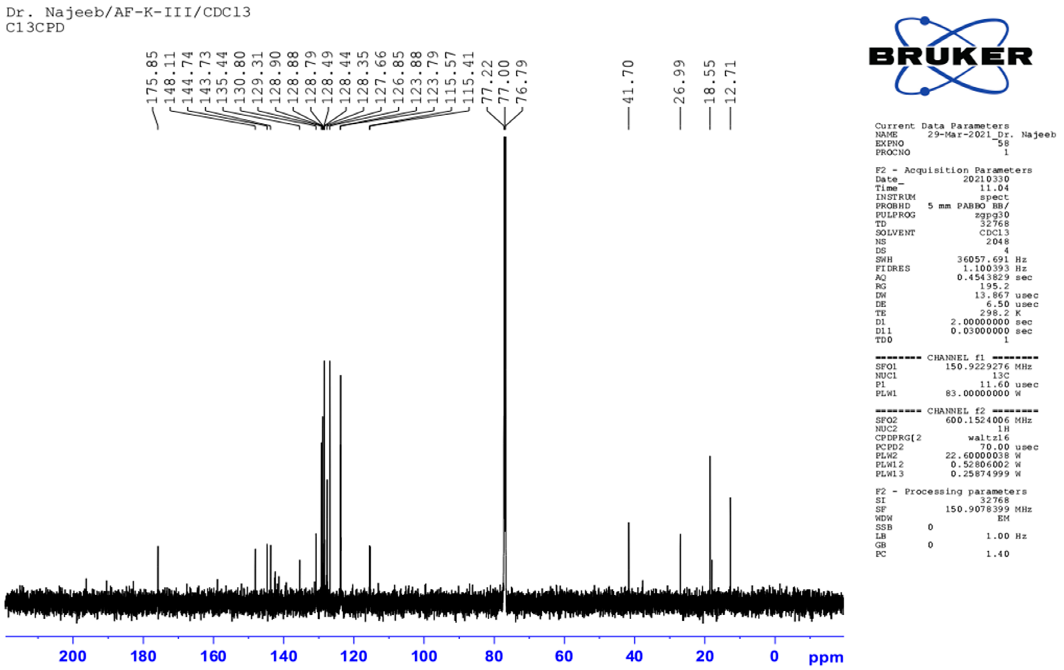

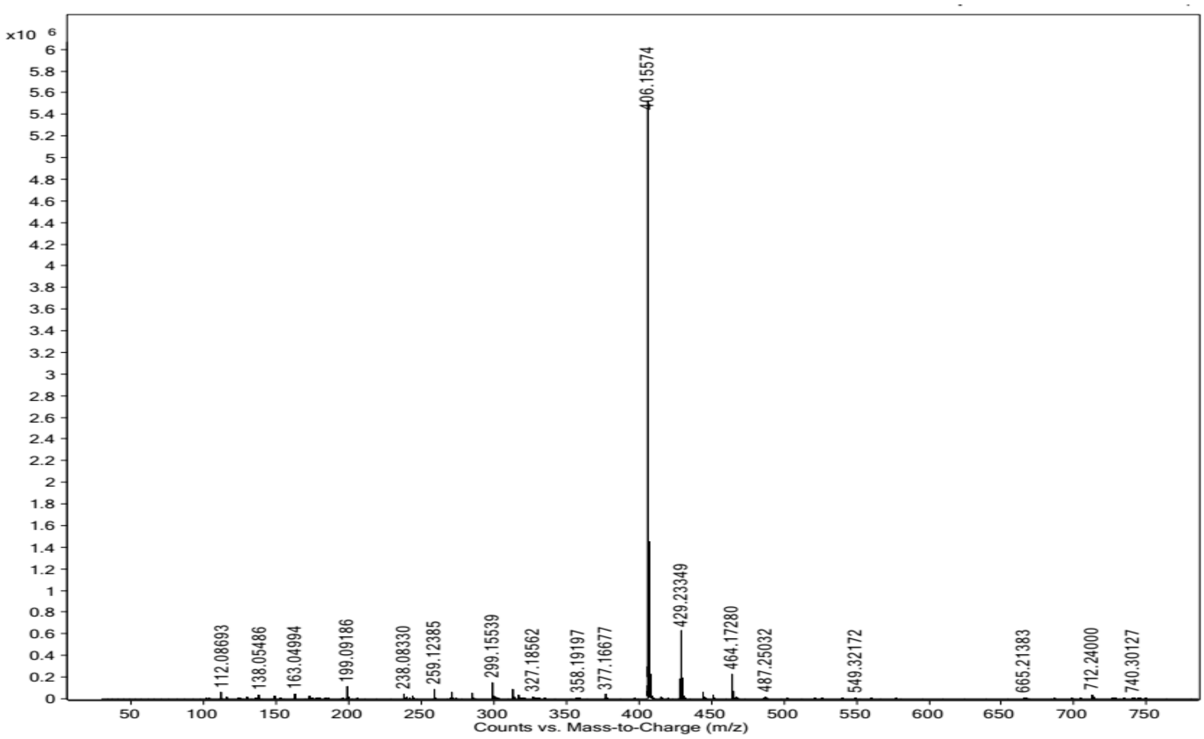

Figure S18: ^{1}H, ^{13}C NMR and HRMS (ESI^{+}) of compound **5b**

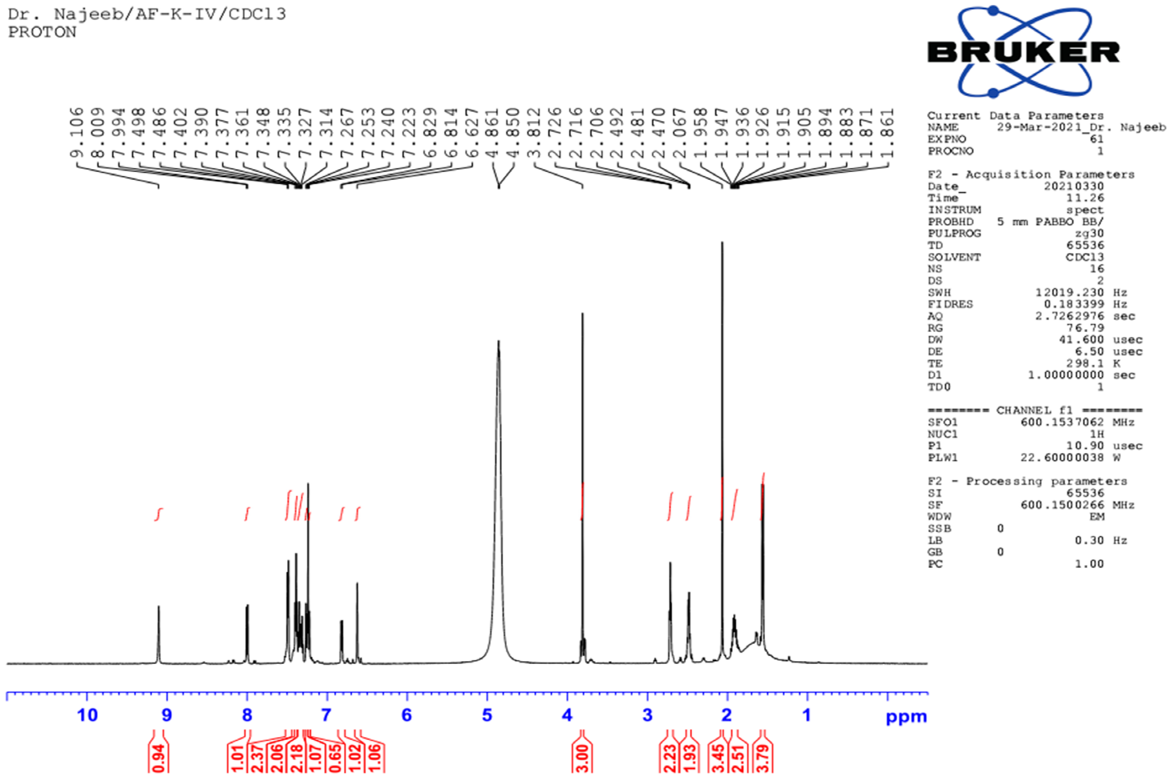

Dr. Najeeb/AF-K-IV/CDCl3
PROTON
9.106 8.009 7.994 7.498 7.486 7.402 7.390 7.377 7.361 7.348 7.335 7.327 7.314 7.267 7.253 7.240 7.223 6.829 6.814 6.627 4.861 4.850 3.812 2.726 2.716 2.706 2.492 2.481 2.470 2.067 1.958 1.947 1.936 1.926 1.915 1.905 1.894 1.883 1.871 1.861
BRUKER
Current Data Parameters
NAME 29-Mar-2021_Dr. Najeeb
EXPNO 61
PROCNO 1
F2 - Acquisition Parameters
Date_ 20210330
Time 11.26
INSTRUM spect
PROBHD 5 mm PABBO BB/
PULPROG zg30
TD 65536
SOLVENT CDCl3
NS 16
DS 2
SWH 12019.230 Hz
FIDRES 0.183399 Hz
AQ 2.7262976 sec
RG 76.79
DW 41.600 usec
DE 6.50 usec
TE 298.1 K
D1 1.00000000 sec
TD0 1
======== CHANNEL f1 ========
SFO1 600.1537062 MHz
NUC1 1H
P1 10.90 usec
PLW1 22.60000038 W
F2 - Processing parameters
SI 65536
SF 600.1500266 MHz
WDW EM
SSB 0
LB 0.30 Hz
GB 0
PC 1.00
10 9 8 7 6 5 4 3 2 1 ppm
0.94 1.01 2.37 2.06 2.18 1.07 0.65 1.02 1.06 3.00 2.23 1.93 3.45 2.51 3.79

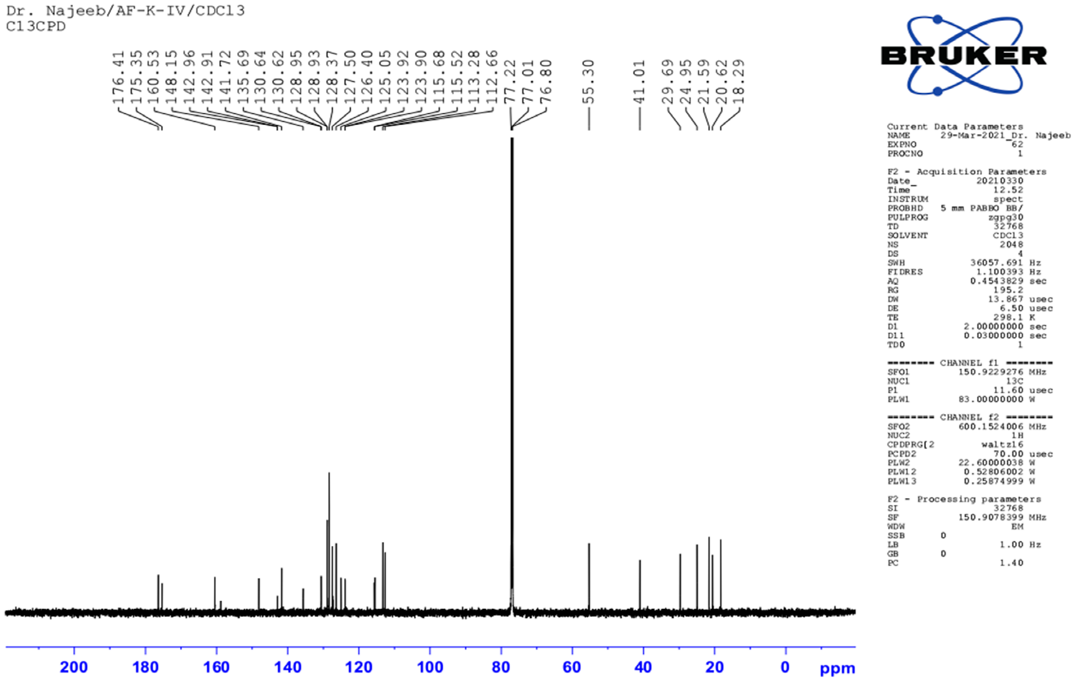

Dr. Najeeb/AF-K-IV/CDCl3
C13CPD
176.41 175.35 160.53 148.15 142.96 142.91 141.72 135.69 130.64 130.62 128.95 128.93 128.37 127.50 126.40 125.05 123.92 123.90 115.68 115.52 113.28 112.66 77.22 77.01 76.80 55.30 41.01 29.69 24.95 21.59 20.62 18.29
BRUKER
Current Data Parameters
NAME 29-Mar-2021_Dr. Najeeb
EXPNO 62
PROCNO 1
F2 - Acquisition Parameters
Date_ 20210330
Time 12.52
INSTRUM spect
PROBHD 5 mm PABBO BB/
PULPROG zgpg30
TD 32768
SOLVENT CDCl3
NS 2048
DS 4
SWH 36057.691 Hz
FIDRES 1.100393 Hz
AQ 0.4543829 sec
RG 195.2
DW 13.867 usec
DE 6.50 usec
TE 298.1 K
D1 2.00000000 sec
D11 0.03000000 sec
TD0 1
======== CHANNEL f1 ========
SFO1 150.9229276 MHz
NUC1 13C
P1 11.60 usec
PLW1 83.00000000 W
======== CHANNEL f2 ========
SFO2 600.1524006 MHz
NUC2 1H
CPDPRG[2 waltz16
PCPD2 70.00 usec
PLW2 22.60000038 W
PLW12 0.52806002 W
PLW13 0.25874999 W
F2 - Processing parameters
SI 32768
SF 150.9078399 MHz
WDW EM
SSB 0
LB 1.00 Hz
GB 0
PC 1.40
200 180 160 140 120 100 80 60 40 20 0 ppm

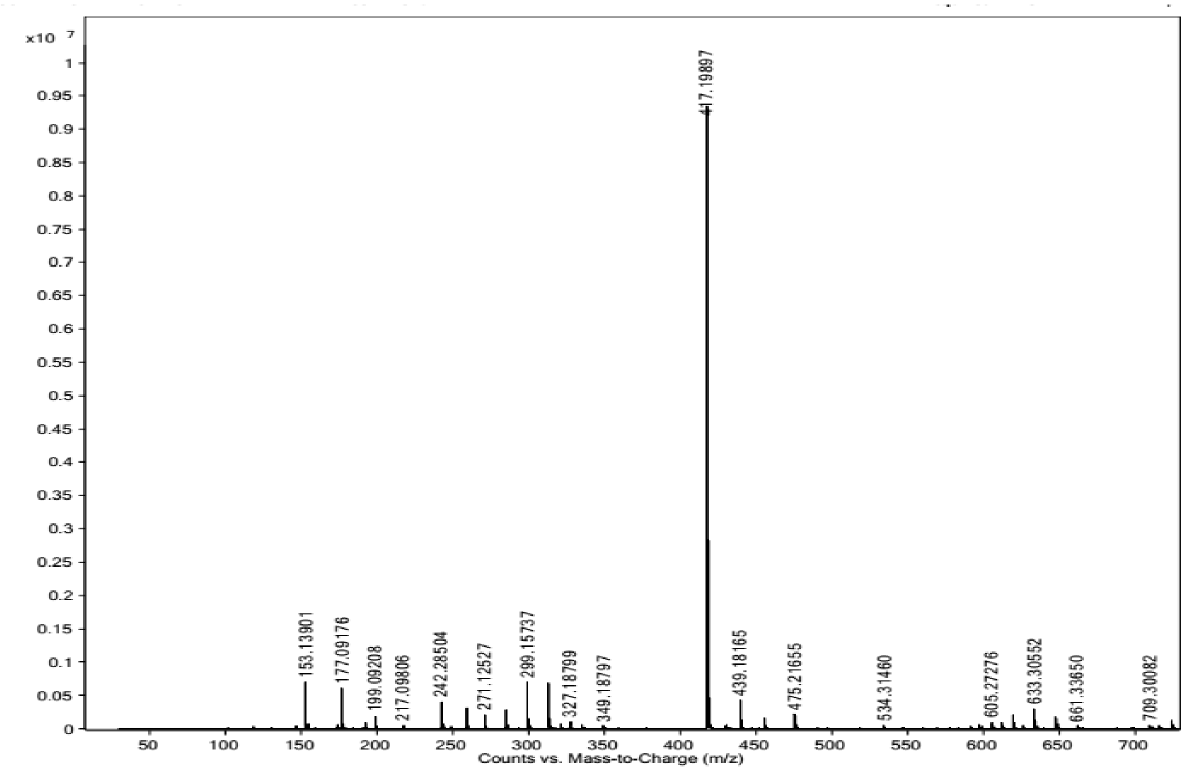

Figure S19: ^{1}H, ^{13}C NMR and HRMS (ESI^{+}) of compound **5c**

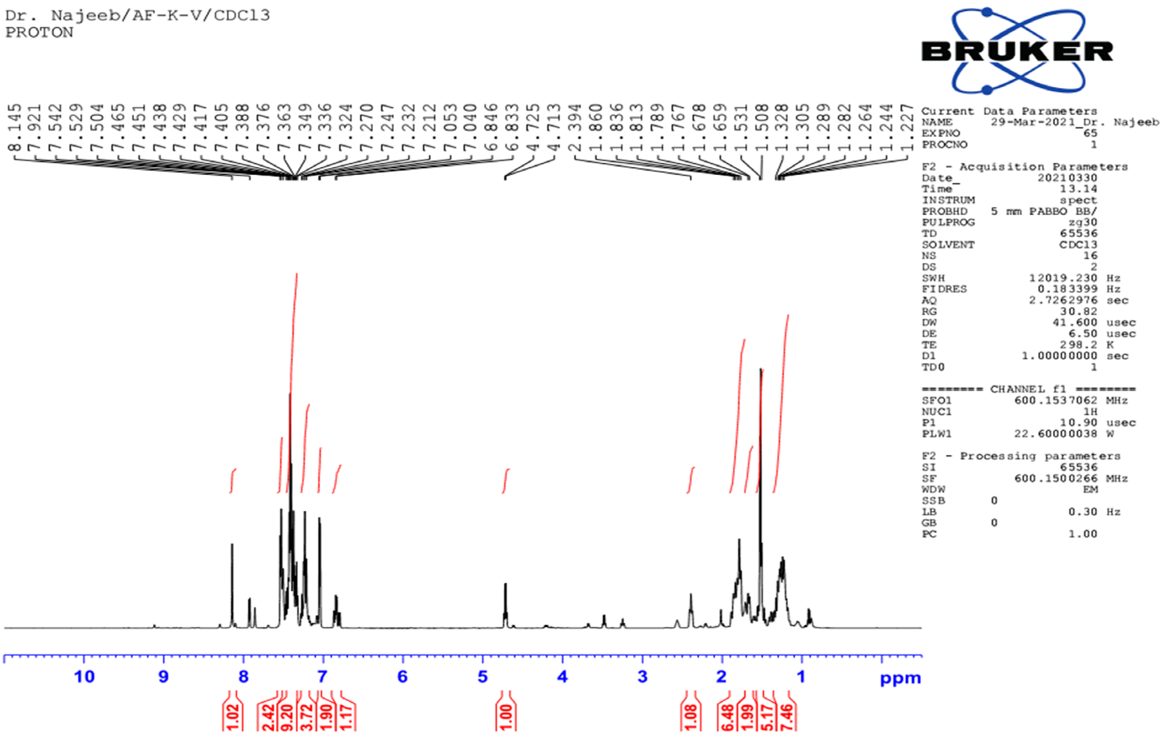

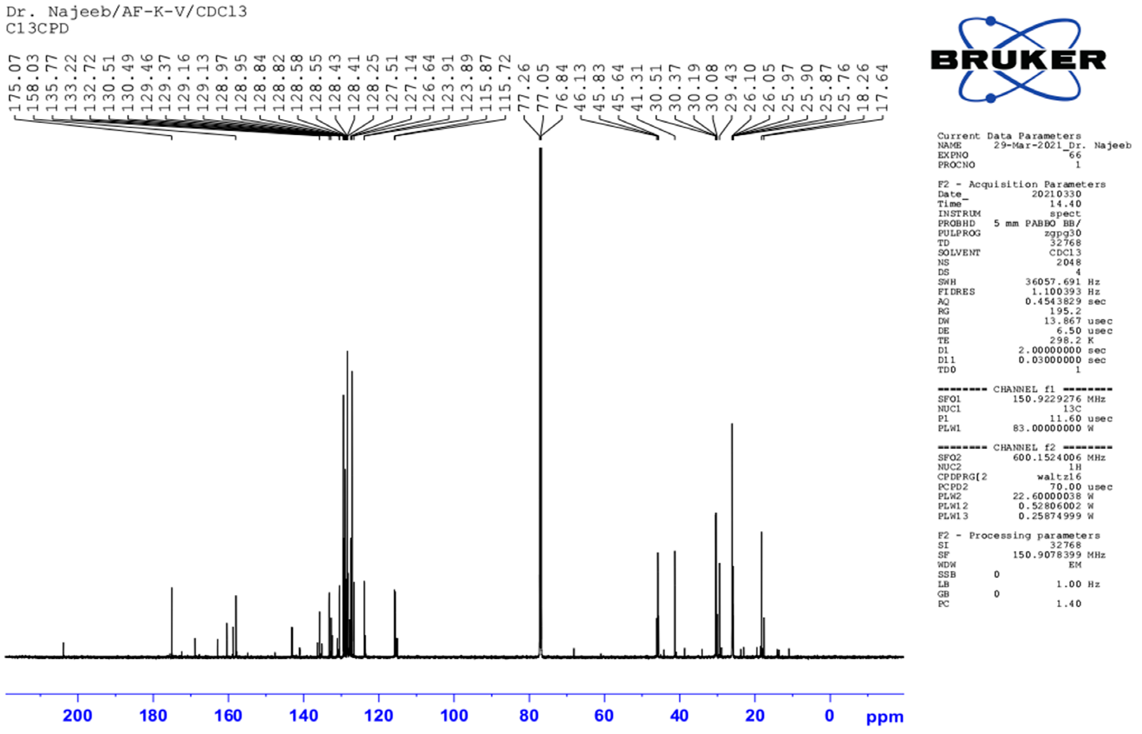

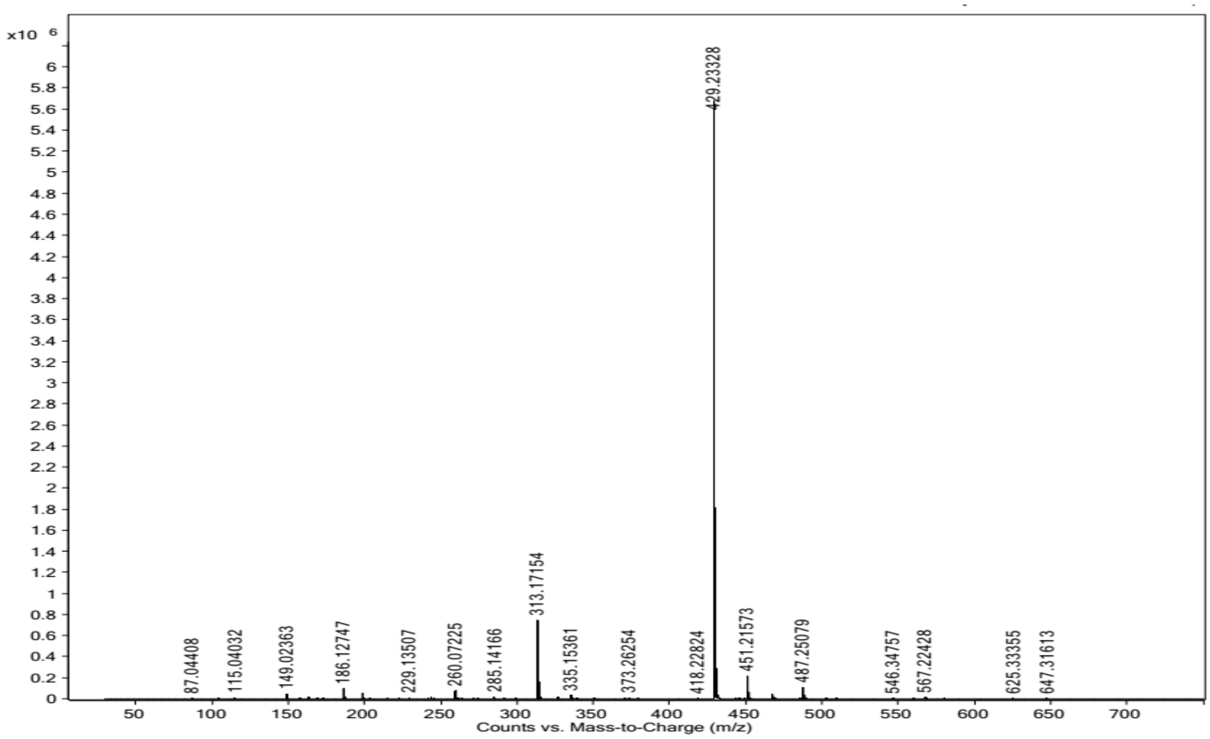

Figure S20: ^{1}H, ^{13}C NMR and HRMS (ESI$^+$) of compound **5d**

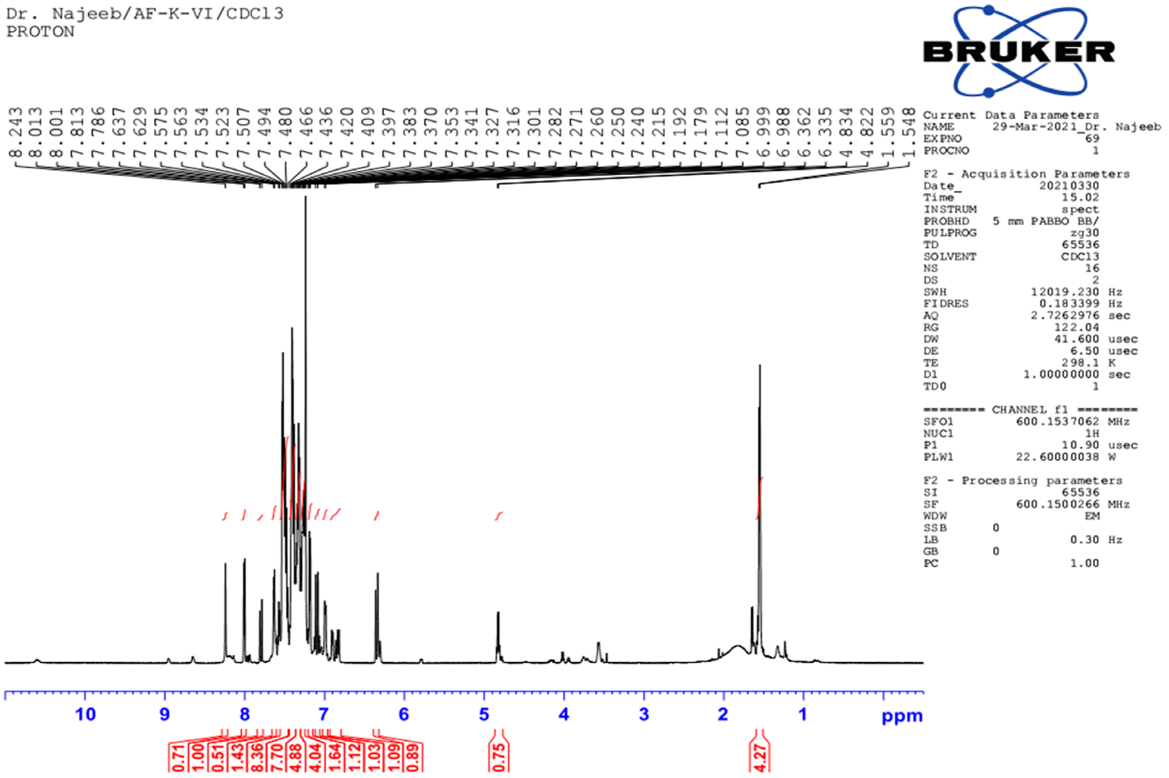

Dr. Najeeb/AF-K-VI/CDCl3
PROTON
BRUKER
8.243 8.013 8.001 7.813 7.786 7.637 7.629 7.575 7.563 7.534 7.523 7.507 7.494 7.480 7.466 7.436 7.420 7.409 7.397 7.383 7.370 7.353 7.341 7.327 7.316 7.301 7.282 7.271 7.260 7.250 7.240 7.215 7.192 7.179 7.112 7.085 6.999 6.988 6.362 6.335 4.834 4.822 1.559 1.548
Current Data Parameters
NAME 29-Mar-2021_Dr. Najeeb
EXPNO 69
PROCNO 1
F2 - Acquisition Parameters
Date_ 20210330
Time 15.02
INSTRUM spect
PROBHD 5 mm PABBO BB/
PULPROG zg30
TD 65536
SOLVENT CDCl3
NS 16
DS 2
SWH 12019.230 Hz
FIDRES 0.183399 Hz
AQ 2.7262976 sec
RG 122.04
DW 41.600 usec
DE 6.50 usec
TE 298.1 K
D1 1.00000000 sec
TD0 1
======== CHANNEL f1 ========
SFO1 600.1537062 MHz
NUC1 1H
P1 10.90 usec
PLW1 22.60000038 W
F2 - Processing parameters
SI 65536
SF 600.1500266 MHz
WDW EM
SSB 0
LB 0.30 Hz
GB 0
PC 1.00
10 9 8 7 6 5 4 3 2 1 ppm
0.71 1.00 0.51 1.43 8.36 7.70 4.88 4.04 1.64 1.12 1.03 1.09 0.89 0.75 4.27

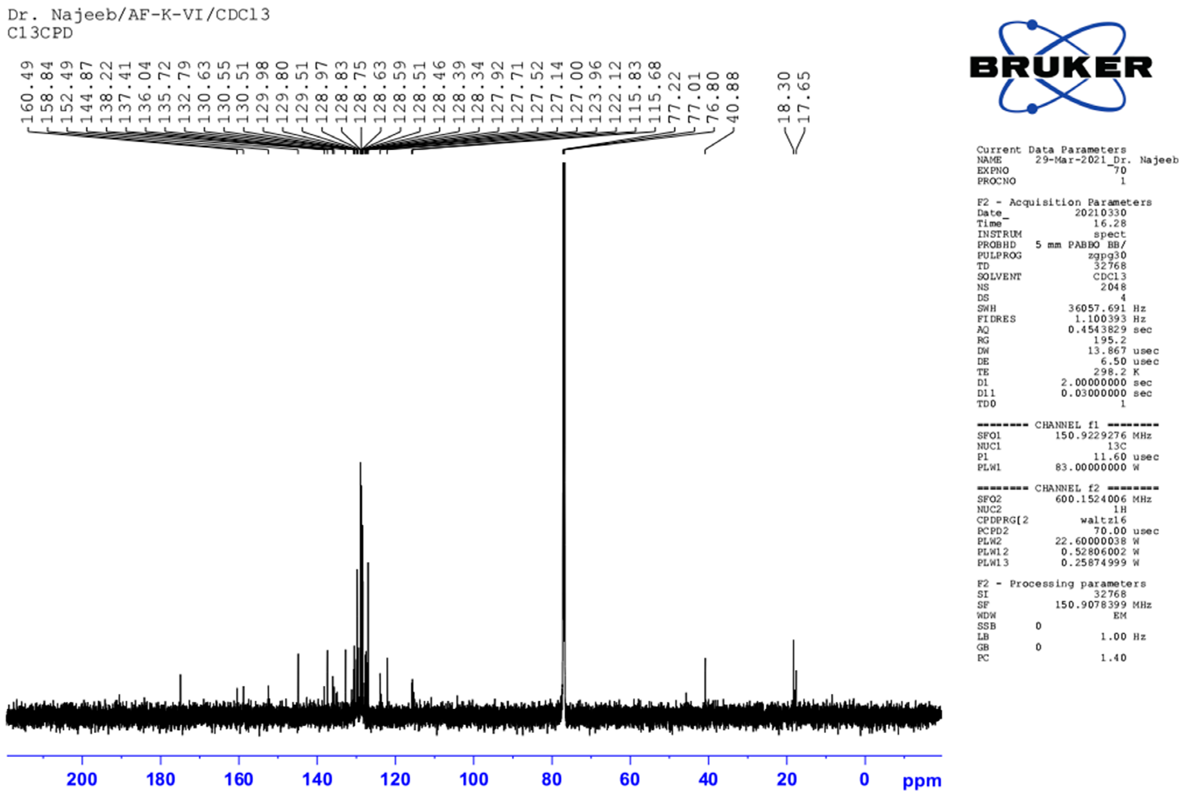

Dr. Najeeb/AF-K-VI/CDCl3
C13CPD
BRUKER
160.49 158.84 152.49 144.87 138.22 137.41 136.04 135.72 132.79 130.63 130.55 130.51 129.98 129.80 129.51 128.97 128.83 128.75 128.63 128.59 128.51 128.46 128.39 128.34 127.92 127.71 127.52 127.14 127.00 123.96 122.12 115.83 115.68 77.22 77.01 76.80 40.88 18.30 17.65
Current Data Parameters
NAME 29-Mar-2021_Dr. Najeeb
EXPNO 70
PROCNO 1
F2 - Acquisition Parameters
Date_ 20210330
Time 16.28
INSTRUM spect
PROBHD 5 mm PABBO BB/
PULPROG zgpg30
TD 32768
SOLVENT CDCl3
NS 2048
DS 4
SWH 36057.691 Hz
FIDRES 1.100393 Hz
AQ 0.4543829 sec
RG 195.2
DW 13.867 usec
DE 6.50 usec
TE 298.2 K
D1 2.00000000 sec
D11 0.03000000 sec
TD0 1
======== CHANNEL f1 ========
SFO1 150.9229276 MHz
NUC1 13C
P1 11.60 usec
PLW1 83.00000000 W
======== CHANNEL f2 ========
SFO2 600.1524006 MHz
NUC2 1H
CPDPRG[2 waltz16
PCPD2 70.00 usec
PLW2 22.60000038 W
PLW12 0.52806002 W
PLW13 0.25874999 W
F2 - Processing parameters
SI 32768
SF 150.9078399 MHz
WDW EM
SSB 0
LB 1.00 Hz
GB 0
PC 1.40
200 180 160 140 120 100 80 60 40 20 0 ppm

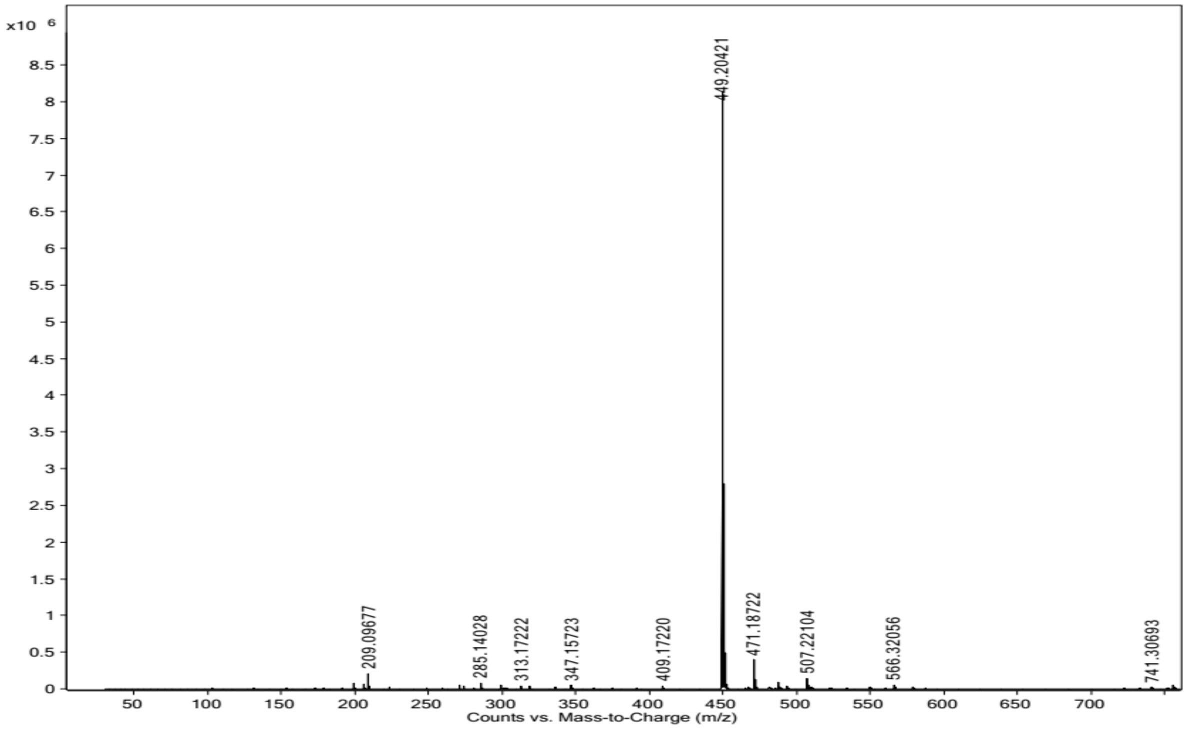

Figure S21: ^{1}H, ^{13}C NMR and HRMS (ESI^{+}) of compound **5e**

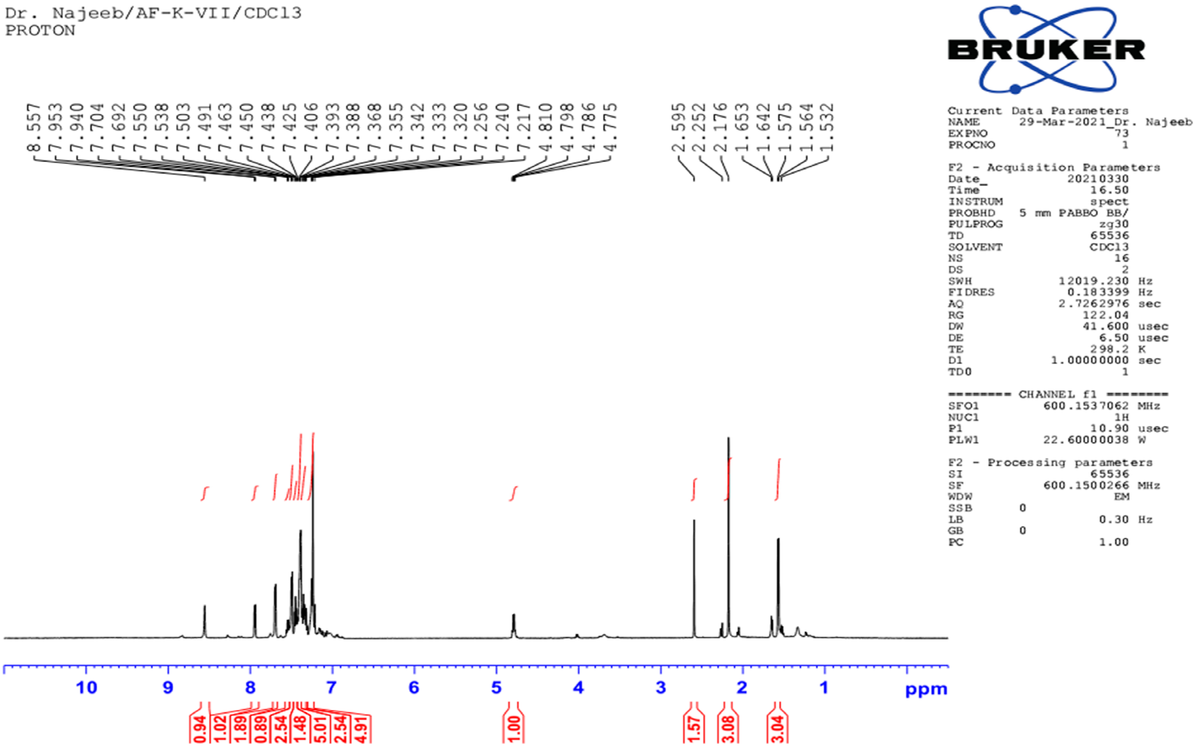

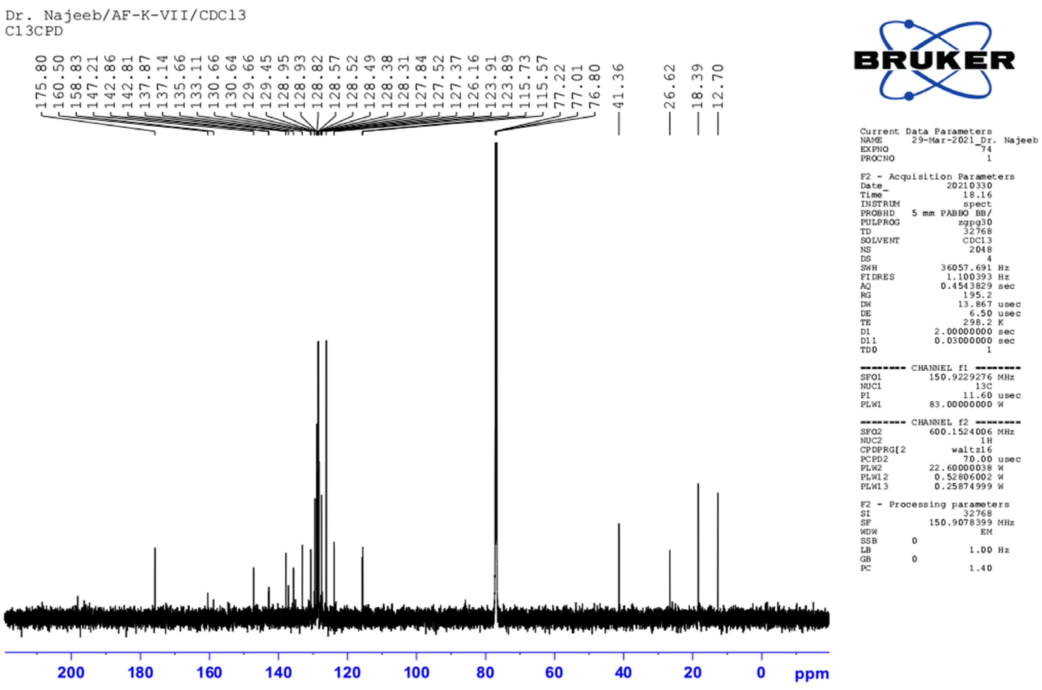

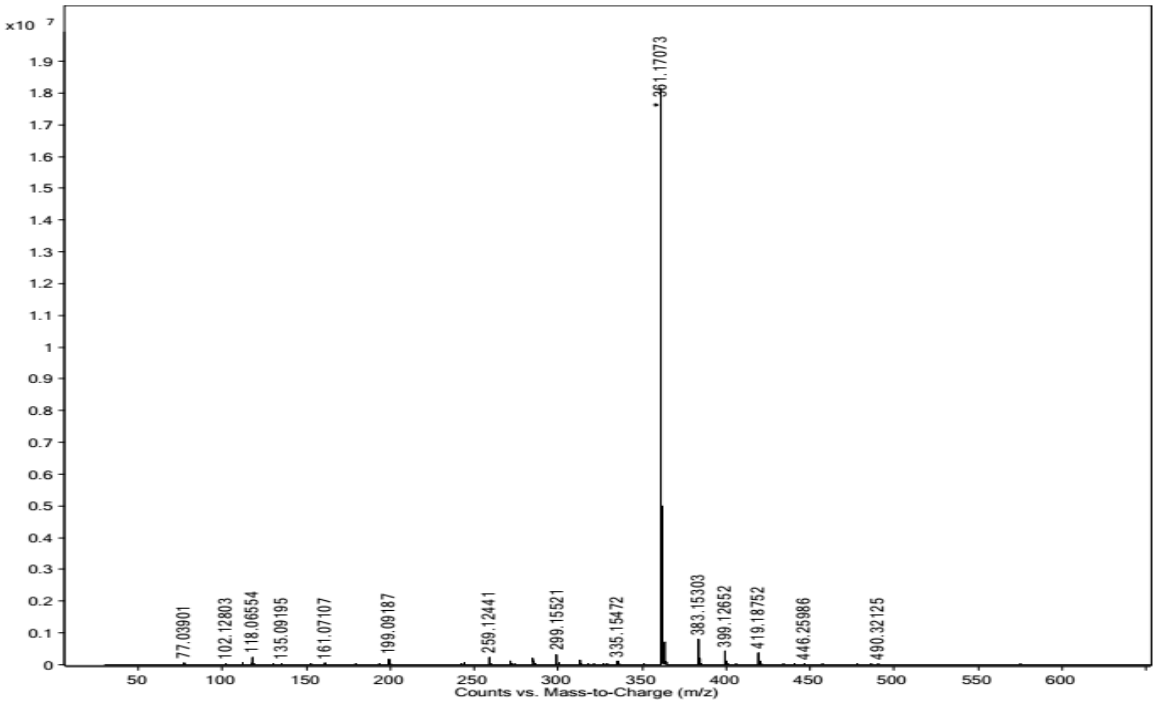

Figure S22: ^{1}H, ^{13}C NMR and HRMS (ESI^{+}) of compound **5f**

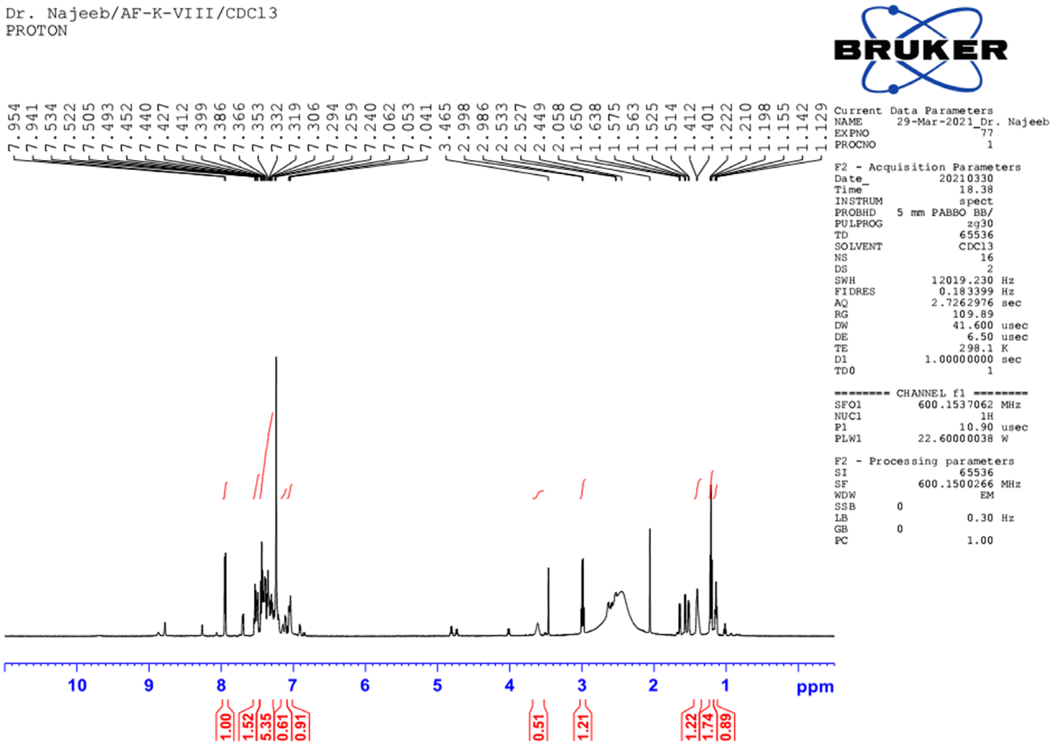
Dr. Najeeb/AF-K-VIII/CDCl3
PROTON
BRUKER
7.954
7.941
7.534
7.522
7.505
7.493
7.452
7.440
7.427
7.412
7.399
7.386
7.366
7.353
7.332
7.319
7.306
7.294
7.259
7.240
7.062
7.053
7.041
3.465
2.998
2.986
2.533
2.527
2.449
2.058
1.650
1.638
1.575
1.563
1.525
1.514
1.412
1.401
1.222
1.210
1.198
1.155
1.142
1.129
Current Data Parameters
NAME 29-Mar-2021_Dr. Najeeb
EXPNO 77
PROCNO 1
F2 - Acquisition Parameters
Date_ 20210330
Time 18.38
INSTRUM spect
PROBHD 5 mm PABBO BB/
PULPROG zg30
TD 65536
SOLVENT CDCl3
NS 16
DS 2
SWH 12019.230 Hz
FIDRES 0.183399 Hz
AQ 2.7262976 sec
RG 109.89
DW 41.600 usec
DE 6.50 usec
TE 298.1 K
D1 1.00000000 sec
TD0 1
======== CHANNEL f1 ========
SFO1 600.1537062 MHz
NUC1 1H
P1 10.90 usec
PLW1 22.60000038 W
F2 - Processing parameters
SI 65536
SF 600.1500266 MHz
WDW EM
SSB 0
LB 0.30 Hz
GB 0
PC 1.00
10
9
8
7
6
5
4
3
2
1
ppm
1.00
1.52
5.35
0.61
0.91
0.51
1.21
1.22
1.74
0.89

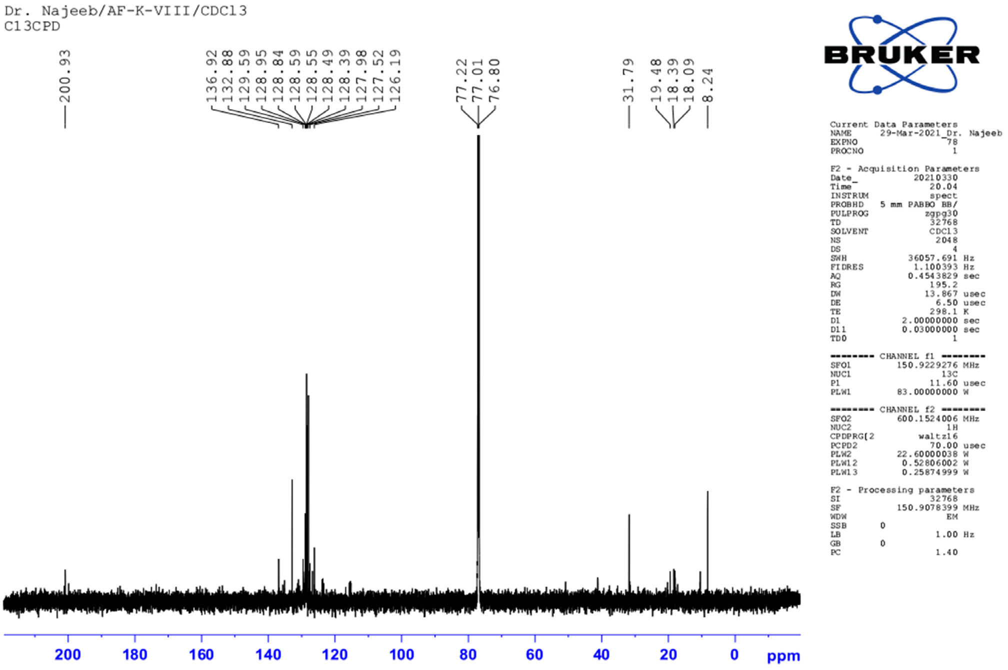
Dr. Najeeb/AF-K-VIII/CDCl3
C13CPD
BRUKER
200.93
136.92
132.88
129.59
128.95
128.84
128.59
128.55
128.49
128.39
127.98
127.52
126.19
77.22
77.01
76.80
31.79
19.48
18.39
18.09
8.24
Current Data Parameters
NAME 29-Mar-2021_Dr. Najeeb
EXPNO 78
PROCNO 1
F2 - Acquisition Parameters
Date_ 20210330
Time 20.04
INSTRUM spect
PROBHD 5 mm PABBO BB/
PULPROG zgpg30
TD 32768
SOLVENT CDCl3
NS 2048
DS 4
SWH 36057.691 Hz
FIDRES 1.100393 Hz
AQ 0.4543829 sec
RG 195.2
DW 13.867 usec
DE 6.50 usec
TE 298.1 K
D1 2.00000000 sec
D11 0.03000000 sec
TD0 1
======== CHANNEL f1 ========
SFO1 150.9229276 MHz
NUC1 13C
P1 11.60 usec
PLW1 83.00000000 W
======== CHANNEL f2 ========
SFO2 600.1524006 MHz
NUC2 1H
CPDPRG[2 waltz16
PCPD2 70.00 usec
PLW2 22.60000038 W
PLW12 0.52806002 W
PLW13 0.25874999 W
F2 - Processing parameters
SI 32768
SF 150.9078399 MHz
WDW EM
SSB 0
LB 1.00 Hz
GB 0
PC 1.40
200
180
160
140
120
100
80
60
40
20
0
ppm

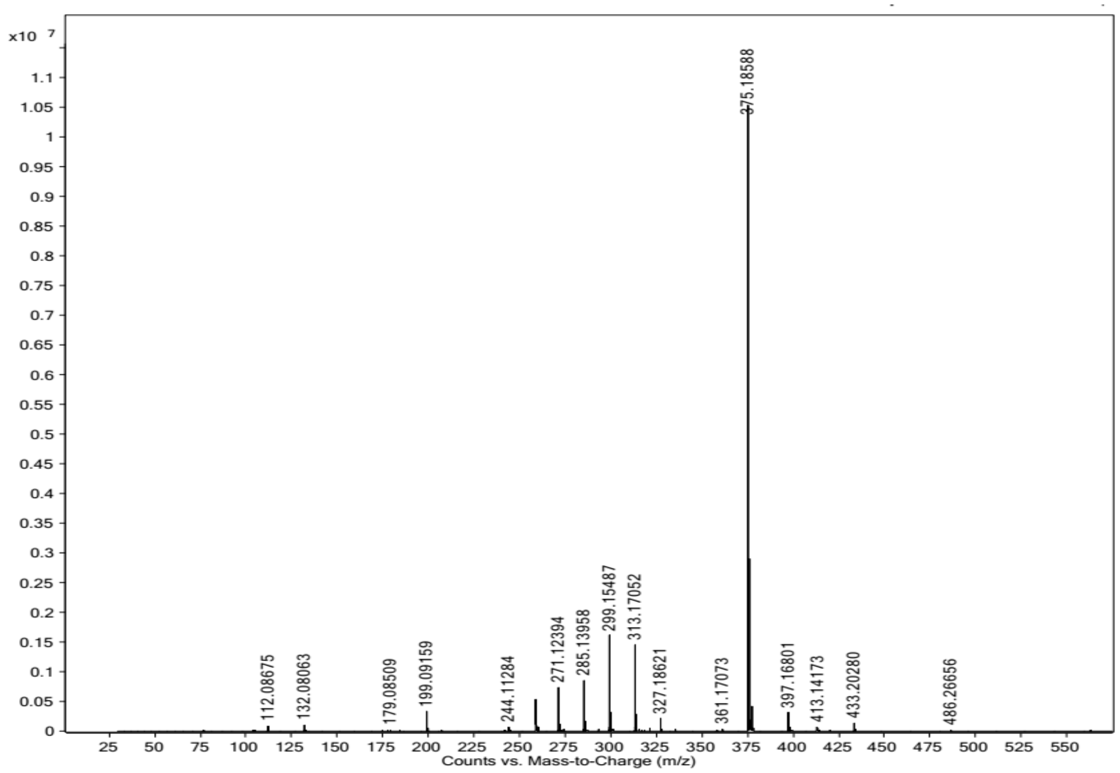

Figure S23: ^{1}H, ^{13}C NMR and HRMS (ESI^{+}) of compound **5g**

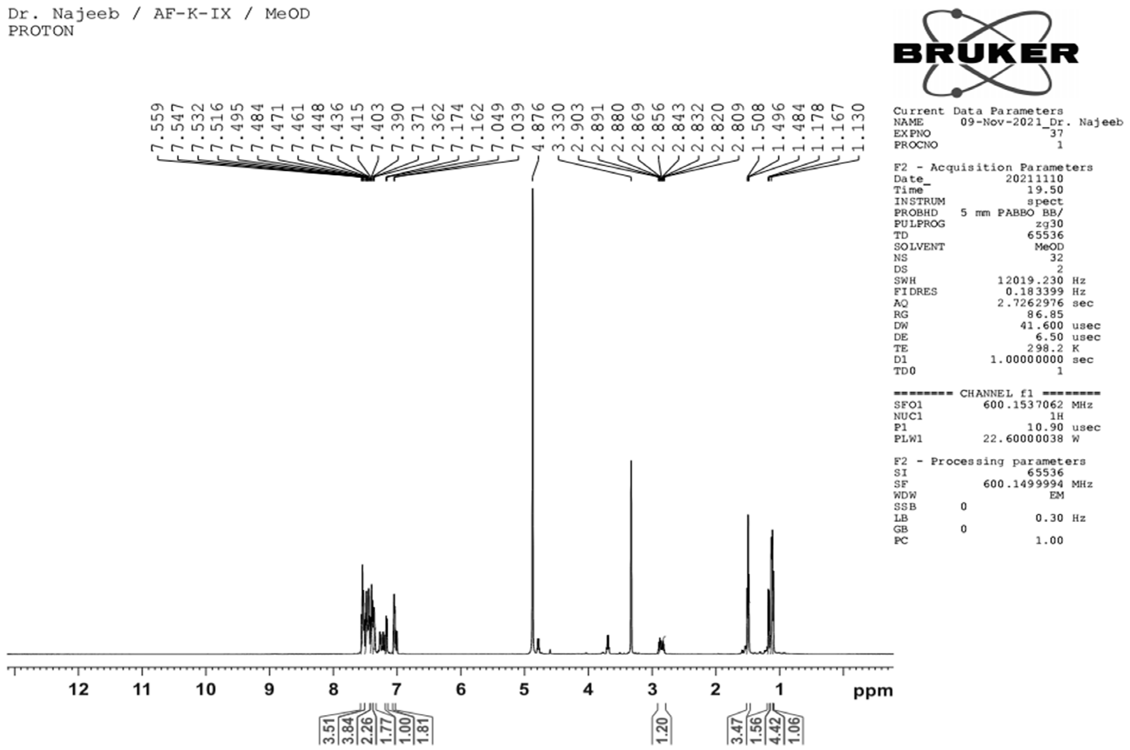

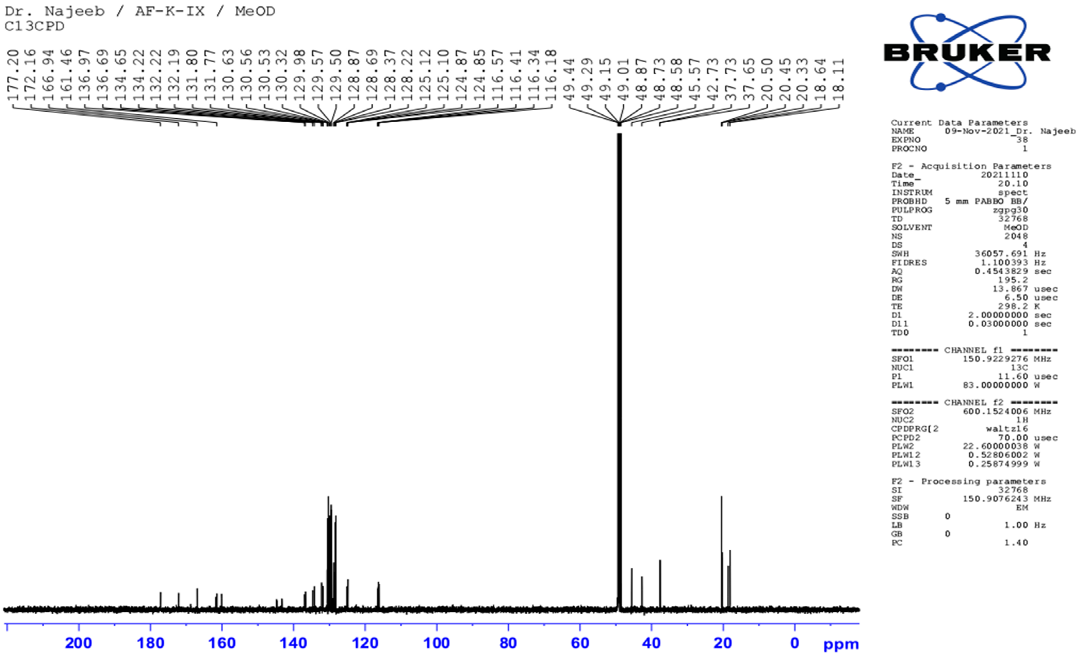

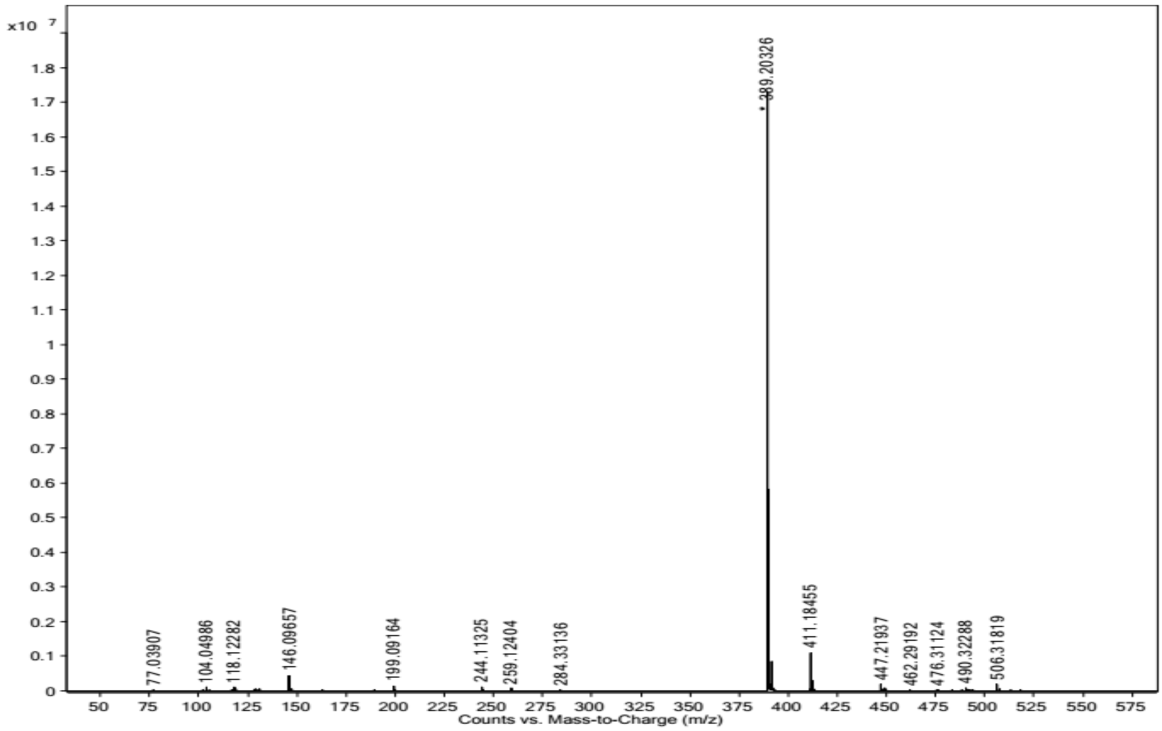

Figure S24: ^{1}H, ^{13}C NMR and HRMS (ESI[+]) of compound **5h**

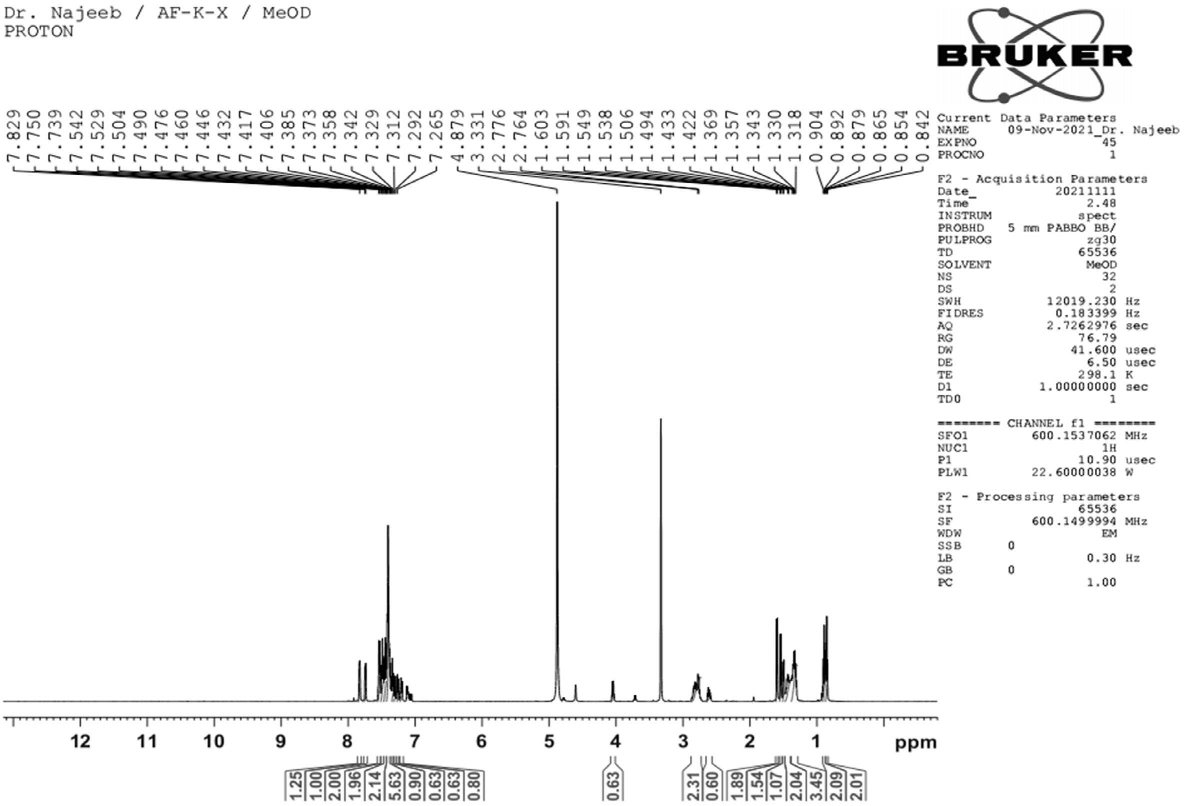
Dr. Najeeb / AF-K-X / MeOD
PROTON
7.829 7.750 7.739 7.542 7.529 7.504 7.490 7.476 7.460 7.446 7.432 7.417 7.406 7.385 7.373 7.358 7.342 7.329 7.312 7.292 7.265 4.879 3.331 2.776 2.764 1.603 1.591 1.549 1.538 1.506 1.494 1.433 1.422 1.369 1.357 1.343 1.330 1.318 0.904 0.892 0.879 0.865 0.854 0.842
BRUKER
Current Data Parameters
NAME 09-Nov-2021_Dr. Najeeb
EXPNO 45
PROCNO 1
F2 - Acquisition Parameters
Date_ 20211111
Time 2.48
INSTRUM spect
PROBHD 5 mm PABBO BB/
PULPROG zg30
TD 65536
SOLVENT MeOD
NS 32
DS 2
SWH 12019.230 Hz
FIDRES 0.183399 Hz
AQ 2.7262976 sec
RG 76.79
DW 41.600 usec
DE 6.50 usec
TE 298.1 K
D1 1.00000000 sec
TD0 1
======== CHANNEL f1 ========
SFO1 600.1537062 MHz
NUC1 1H
P1 10.90 usec
PLW1 22.60000038 W
F2 - Processing parameters
SI 65536
SF 600.1499994 MHz
WDW EM
SSB 0
LB 0.30 Hz
GB 0
PC 1.00
12 11 10 9 8 7 6 5 4 3 2 1 ppm
1.25 1.00 2.00 1.96 2.14 5.63 0.90 0.63 0.63 0.80 0.63 2.31 0.60 1.89 1.54 1.07 2.04 3.45 2.09 2.01

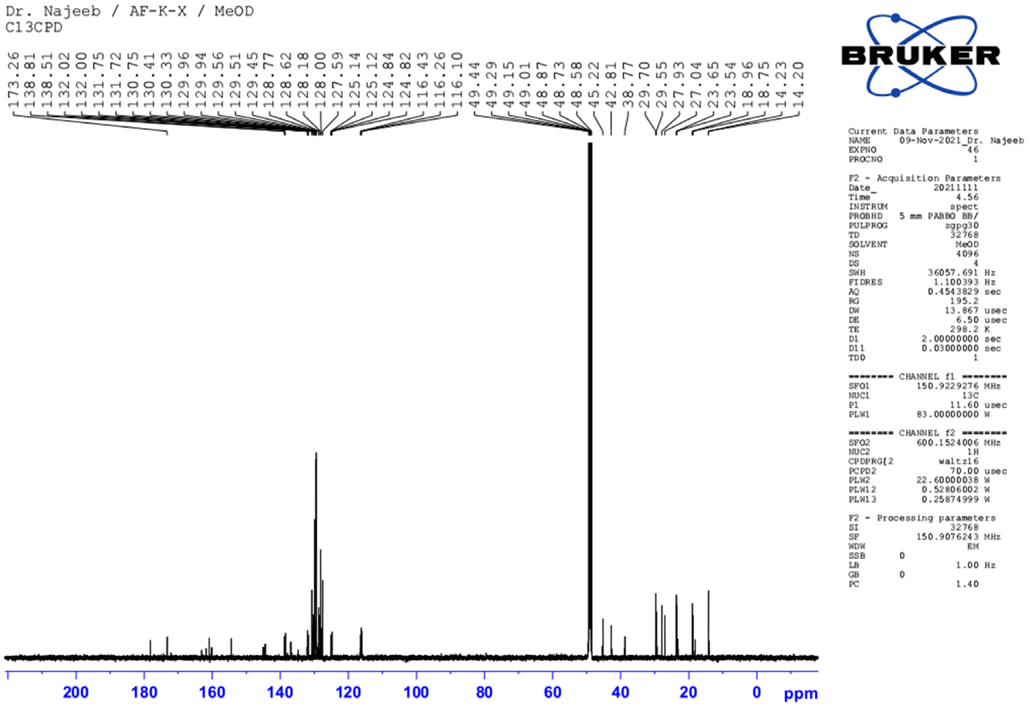
Dr. Najeeb / AF-K-X / MeOD
C13CPD
173.26 138.81 138.51 132.02 132.00 131.75 131.72 130.75 130.41 130.33 129.96 129.94 129.56 129.51 129.45 128.77 128.62 128.18 128.00 127.59 125.14 125.12 124.84 124.82 116.43 116.26 116.10 49.44 49.29 49.15 49.01 48.87 48.73 48.58 45.22 42.81 38.77 29.70 29.55 27.93 27.04 23.65 23.54 18.96 18.75 14.23 14.20
BRUKER
Current Data Parameters
NAME 09-Nov-2021_Dr. Najeeb
EXPNO 46
PROCNO 1
F2 - Acquisition Parameters
Date_ 20211111
Time 4.56
INSTRUM spect
PROBHD 5 mm PABBO BB/
PULPROG zgpg30
TD 32768
SOLVENT MeOD
NS 4096
DS 4
SWH 36057.691 Hz
FIDRES 1.100393 Hz
AQ 0.4543829 sec
RG 195.2
DW 13.867 usec
DE 6.50 usec
TE 298.2 K
D1 2.00000000 sec
D11 0.03000000 sec
TD0 1
======== CHANNEL f1 ========
SFO1 150.9229276 MHz
NUC1 13C
P1 11.60 usec
PLW1 83.00000000 W
======== CHANNEL f2 ========
SFO2 600.1524006 MHz
NUC2 1H
CPDPRG[2 waltz16
PCPD2 70.00 usec
PLW2 22.60000038 W
PLW12 0.52806002 W
PLW13 0.25874999 W
F2 - Processing parameters
SI 32768
SF 150.9076243 MHz
WDW EM
SSB 0
LB 1.00 Hz
GB 0
PC 1.40
200 180 160 140 120 100 80 60 40 20 0 ppm

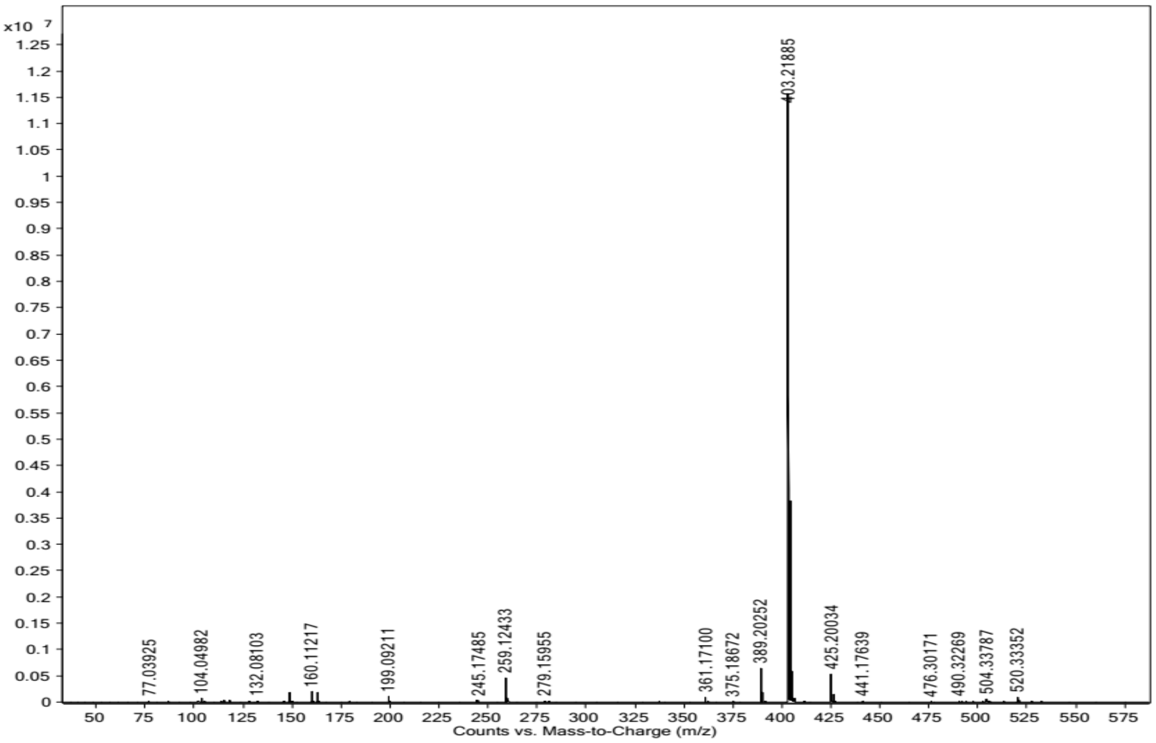

Figure S25: ^{1}H, ^{13}C NMR and HRMS (ESI^{+}) of compound **5i**

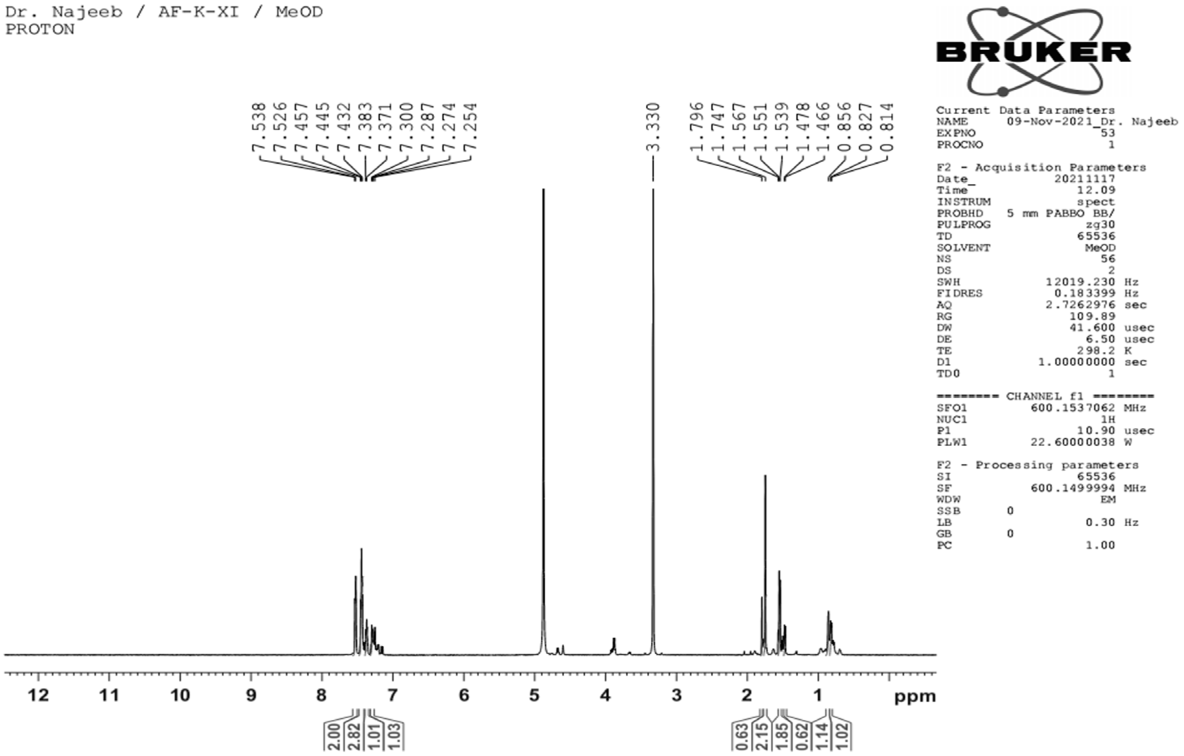

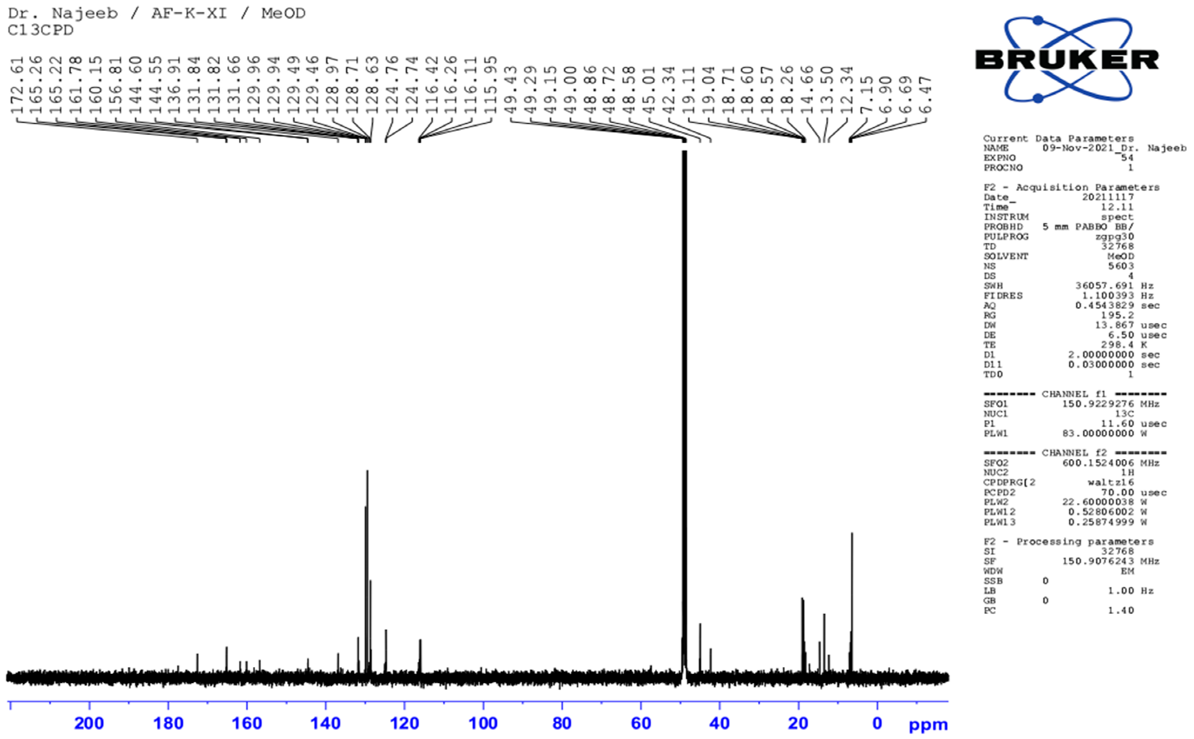

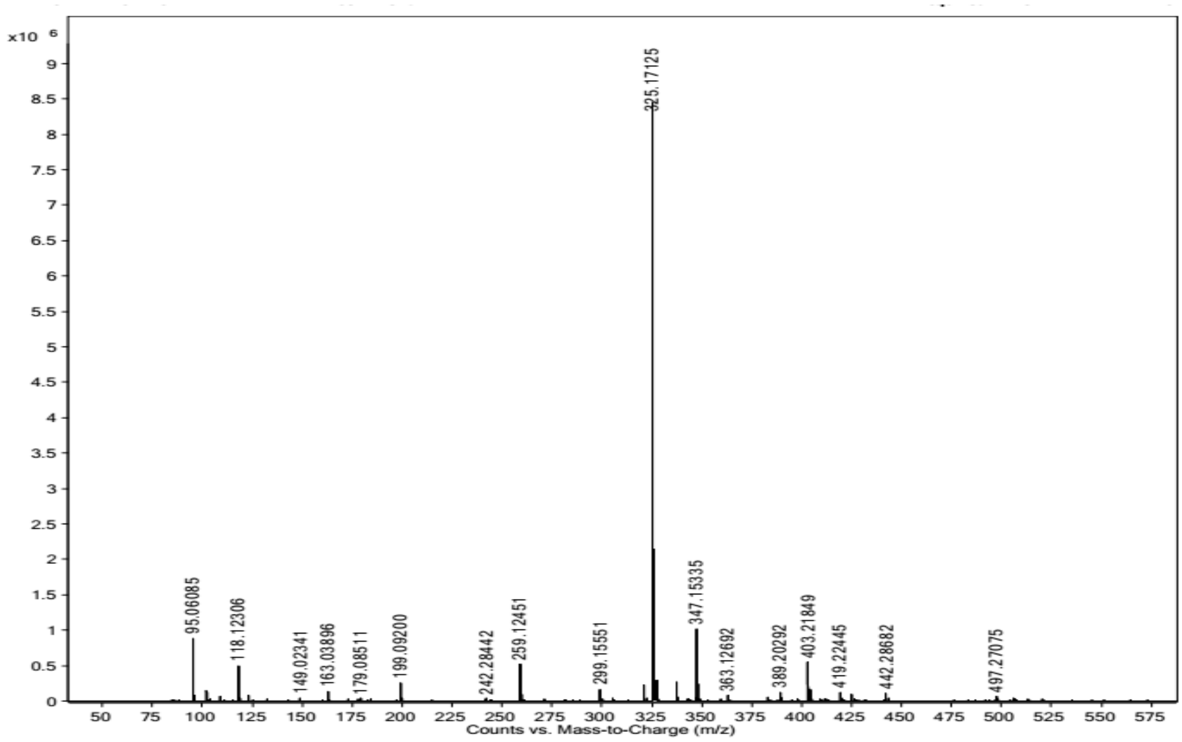

Figure S26: ^{1}H, ^{13}C NMR and HRMS (ESI^{+}) of compound **5j**

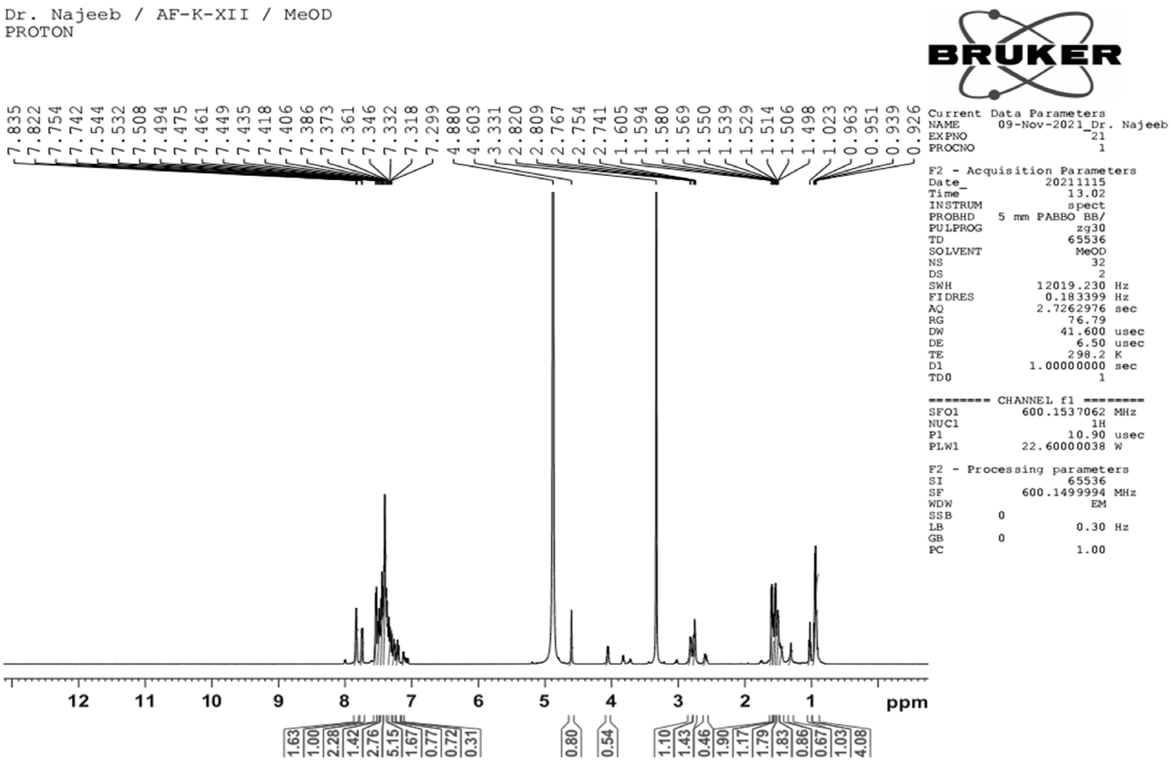
Dr. Najeeb / AF-K-XII / MeOD
PROTON
BRUKER
Current Data Parameters
NAME 09-Nov-2021_Dr. Najeeb
EXPNO 21
PROCNO 1
F2 - Acquisition Parameters
Date_ 20211115
Time 13.02
INSTRUM spect
PROBHD 5 mm PABBO BB/
PULPROG zg30
TD 65536
SOLVENT MeOD
NS 32
DS 2
SWH 12019.230 Hz
FIDRES 0.183399 Hz
AQ 2.7262976 sec
RG 76.79
DW 41.600 usec
DE 6.50 usec
TE 298.2 K
D1 1.00000000 sec
TD0 1
======== CHANNEL f1 ========
SFO1 600.1537062 MHz
NUC1 1H
P1 10.90 usec
PLW1 22.60000038 W
F2 - Processing parameters
SI 65536
SF 600.1499994 MHz
WDW EM
SSB 0
LB 0.30 Hz
GB 0
PC 1.00
ppm

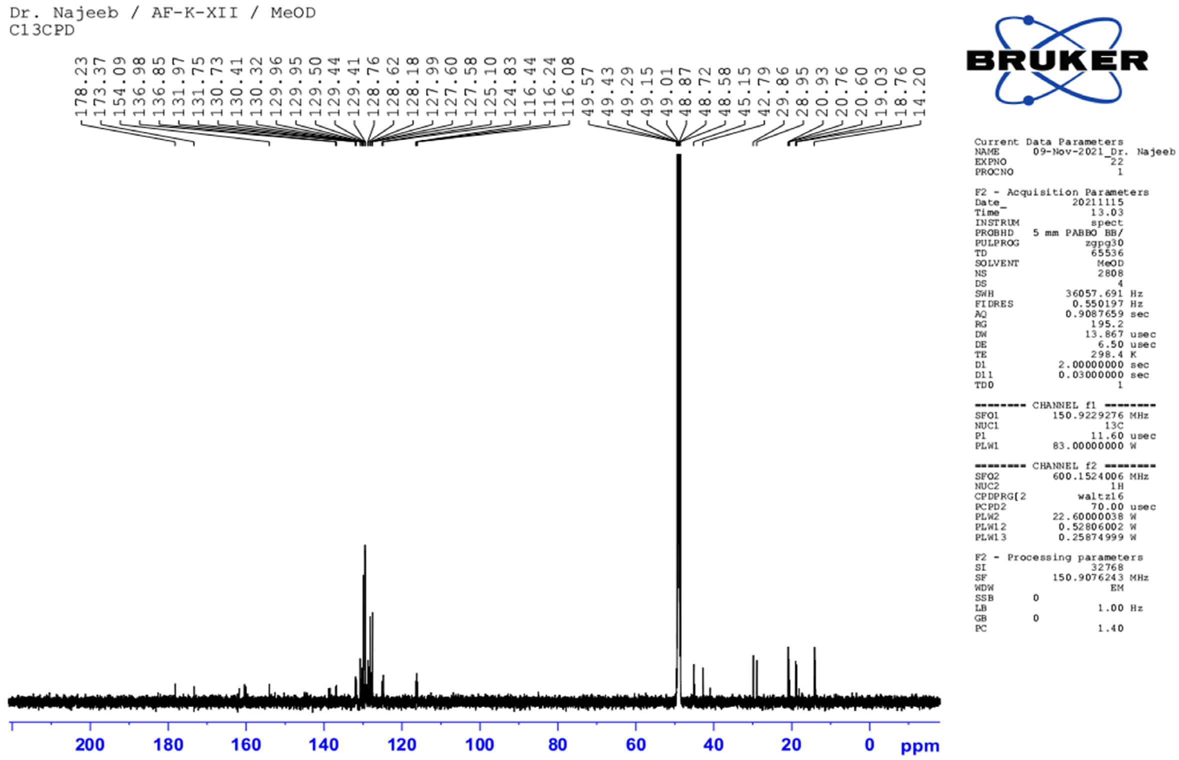
Dr. Najeeb / AF-K-XII / MeOD
C13CPD
BRUKER
Current Data Parameters
NAME 09-Nov-2021_Dr. Najeeb
EXPNO 22
PROCNO 1
F2 - Acquisition Parameters
Date_ 20211115
Time 13.03
INSTRUM spect
PROBHD 5 mm PABBO BB/
PULPROG zgpg30
TD 65536
SOLVENT MeOD
NS 2808
DS 4
SWH 36057.691 Hz
FIDRES 0.550197 Hz
AQ 0.9087659 sec
RG 195.2
DW 13.867 usec
DE 6.50 usec
TE 298.4 K
D1 2.00000000 sec
D11 0.03000000 sec
TD0 1
======== CHANNEL f1 ========
SFO1 150.9229276 MHz
NUC1 13C
P1 11.60 usec
PLW1 83.00000000 W
======== CHANNEL f2 ========
SFO2 600.1524006 MHz
NUC2 1H
CPDPRG[2 waltz16
PCPD2 70.00 usec
PLW2 22.60000038 W
PLW12 0.52806002 W
PLW13 0.25874999 W
F2 - Processing parameters
SI 32768
SF 150.9076243 MHz
WDW EM
SSB 0
LB 1.00 Hz
GB 0
PC 1.40
ppm

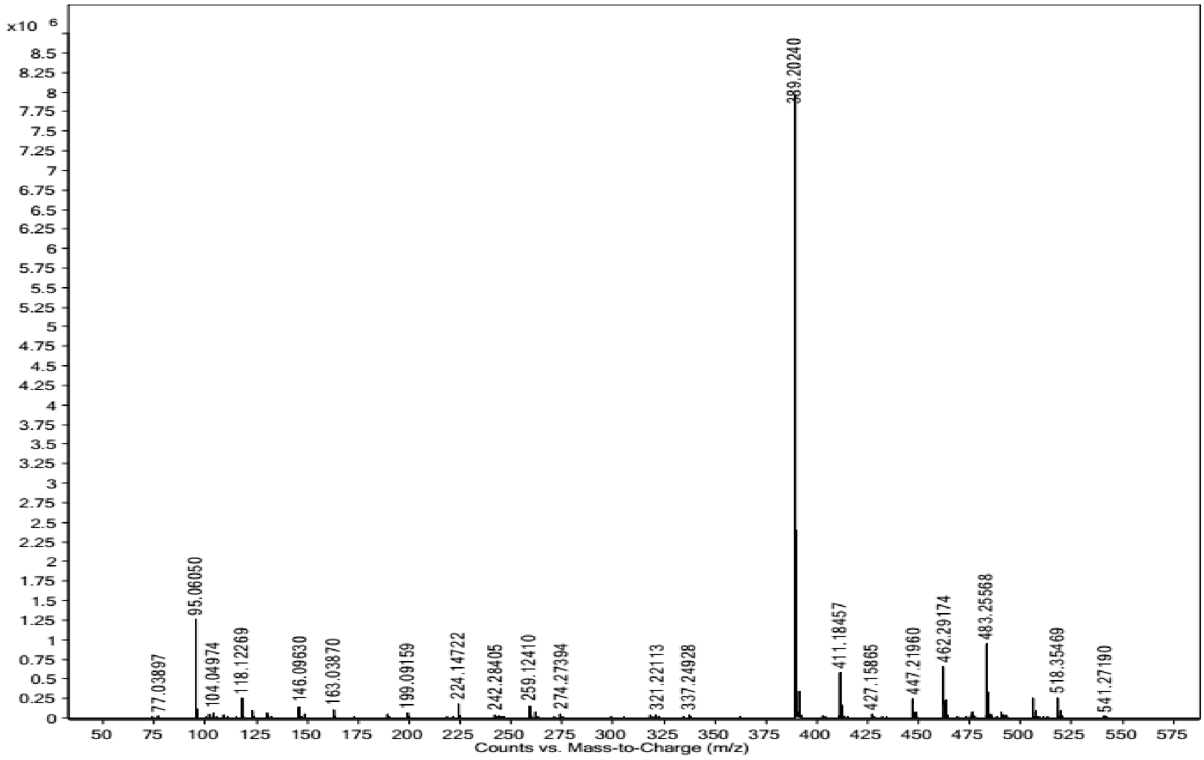

Figure S27: ^{1}H, ^{13}C NMR and HRMS (ESI^{+}) of compound **5k**

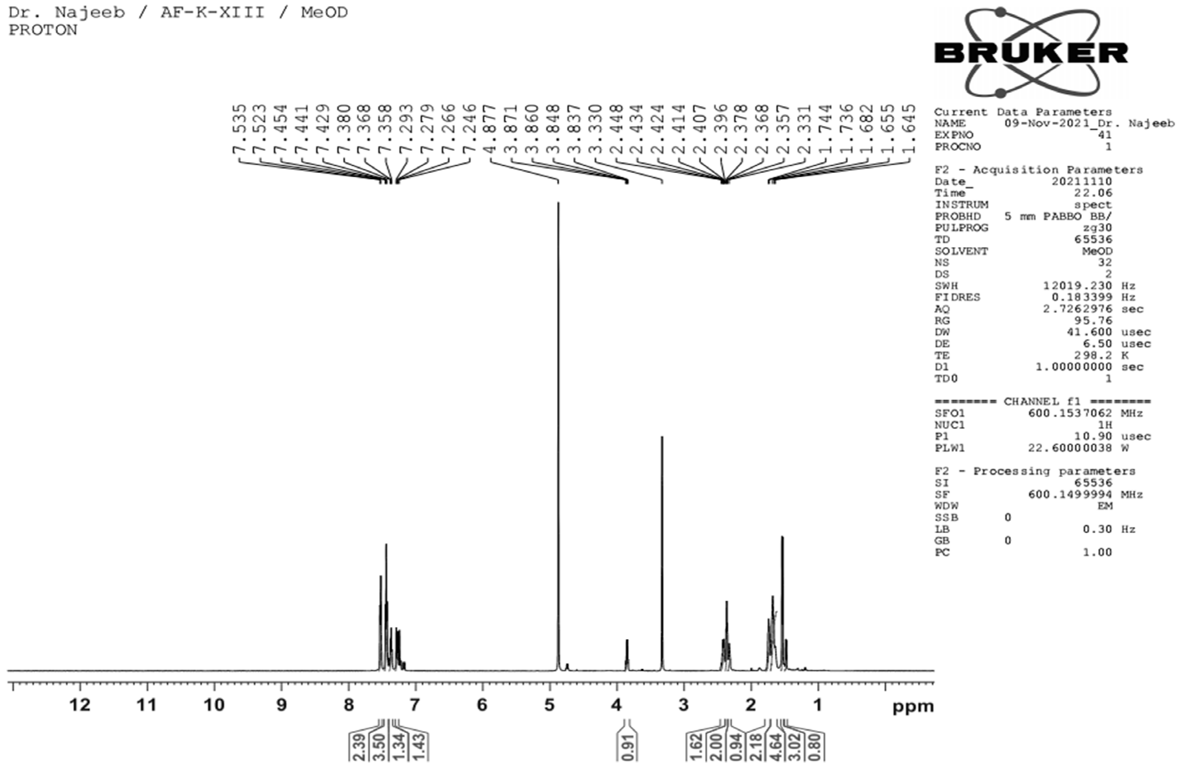

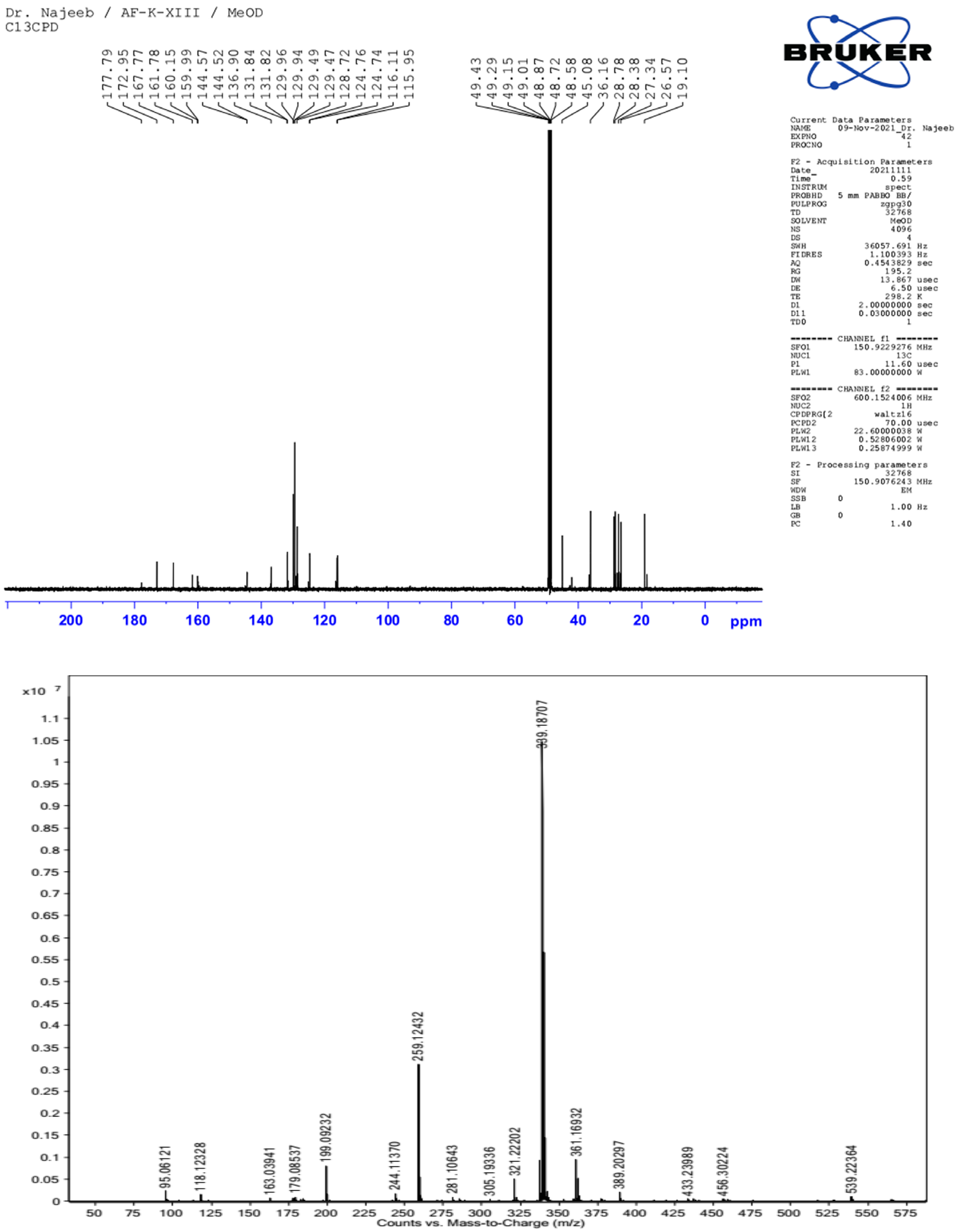

Figure S28: ^{1}H, ^{13}C NMR and HRMS (ESI^{+}) of compound **5l**

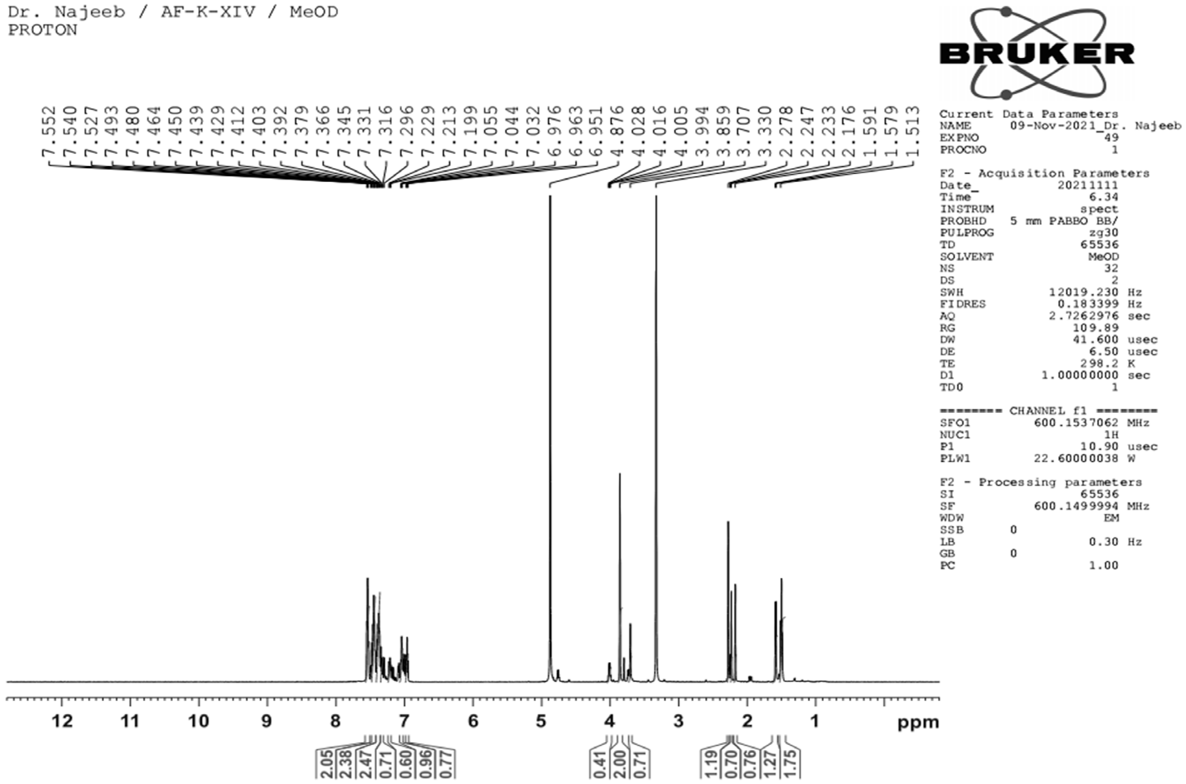
Dr. Najeeb / AF-K-XIV / MeOD
PROTON
BRUKER
7.552 7.540 7.527 7.493 7.480 7.464 7.450 7.439 7.429 7.412 7.403 7.392 7.379 7.366 7.345 7.331 7.316 7.296 7.229 7.213 7.199 7.055 7.044 7.032 6.976 6.963 6.951 4.876 4.028 4.016 4.005 3.994 3.859 3.707 3.330 2.278 2.247 2.233 2.176 1.591 1.579 1.513
Current Data Parameters
NAME 09-Nov-2021_Dr. Najeeb
EXPNO 49
PROCNO 1
F2 - Acquisition Parameters
Date_ 20211111
Time 6.34
INSTRUM spect
PROBHD 5 mm PABBO BB/
PULPROG zg30
TD 65536
SOLVENT MeOD
NS 32
DS 2
SWH 12019.230 Hz
FIDRES 0.183399 Hz
AQ 2.7262976 sec
RG 109.89
DW 41.600 usec
DE 6.50 usec
TE 298.2 K
D1 1.00000000 sec
TD0 1
======= CHANNEL f1 =======
SFO1 600.1537062 MHz
NUC1 1H
P1 10.90 usec
PLW1 22.60000038 W
F2 - Processing parameters
SI 65536
SF 600.1499994 MHz
WDW EM
SSB 0
LB 0.30 Hz
GB 0
PC 1.00
12 11 10 9 8 7 6 5 4 3 2 1 ppm
2.05 2.38 2.47 0.71 0.60 0.96 0.77 0.41 2.00 0.71 1.19 0.70 0.76 1.27 1.75

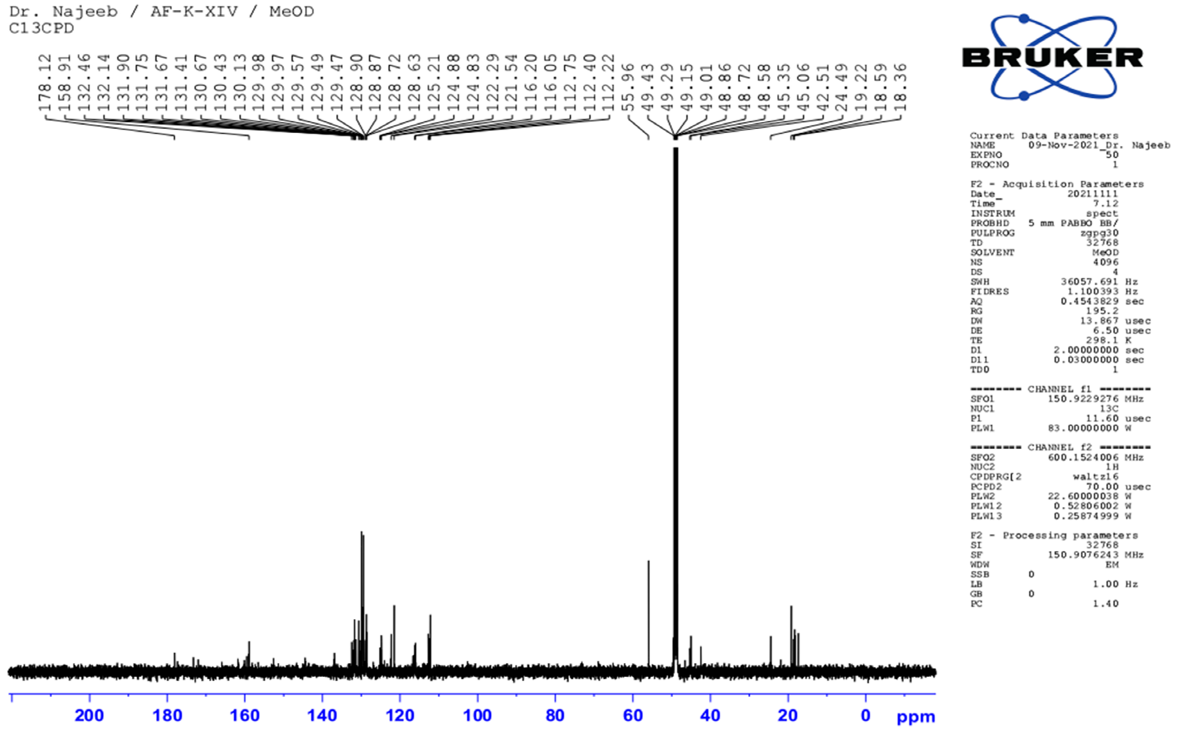
Dr. Najeeb / AF-K-XIV / MeOD
C13CPD
BRUKER
178.12 158.91 132.46 132.14 131.90 131.75 131.67 131.41 130.67 130.43 130.13 129.98 129.97 129.57 129.49 129.47 128.90 128.87 128.72 128.63 125.21 124.88 124.83 122.29 121.54 116.20 116.05 112.75 112.40 112.22 55.96 49.43 49.29 49.15 49.01 48.86 48.72 48.58 45.35 45.06 42.51 24.49 19.22 18.59 18.36
Current Data Parameters
NAME 09-Nov-2021_Dr. Najeeb
EXPNO 50
PROCNO 1
F2 - Acquisition Parameters
Date_ 20211111
Time 7.12
INSTRUM spect
PROBHD 5 mm PABBO BB/
PULPROG zgpg30
TD 32768
SOLVENT MeOD
NS 4096
DS 4
SWH 36057.691 Hz
FIDRES 1.100393 Hz
AQ 0.4543829 sec
RG 195.2
DW 13.867 usec
DE 6.50 usec
TE 298.1 K
D1 2.00000000 sec
D11 0.03000000 sec
TD0 1
======= CHANNEL f1 =======
SFO1 150.9229276 MHz
NUC1 13C
P1 11.60 usec
PLW1 83.00000000 W
======= CHANNEL f2 =======
SFO2 600.1524006 MHz
NUC2 1H
CPDPRG[2 waltz16
PCPD2 70.00 usec
PLW2 22.60000038 W
PLW12 0.52806002 W
PLW13 0.25874999 W
F2 - Processing parameters
SI 32768
SF 150.9076243 MHz
WDW EM
SSB 0
LB 1.00 Hz
GB 0
PC 1.40
200 180 160 140 120 100 80 60 40 20 0 ppm

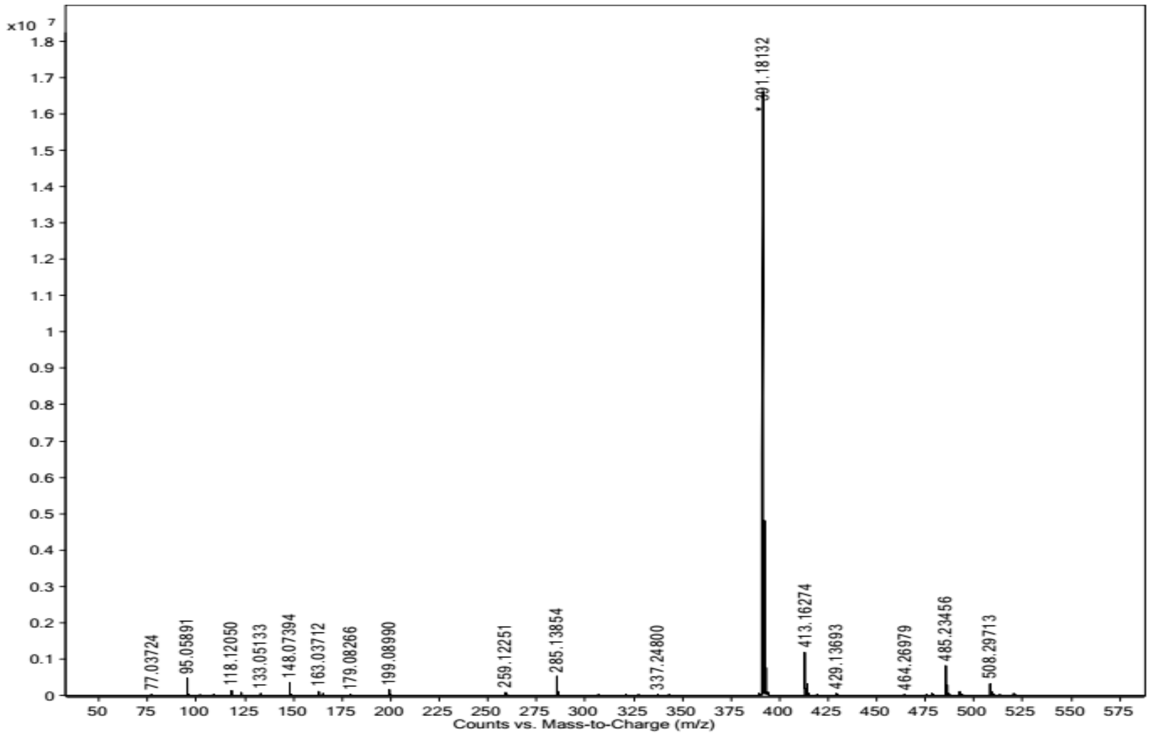

Figure S29: ^{1}H, ^{13}C NMR and HRMS (ESI^{+}) of compound **5m**

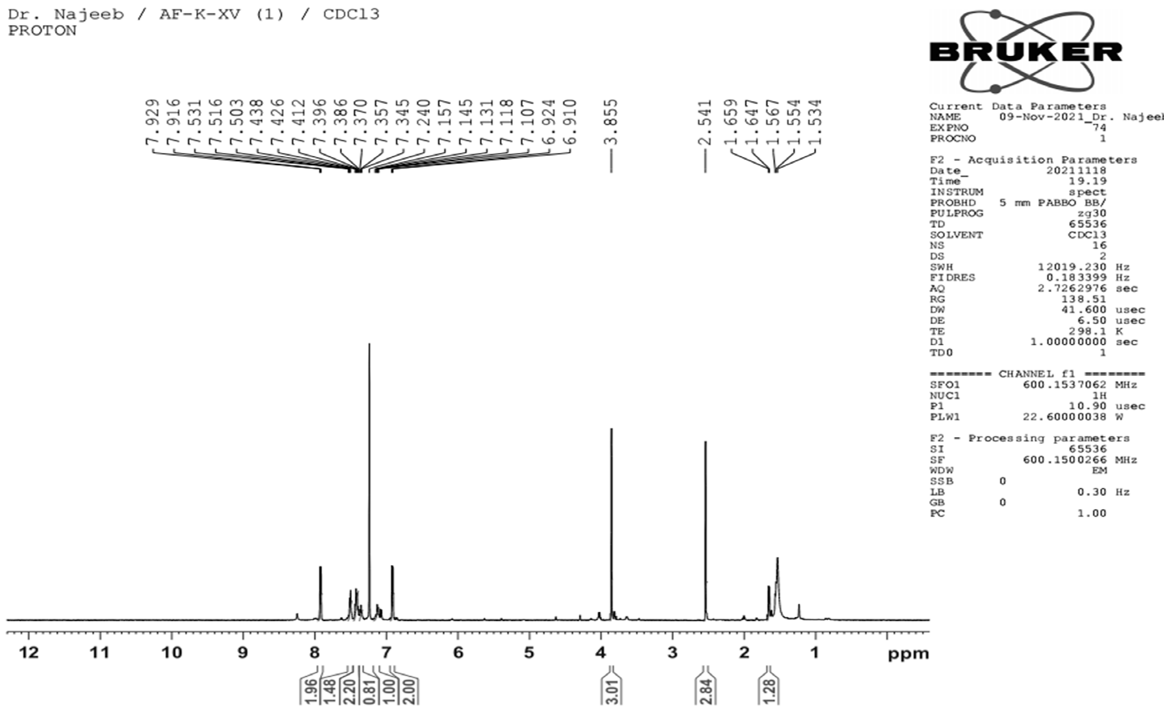

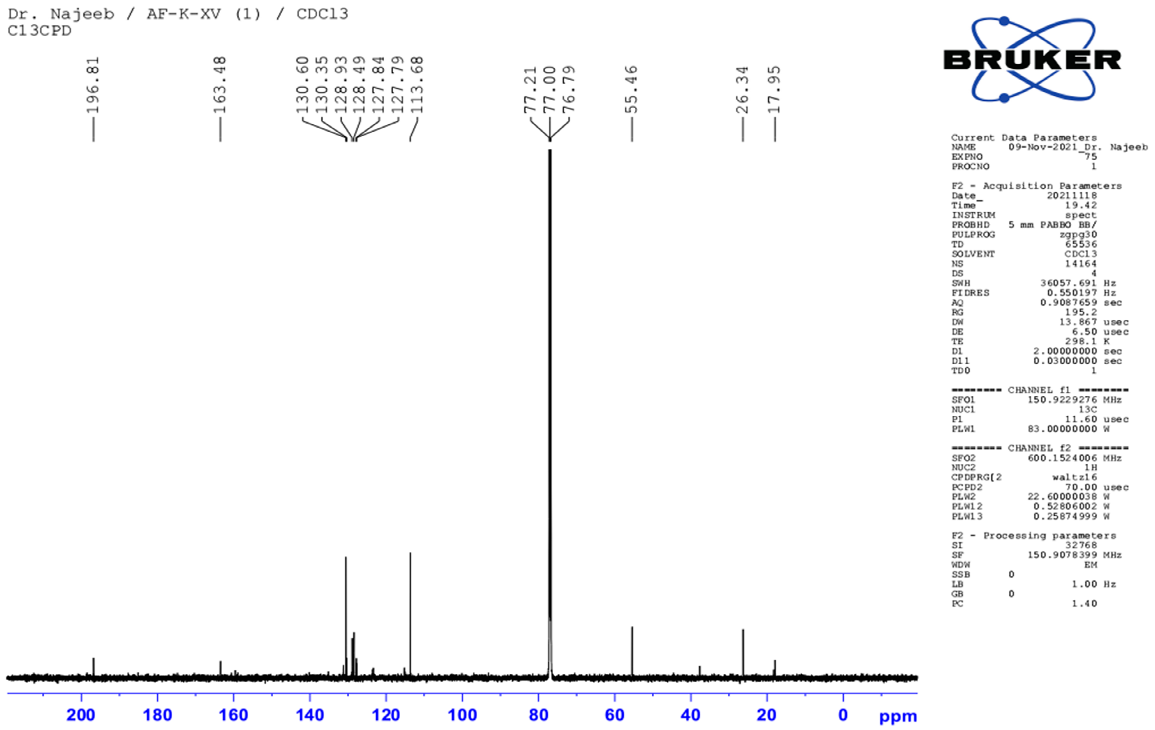

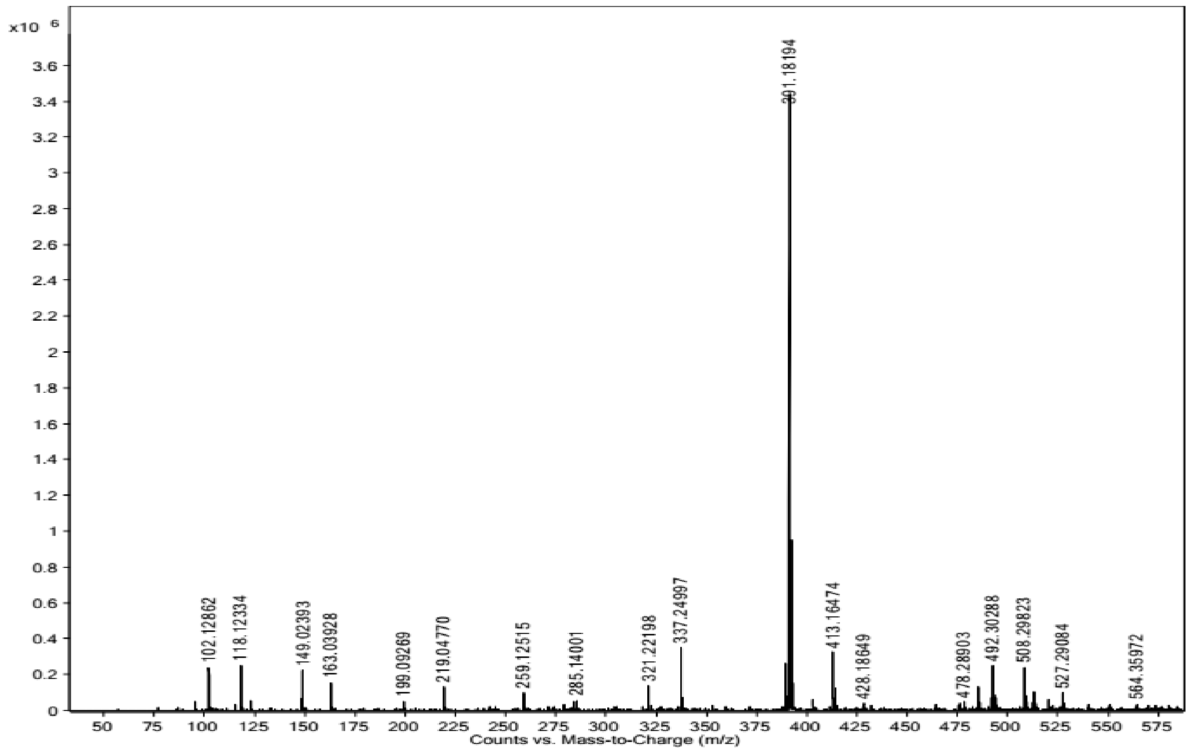

Figure S30: ^{1}H, ^{13}C NMR and HRMS (ESI^+) of compound **5n**
